# The conservation landscape of the human ribosomal RNA gene repeats

**DOI:** 10.1371/journal.pone.0207531

**Published:** 2018-12-05

**Authors:** Saumya Agrawal, Austen R. D. Ganley

**Affiliations:** 1 Institute of Natural and Mathematical Sciences, Massey University, Auckland, New Zealand; 2 School of Biological Sciences, University of Auckland, Auckland, New Zealand; National Center for Biotechnology Information, UNITED STATES

## Abstract

Ribosomal RNA gene repeats (rDNA) encode ribosomal RNA, a major component of ribosomes. Ribosome biogenesis is central to cellular metabolic regulation, and several diseases are associated with rDNA dysfunction, notably cancer, However, its highly repetitive nature has severely limited characterization of the elements responsible for rDNA function. Here we make use of phylogenetic footprinting to provide a comprehensive list of novel, potentially functional elements in the human rDNA. Complete rDNA sequences for six non-human primate species were constructed using *de novo* whole genome assemblies. These new sequences were used to determine the conservation profile of the human rDNA, revealing 49 conserved regions in the rDNA intergenic spacer (IGS). To provide insights into the potential roles of these conserved regions, the conservation profile was integrated with functional genomics datasets. We find two major zones that contain conserved elements characterised by enrichment of transcription-associated chromatin factors, and transcription. Conservation of some IGS transcripts in the apes underpins the potential functional significance of these transcripts and the elements controlling their expression. Our results characterize the conservation landscape of the human IGS and suggest that noncoding transcription and chromatin elements are conserved and important features of this unique genomic region.

## Introduction

**Fig 1:**
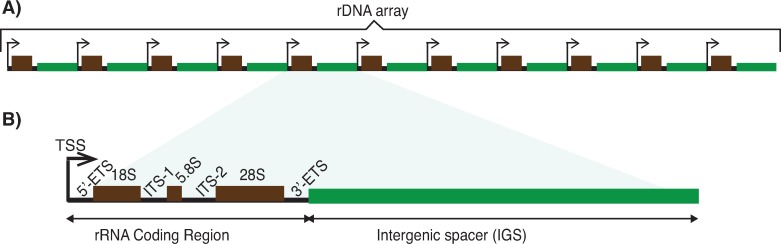
Eukaryotic ribosomal DNA organization. **A**) Head-to-tail tandem arrangement of rDNA repeat units. Typically, there are more units in an array than depicted. **B**) Each rDNA unit has an rRNA coding region (black) and an intergenic spacer (IGS; green). The coding region encodes the ~18S, 5.8S and ~28S rRNAs (black boxes) separated by two internal transcribed spacers (ITS-1 and 2) and flanked by two external transcribed spacers (5’- and 3’-ETS).

A characteristic feature of most eukaryote genomes is the presence of one or more tandem arrays of gene repeats encoding ribosomal RNA (rRNA), a key building block of ribosomes. The major eukaryotic rRNA gene repeat family is known as the ribosomal DNA (rDNA), with each repeat encompassing a coding region encoding 18S, 5.8S and 28S rRNA, and an intergenic spacer (IGS) that separates adjacent coding regions (**Fig 1**). In humans, each repeat unit is ~43 kb in length, with a ~13 kb rRNA coding region and a ~30 kb IGS [1]. There are approximately 200–600 rDNA copies distributed amongst tandem arrays on the short arms of the five acrocentric chromosomes in human (chromosomes 13, 14, 15, 21, and 22) [2–7]. The rDNA is transcribed by RNA Polymerase I (Pol-I) in the nucleolus [8,9], and this primary role in ribosome biogenesis places the rDNA at the heart of cellular metabolic homeostasis [10]. In addition, the rDNA has been found to mediate a number of “extra-coding” functions, including roles in genome stability [11,12], cell cycle control [13–17], protein sequestration [18], epigenetic silencing [19,20], and aging [21,22], and it forms three-dimensional interactions with other areas of the genome [23,24].

A critical outcome of the central role the rDNA plays in the biology of the cell is an association with a number of human diseases. An association between ribosome biogenesis/rDNA and cancer dates back over 100 years and stems from observations of nucleolar hypertrophy and upregulated rRNA expression in tumour cells [25–28]. rRNA dysfunction is also associated with a group of genetic diseases that result from impaired ribosome biogenesis, known as ribosomopathies [29,30]. In addition, there is growing evidence for the rDNA playing a role in cellular differentiation [31–34]. Despite these strong connections to human pathology, the rDNA remains poorly characterized [35]. Critically, the rDNA is not placed in the human genome chromosomal assembly [36], and consequently is excluded from many genome-wide analyses. Recent studies have begun using genomic data to examine aspects of the rDNA such as copy number [6,37,38] and rRNA sequence [7,39] variation. However, the lack of tools to genetically manipulate the highly repetitive rDNA in mammalian systems means that the human rDNA is still not well characterized at the molecular level.

The rDNA IGS has been shown to house a number of functional elements that mediate rRNA regulatory and rDNA extra-coding functions in *Saccharomyces cerevisiae* [11,40–53]. In stark contrast, even though the human IGS is approximately ten times longer than yeast, few functional elements have been defined to date. Those that have are restricted to the rRNA promoter [54], 10 bp repeats (Sal boxes) some of which act as terminators of the primary rRNA transcript [55], and two noncoding IGS transcripts that are associated with stress response [18]. Other elements have been identified from their sequence composition, including several other repeat elements [1], a cdc27 pseudogene [56], and putative c-Myc and p53 binding sites [57,58]. Pioneering work characterizing the chromatin structure of the human IGS has provided evidence for regions with distinct chromatin states, including states characteristic of transcriptional regulatory activity [59]. Furthermore, there appears to be dynamic regulation of this rDNA chromatin structure [60,61]. However, without further characterization, the functional significance of these human rDNA chromatin states is unclear.

Comparative genomics is a powerful method for the identification of functional elements that are difficult to detect by traditional molecular approaches [62–65]. In particular, phylogenetic footprinting is an effective way to identify potentially functional elements using orthologous sequence data alone. The principle is that mutations in functional elements will be deleterious, therefore changes in the sequences of functional elements are selected against and change at a slower rate than non-functional elements over evolutionary time [66]. Thus, comparison of orthologous sequences from related species results in the functional elements appearing as “phylogenetic footprints”—highly conserved regions in a multiple sequence alignment against a background of non-functional, poorly conserved sequences [66]. Application of this method to the rDNA of *S*. *cerevisiae* successfully identified both known and novel functional elements in the IGS [11,51]. Given how little is known about functional elements in the human IGS and the strong connections between rDNA biology and human pathology, we decided to utilize phylogenetic footprinting to identify potential functional elements in the human rDNA.

Here, we constructed complete rDNA sequences from six primate species for which these sequences were previously unknown. Alignment of these sequences with a human rDNA sequence shows that previously identified functional elements in the human IGS are evident as phylogenetic footprints, and there are a number of other conserved regions not associated with any known functional element. Building on the results characterizing the chromatin state of the human rDNA [59], we shed light on the potential functions of these uncharacterized IGS conserved regions by overlaying publicly available RNA-seq, CAGE, and ChIP-seq data onto the conservation profiles. These analyses suggest that chromatin structure and the production/regulation of noncoding transcripts are major activities associated with sequence conservation in the human IGS. This is reinforced by conservation of IGS transcriptional activity in the apes, implying that these activities may be important for human rDNA function.

## Materials and methods

### Whole genome assemblies to obtain the primate rDNA sequences

Whole genome sequencing (WGS) data for the six primates *viz*. chimpanzee (*Pan troglodytes)*, gorilla (*Gorilla gorilla)*, orangutan (*Pongo abelii)*, gibbon (*Nomascus leucogenys)*, rhesus macaque (*Macaca mulatta)*, and common marmoset (*Callithrix jacchus*) were obtained from the Ensemble database (**S1 Table**). Whole genome assemblies (WGAs) for chimpanzee, gorilla, gibbon, macaque and common marmoset were performed using Arachne ver. r37405, and orangutan using Arachne ver. r37578, on a 64-bit server with six-core an Intel Xeon @ 2.67GHz processor and 512 GB RAM. We used Arachne [67,68; **S1 and S2 Tables**], as it resolved the rDNA unit the best in a comparative study of whole genome assemblers that we performed [69]. Default parameters were used for all assemblies. The steps to construct complete rDNA sequences are given in **S1 File.**

### BAC filters screening and BAC clones

BAC filters and E. *coli* containing the rDNA BAC clones were obtained from Children’s Hospital Oakland Research Institute, USA (CHORI; http://www.chori.org) (**S3 Table**). A 594 bp human 18S rDNA PCR product probe (Genbank U13369 coordinates 4,328–4,922) was made using male human template genomic DNA (Promega), primers HS_18S_rDNA_F (5'-AGCTCGTAGTTGGATCTTGG-3') and HS_18S_rDNA_R (5'- GTGAGGTTTCCCGTGTTGAG -3'), and DIG high prime DNA Labeling Kit II (Roche). To identify rDNA-containing BAC clones, Southern hybridization was used to screen the BAC filters with chemiluminescent detection and CDP-Star (Roche). BAC extraction was performed using overnight LB/chloramphenicol (30 μg/L) *E*. *coli* cultures containing the BAC of interest with the NucleoBond Xtra Maxi Plus (Macherey-Nagel) kit.

### Determination of the primate rDNA size

To determine rDNA unit size, 10 μl of purified BAC DNA was digested with 100U of I-*Ppo*I (Promega) overnight. I-*Ppo*I digested products were run on 1% pulsed field certified agarose (Bio-Rad) in 0.5X TBE gels with a CHEF Mapper XA (Bio-Rad) for 31 hrs using FIGE settings 180 V and 120 V forward and reverse voltages, respectively, and a 0.4 sec to 2 sec linear ramp switch time at 14°C. To aid resolution of bands, 5 kb ladder (Bio-Rad) was mixed with loading dye and water in a 1:1:2 ratio and incubated for 2 hrs at 37°C, 50°C for 15 min, and on ice for 10 min.

### rDNA BAC sequencing and analysis

Indexed libraries were prepared from BAC clones using NucleoBond Xtra Maxi Plus (Macherey-Nagel). NGS was performed using Illumina HiSeq 2000 with 2x100 bp paired end reads and a 250 bp insert size. Low quality (<13) ends of reads were trimmed off and reads <25 bp in length were removed using SolexaQA [70]. Processed reads were mapped to the corresponding WGA rDNA sequence using bowtie (ver. 0.12.8). Consensus sequences were generated using a minimum coverage cutoff of 5 with CLC Genomic workbench and aligned to the corresponding WGA rDNA sequence using the MAFFT server [http://mafft.cbrc.jp/alignment/server; 71] with strategy E-INS-I and scoring matrix 1 PAM. Repeat regions in the rDNA sequences were identified using RepeatMasker (http://www.repeatmasker.org) with “DNA source” set as “human”. Alu elements in the IGS were confirmed using DFAM database (ver. 1.1) [http://dfam.janelia.org; 72], and numbered according to their IGS position (starting closest to the 3’-ETS). Other sequence elements in the IGS were identified using YASS [73] and BLAST [74].

### Multiple sequence alignment and similarity plots

Primate rDNA sequences were aligned to the human rDNA sequence (**S1 Appendix)** to generate multiple sequence alignments (MSA) using MAFFT (ver. 6.935b) [71,75] with strategy E-INS-i (—genafpair), 1 PAM scoring matrix (—kimura 1), and gap penalty zero (—ep 0) (**command:** mafft—genafpair—maxiterate 6—thread 6—cluastalout—kimura 1—ep 0—reorder fasta_input_file > seq.aln). Where required, alignments were adjusted by visual inspection. Columns with gaps in the human rDNA reference sequence were removed before similarity plot construction using Synplot [http://hscl.cimr.cam.ac.uk/syn_plot.html; 76] with a sliding window of 50 and increments of 1 bp. Human rDNA annotations were mapped onto the similarity plot using GFF files.

### Identification of conserved regions

Conserved regions in the MSA were identified using phastCons [77,78] using the phylogeny matrix for 99 vertebrates obtained by ENCODE (http://hgdownload.cse.ucsc.edu/goldenPath/hg38/phastCons100way/hg38.phastCons100way.mod; **S2 Appendix**).

### ORC mapping and peak analysis

Single end reads (1x36 bp) for origin of replication (ORC) ChIP-seq and corresponding Input [79] data were processed and mapped to the modified human genome assembly using bowtie ver. 0.12.8 (parameters: -l 30 -n 3 -a—best—strata -m 1). Mapped reads were sorted and duplicate reads were removed using Picard (**S1 File**). ORC enrichment was determined and noise removed using MACS2 (**S1 File**). MACS2 function bdgpeakcall [p-value cutoff 10^−20^ (-c 20)] was used to identify ORC peaks, and enrichment and peaks were visualized using Integrative Genomics Viewer (IGV) ver. 2.3.

### Transcriptome profiling

We introduced the human rDNA sequence into chr21 of the human genome assembly (hg19) to produce a modified human genome assembly. Long RNA-seq [poly(A+) and poly(A-)] and small RNA-seq data for human cell lines HUVEC, GM12878, H-1hESC, K562, HepG-2, and HeLa-S3 were obtained from the Cold Spring Harbor Laboratories long RNA-seq and short RNA-seq databases, respectively (**S3 Appendix**). The long RNA-seq data were mapped to the modified human genome assembly using STAR aligner (ver. 2.2.0) [80]. Mapped reads were assembled (reads mapping to the rDNA coding region were masked) using Cufflink (ver. 2.2.1) [81]. Details are given in **S1 File.** Small RNA-seq data were mapped to the modified human genome assembly using bowtie. Regions with > = 5 read coverage were extracted using bedtools.

CAGE data for human cell lines HUVEC, GM12878, H9-hESC, K562, HepG-2, and HeLa-S3 were obtained from FANTOM [82; Supplemental data 3], and mapped to the modified repeat masked human genome assembly using bowtie (ver. 0.12.8). Masked assemblies were used to avoid multi-mapping reads from repeat regions, as CAGE reads are single end 35 bp reads with pseudo quality values. Paraclu (ver. 3) was used to identify the tag enrichments. Details are given in **S1 File**.

Paired-end (2x101 bp) total RNA-seq data for heart, kidney, liver, lung, and skeletal muscle of chimpanzee were obtained from the Nonhuman Primate Reference Transcriptome Resource [83], and analyzed as for the human RNA-seq analysis (**S1 File**). Poly(A+) single end data (1x76 bp) from heart, kidney, and liver of orangutan and macaque were obtained from Brawand *et*. *al*. [84], and analyzed as for the human RNA-seq analysis (**S1 File**) except that the STAR aligner parameter “outFilterMismatchNmax” was set to “5” and the Cufflink parameter “library-type” was change to “fr-unstranded”.

### Chromatin profiling

Data for histone modifications (H3K4Me1, H3K4Me2, H3K4Me3, H3K9Ac, H3K27Ac, H2.AZ, H3K36Me3, H3K9Me1, H4K20Me1, H3K79Me2, H3K27Me3 and H3K9Me3), RNA polymerases (Pol II and Pol III), transcription factors (TBP, ZNF143, c-Myc, Brf3, Brf1, Brd1, and UBF), CTCF, and Input for cell types HUVEC, GM12878, H-1hESC, K562, HepG-2, HeLa-S3, and A549 were downloaded from ENCODE [85; S3 Appendix]. Reads were processed and mapped to the modified human genome assembly using bowtie (ver. 0.12.8). Enrichment peaks were called using macs2 [86]. Details are given in **S1 File**. Mapped chromatin markers were combined to predict the rDNA chromatin states using Segway, for which a 10-state model underwent unsupervised training on 1% of the human genome [87] before prediction of chromatin states.

### Availability of data and material

The human rDNA sequence from BAC clone GL000220.1 is available (**S1 Appendix**). Primate rDNA sequences constructed using WGS data and sequencing of BAC clones are available from Genbank (accessions KX061886-KX061891 and KX061874-KX061885, respectively, and **S1 Appendix**). Raw NGS data for primate rDNA BACs are available from the Sequence Read Archive (accession SRP068821). The phastCons profile (**S2 Appendix**) and multiple sequence alignment of primate rDNA sequences (**S6 Appendix)** are available. IGV sessions for visualizing the rDNA ChIP-seq peaks, RNA-seq predicted transcripts and CAGE peaks for cell types included in this study are available through figshare **(https://doi.org/10.17608/k6.auckland.6159395.v1)**.

## Results

### Selection of species for phylogenetic footprinting

**Fig 2:**
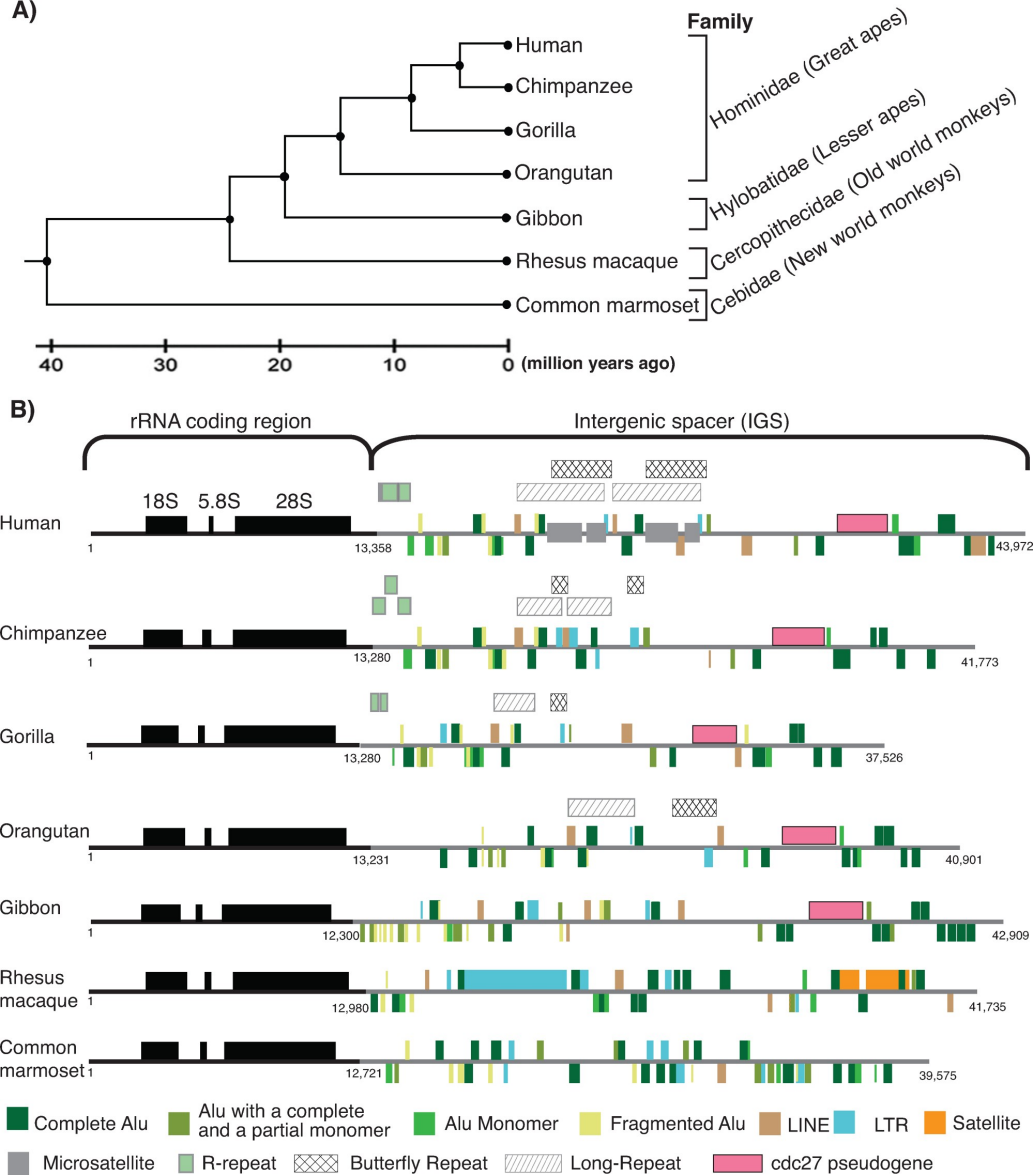
Primate rDNA repeat units. **A)** Phylogenetic tree showing the relationships between primate species selected for rDNA phylogenetic footprinting [adapted from 91]. **B)** Human and primate rDNA unit structures are shown. The rRNA coding region (black line), including the 18S, 5.8S and 28S rRNA subunits (black boxes), and the IGS (grey line) are indicated along with the positions of repeat elements and a cdc27 pseudogene. Elements above the line are on the forward strand; those below on the reverse strand. The rRNA coding region/IGS coordinates and rDNA unit lengths are indicated.

We set out to use phylogenetic footprinting to identify regions in the human IGS that are potential functional but have escaped detection because of the difficulties of working with the highly repetitive rDNA region. To do this, we decided to compare the human rDNA sequence with rDNA sequences from various primates. However, despite the genomes of several primate species having been sequenced, the complete rDNA sequence has not been identified, therefore we constructed rDNA sequences for selected primate species using whole genome assemblies (WGA). We used two criteria to select the primate species for analysis. First was the availability of Sanger whole genome sequence (WGS) data, as preliminary analysis suggested that short-read next generation sequencing data are refractory to the assembly of complete rDNA units. The range of species relatedness is critical for phylogenetic footprinting [88], therefore our second criterion was inclusion of species with varying relatedness to human. Based on these criteria, we selected six primates (of the roughly 300 living species of primates distributed among 13 families [89]) that had Sanger whole genome sequence data available [90]: *Pan troglodytes* (chimpanzee), *Gorilla gorilla* (gorilla), and *Pongo abelii* (orangutan) from the Hominidae, *Nomascus leucogenys* (gibbon) from the Hylobatidae, *Macaca mulatta* (rhesus macaque) from the old world monkeys, and *Callithrix jacchus* (common marmoset) from the new world monkeys. These primates include both species closely related to human (Hominidae and Hylobatidae), together with more distantly related species (old and new world monkeys) (**Fig 2A**).

### Reference human rDNA sequence

The widely used reference human rDNA unit (Genbank accession U13369) was constructed by assembling several partial sequences obtained by different labs [1]. This sequence is known to contain errors [25,92], hence we wanted to use a human rDNA sequence from a single source that is likely to have fewer errors. We chose the complete human rDNA unit sequence (43,972 bp) present in an unannotated BAC clone (Genbank accession GL000220.1; same as Genbank AL592188; **S1 Appendix**) [36] that is present as an unplaced scaffold in the GRCh38 human reference genome and contains a complete and partial rDNA unit together with a part of the rDNA distal flanking region. We refer to this rDNA sequence as the “human rDNA”, and it includes a 13,357 bp coding region and a 30,615 bp IGS (as determined by comparison to the Genbank human rDNA sequence). [36]. Excluding copy number variation in microsatellite and other repeats in the IGS (**S4 Appendix**), the human rDNA shows 98.1% sequence identity to U13369. This human rDNA sequence has 96.6% sequence identity (**S5 Appendix**) to another recently published human rDNA reference sequence [Genbank accession KY962518.1; 39] also derived from a sequenced BAC clone that includes the rDNA distal flanking region (Genbank accession FP236383). The differences are predominantly differences in microsatellite tract lengths **(S5 Appendix; highlighted in orange**), but our reference has two deletions compared to KY962518 (KY962518.1 coordinates 13923–14720 and 28,378–28,580; **S5 Appendix, highlighted in blue**) that total approximately 1 kb. One is a deletion of one repeat copy from the tandem R-repeat region. Chimp has about half of this extra repeat copy, but none of the other primates do. The other is a deletion of one repeat copy from a set of three tandem repeats located within the longer Long Repeat/Butterfly repeat region, with none of the primate species in this study sharing this extra repeat copy. It remains to be determined whether these are natural copy number polymorphisms or assembly artifacts.

### Constructing primate rDNA sequences

To perform phylogenetic footprinting, we first constructed rDNA sequences for the selected primate species using WGA. The high level of sequence identity between rDNA units within a genome [93–95] leads genome assemblers to construct a single, high-coverage “consensus” rDNA unit sequence from the multiple rDNA repeats. The coverage level will be greater than that of unique regions by a factor of the rDNA copy number (about 200–500 in primates; [96,97]). We therefore performed WGA on publicly available WGS data for the primate species (**S1 and S2 Tables**) and selected high-coverage contigs. These contigs were screened using the human rDNA sequence to identify rDNA-containing contigs, were and merged to produce complete rDNA sequences. From this we obtained rDNA units for the six primate species, ranging in size from 37.5–42.9 kb (**Fig 2B**), and the regions corresponding to the rRNA coding region and IGS were identified by comparison with the human rDNA (**S4 Table**). The human coding region aligns completely (end to end) to all primate rDNA sequences except marmoset, for which the 5’ external transcribed spacer (ETS) is 272 bp shorter than the human 5’ ETS. This may be because the marmoset 5’ ETS is actually shorter than human, or because the WGA failed to properly assemble this region.

Use of the human rDNA to identify rDNA contigs in the primate WGAs makes it possible that regions present in other primates, but not in human, were missed. Furthermore, the presence of repetitive elements in the IGS that are also found in other regions of the genome [98] may have led to WGA errors [99]. To eliminate these possibilities, we first identified rDNA-containing BAC clones for the primate species (except chimpanzee, which has a high level of genomic sequence identity to human) by screening BAC genomic libraries (**S3 Table**). We compared the sizes of the WGA and BAC rDNA units by digesting the BAC clones with I-*Ppo*I, a homing enzyme that cuts only once in the rDNA (in the 28S), separating the fragments using field inversion gel electrophoresis (FIGE), and performing Southern hybridization (**S1 Fig**). The estimated lengths of the BAC (via FIGE) and the WGA rDNA sequences are similar (**S1 Fig and S5 Table**), with the FIGE sizes being consistently ~1 kb larger than the WGA sizes (**S5 Table**). The ~1 kb difference in size between our rDNA reference and the published KY962518 reference could account for this difference if this missing sequence failed to assemble in all our primate rDNA sequences, including our human reference. However, as outlined above, some of these missing sequences are present in chimp, suggesting they can be correctly assembled. Therefore, we favor the interpretation that the FIGE gels slightly overestimate the size, and that the primate rDNA sequences are accurate. To further confirm the integrity of the WGA rDNA sequences, the primate rDNA BAC clones were sequenced, and consensus primate rDNA sequences were obtained by mapping the reads to the corresponding WGA rDNA sequences. On average, the consensus BAC rDNA sequences are >97% identical to the WGA sequences (**S6 Table**). The variation is mainly due to gaps in the rRNA coding regions caused by an absence of reads from these regions in the NGS data. The high level of sequence identity (where reads are present) suggests the WGS rDNA sequences are accurate representations of the true rDNA sequences and, given that regions of the rDNA are not represented in the NGS reads, we used the WGA sequences as the reference rDNA sequences for all non-human primate species.

Next, we characterized these new primate rDNA sequences to determine their structural similarity to the human rDNA (**S1 Appendix**). The length of the coding region in the six primate species is similar to human *i*.*e*. approximately 13 kb, except gibbon that is slightly smaller (**S4 Table**). As expected, as we move from chimpanzee to common marmoset, the pairwise sequence identity with human decreases for the coding region (**S4 Table**). The microsatellite component of the rDNA unit in all six primate species is higher than the genome wide average for each species (**Table 1**), and human has the highest microsatellite content because of two long, unique [TC]_n_ repeat blocks (**Fig 2B**). Alu elements are the most abundant repeat element in the primate IGS (**Table 1**), and a number are orthologous between human, apes and rhesus macaque (**S2 Fig and S7 Table and S4 Appendix**). We found that, consistent with a previous report [56], Aluhuman22, Aluhuman25 and Aluhuman27 are present in chimpanzee, gorilla, orangutan, gibbon, and rhesus macaque, while Aluhuman23 is present in apes but not rhesus macaque. It has also been reported that orthologs of Aluhuman26 and Aluhuman28 are present in rhesus macaque [56], but our results show that while these two Alus are conserved in apes, the Alu elements present in similar regions in rhesus macaque are on the opposite strand. Several repeats of unknown function have been identified in the human rDNA (called Long repeats and Butterfly repeats; [1]). These show varying distributions amongst the primates (**Fig 2B**), suggesting they originated at different points in primate evolution. The pseudogene of cdc27 in the human IGS is also present in apes but not in monkeys, as previously reported [56], and the rhesus macaque rDNA sequence contains large LTR retrotransposons and satellite repeats that are absent from the other species (**Fig 2B**). Overall, these results show that a clear signal of orthology and synteny is retained in the rDNA sequences of the selected primates, but there is also sufficient diversity for phylogenetic footprinting to be effective.

**Table 1:** Repeat composition of the primate rDNA sequences as a percent of total rDNA length (with genome-wide percent abundance in parentheses for comparison).

Repeat Elements	Human	Chimpanzee	Gorilla	Orangutan	Gibbon	Macaque	Common Marmoset
**Microsatellites**	20.3^a^ (0.8)	8.7 (0.8)	6.6 (1.1)	7.7 (0.8)	7.7 (0.8)	6.2 (0.8)	10.4(0.9)
**Alus (SINE)**	13.3 (10.6)	13.1 (10.3)	13.3 (8.3)	13.6 (9.8)	16.0 (10.6)	14.2 (10.1)	18.2 (11.0)
**LINE**	4.3 (20.4)	1.6 (21.6)	1.3 (19.8)	1.1 (22.2)	1.6 (21.8)	1.5 (19.1)	0.4 (21.8)
**LTR**	1.2 (8.3)	0.7 (9.0)	0.4 (8.4)	0.9 (9.0)	1.7 (8.7)	12.2 (8.4)	3.60 (1.0)

^a^ 9.34% if the 2 kb [TCTC]n microsatellite at 21,894–23,859 in Fig 2 is removed.

### Conserved regions in the human IGS identified by phylogenetic footprinting

**Fig 3:**
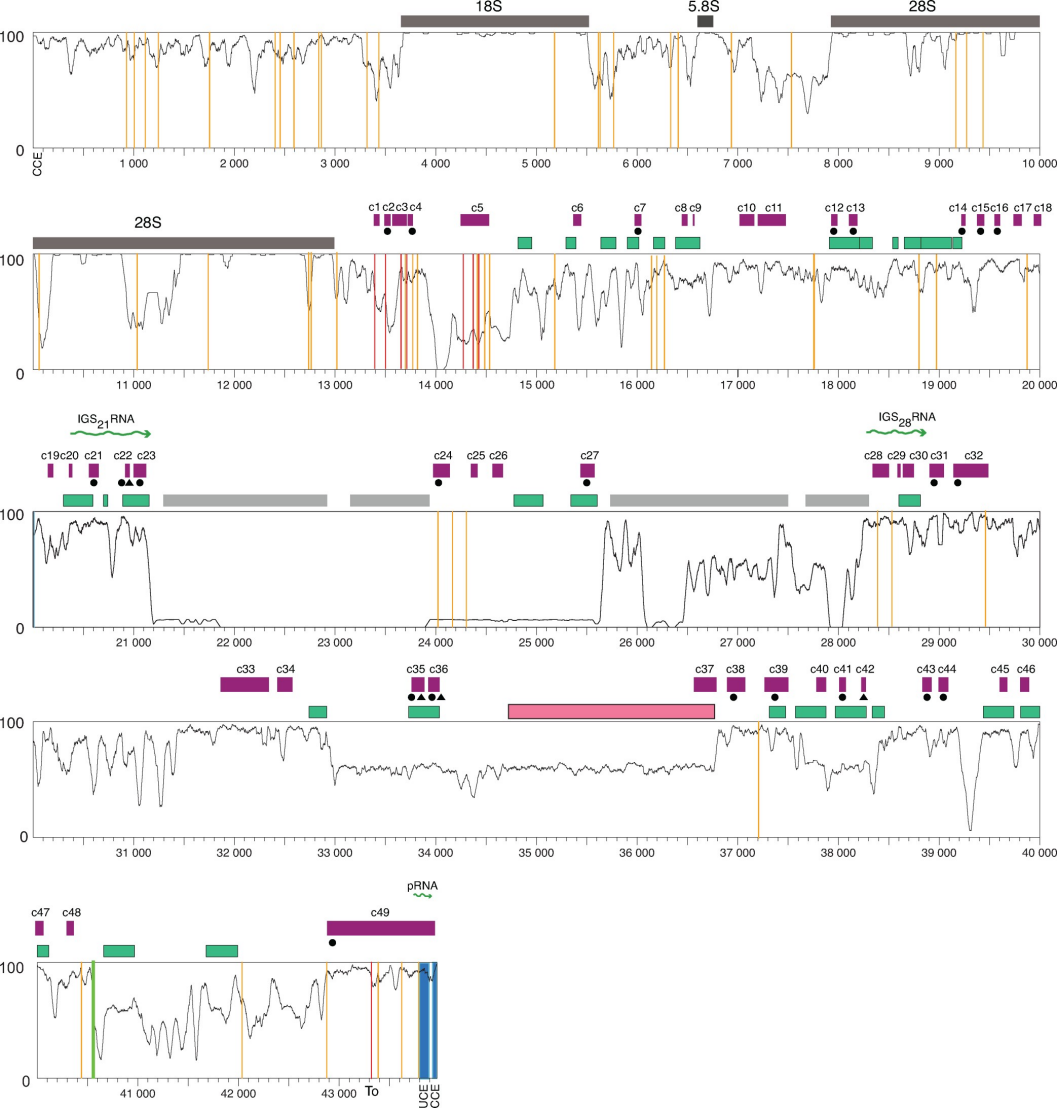
Sequence similarity plot of the primate rDNA. The horizontal axis represents the position in the human rDNA; the vertical axis the level of sequence similarity between 0 (no identity) and 1 (all bases the same). A 50 bp sliding window with 1 bp increment was used to generate the similarity plot. Conserved regions in the IGS (purple boxes) were identified using phastCons. The positions of Alu elements (green boxes), microsatellites (grey boxes), a cdc27 pseudogene (pink box), the rRNA promoter (blue lines), previously identified IGS noncoding transcripts (green wiggly lines), c-Myc binding sites (orange lines), p53 binding site (green line), and Sal boxes (terminator elements; red lines) are indicated. Conserved regions with a black circle or triangle below are conserved in common marmoset and mouse rDNA, respectively.

To identify novel conserved regions that are potentially functional in the human rDNA through phylogenetic footprinting, we aligned the human and primate rDNA sequences. Although the human and common marmoset rDNA sequences align, the alignment is compromised by the relatively low level of sequence identity (**S4 Table**). Therefore, an alignment with the common marmoset omitted (MSA_human-macaque_) was used for the phylogenetic footprinting. The MSA_human-macaque_ has long runs of gaps that are predominantly the result of satellite blocks in the rhesus macaque rDNA (**S6 Appendix**). Because the goal was to identify conserved regions in the human rDNA, all columns in the multiple sequence alignment (MSA) with gaps in the human rDNA were removed. To observe the level of sequence conservation, a similarity plot was generated using Synplot (**Fig 3**). We then identified the regions that are conserved using phastCons, which employs maximum likelihood to fit a phylogenetic hidden Markov model to the alignment [77]. Forty-nine conserved regions (c-1 to c-49) were identified in the human IGS (**Fig 3 and S8 Table**), corresponding to 21.9% of its length. These conserved regions map to both unique regions and Alu elements in the rDNA **(Fig 3)**. We looked to see if these regions are also conserved in the common marmoset and mouse rDNA (using Genbank rDNA reference accession BK000964.3). Twenty-three conserved regions mapped to the common marmoset rDNA, and four mapped to the mouse rDNA, with three found in both, using a >50% identity threshold (**Fig 3 and S9 Table**). Interestingly, two of the three regions conserved with both mouse and common marmoset (c35-36) cover a single Alu repeat (Alu_human_20) with no described function. Together, this phylogenetic footprinting approach reveals conserved regions in the human IGS, including some deeply conserved regions, that represent potentially functional elements.

### Conservation of previously known features in the human IGS

To verify that the phylogenetic footprinting is capable of identifying functional elements in the human rDNA, we looked at whether known human rDNA elements are conserved amongst the primates. As anticipated, the 18S and 5.8S rRNA coding regions are highly conserved across the primates, while the 28S rRNA coding region consists of conserved blocks interspersed with variable regions, as previously reported (**Fig 3**) [100; Fig 3,^101^,^102^]. The rRNA promoter has two characterized elements: an upstream control element (UCE) from position -156 to -107 and a core control element (CCE) from position -45 to +18 [54], and both elements are conserved (**Figs 3 and S3A)**. Several potential rRNA transcriptional terminators (Sal boxes) are present downstream of the 28S rRNA coding region [55,103], and all are conserved (**S3B Fig)**. In addition, the Sal box proximal to the rRNA promoter [55] is conserved, although the functional significance of a terminator in this position is not clear. The c-Myc binding sites identified around the rRNA promoter fall in a conserved region (c49; **Fig 3**), with this area having been shown to bind c-Myc [57]. Several other predicted c-Myc binding sites in the IGS also fall into conserved regions, although the majority (including sites near the terminator that were shown to bind c-Myc) do not (**Fig 3)** [57]. However, conservation of the actual binding motif itself does not automatically translate to a conserved region because of the thresholds used to define conserved blocks (**S4 Fig)**, and some c-Myc binding motifs around the terminator that are not in a conserved region are, nevertheless, conserved. The region corresponding to the pRNA, a noncoding RNA transcript that plays a role in rDNA silencing in mouse [104], coincides with conserved region c49, although it is not conserved with mouse (**Fig 3**). Two human IGS transcripts that are produced as a result of stress [called IGS_21_RNA and IGS_28_RNA; 18]) overlap conserved regions c20-c23 and c28-c30, respectively (**Fig 3**). The conservation of these noncoding IGS transcripts suggests that their function in stress response may be conserved in primates. Together, our results show that a number of elements in the rDNA that are known or have been suggested to be functional appear as conserved peaks, suggesting that our phylogenetic footprinting approach has the ability to identify functional elements in the IGS.

### Association of unknown conserved regions with transcription

Previously known functional elements account for 11 (c1-c3, c20-c23, c28-c30 and c49) of the identified 49 conserved regions. The remaining conserved regions remain uncharacterized, and these regions may represent novel functional elements. Therefore, we next looked for potential functions of these novel conserved regions. The presence of characterized noncoding transcripts in the human IGS [18,104,105], as well as their prominence in the rDNA of other organisms [11,106–108], led us to explore whether some of the conserved regions are associated with noncoding transcription. We mapped publicly available long poly(A+) and poly(A-) (>200bp), and small RNA (< 200 bp) RNA-seq data [109] from all six cell lines of the first two tiers of the ENCODE project to a modified human genome assembly to which we added the human rDNA sequence (“modified human genome assembly”), without repeats masked. The cell lines included two normal cell lines (HUVEC and GM12878), one embryonic stem cell line (H1-hESC), and three cancer cell lines (K562, HeLa-S3, and HepG-2). Several novel poly(A+) and poly(A-) transcripts were identified, including transcripts in common across all cell lines, and transcripts restricted to a subset of cell lines (**S5 Fig and S10–S21 Tables**). To identify potential transcriptional start sites (TSS) for these noncoding transcripts, we mapped publicly available CAGE data from the FANTOM5 project [82] to the modified human genome assembly with repeats masked (to prevent spurious alignment of the short CAGE sequence reads). Several CAGE peaks were identified that support the presence of some of the novel IGS transcripts (**S5 Fig and S22 Table; Bed files for RNA-seq transcripts and BedGraph files for CAGE peaks are available at figshare location https://doi.org/10.17608/k6.auckland.6159395.v1**).

**Fig 4:**
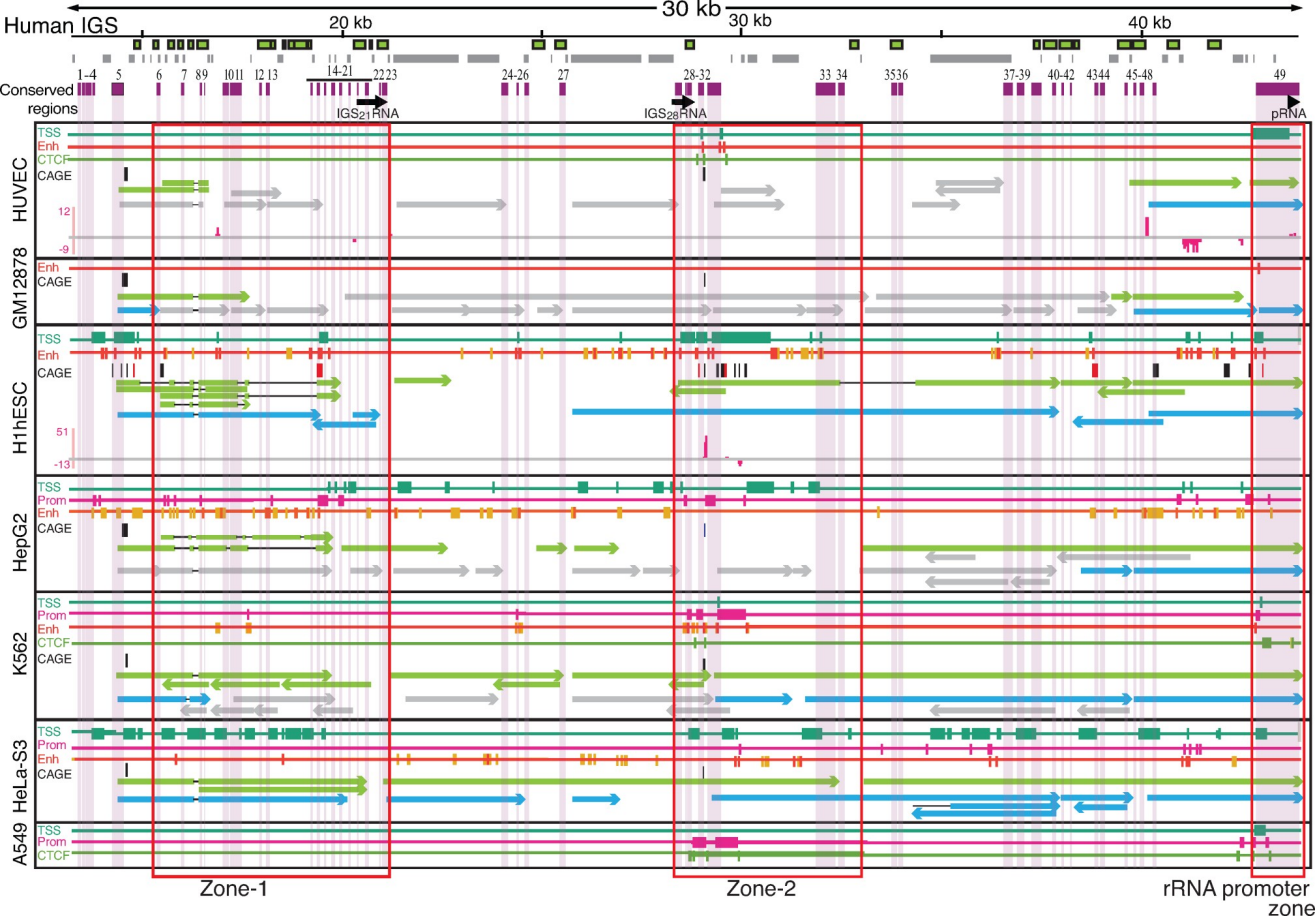
Two zones in the human IGS enriched for conserved regions and transcription associated factors. The human IGS is shown at top, with the positions of Alu elements (green boxes), microsatellites (grey boxes), conserved regions (purple boxes), and previously identified IGS noncoding transcripts (black arrows) indicated. Below are chromatin and transcriptional features of seven human cell lines. The positions of the conserved regions are indicated by pale shading. For each cell line the presence of transcriptional start site (TSS), promoter (Prom), enhancer (Enh), and CTCF segmentation states, obtained by merging peaks for histone modification, Pol II and CTCF using Segway, are indicated. Below these, CAGE peaks are shown for the forward (black boxes) and reverse (red boxes) strands (CAGE stem cell data come from H9-hESC, not H1-hESC), followed by long poly(A+) and poly(A-) transcripts (green and blue arrows, respectively) with FPKM values >1; gray arrows indicate transcripts with FPKM < 1. Arrowheads indicate the direction of transcription. Peaks of small RNA are shown in pink. Zones 1 and 2 that are enriched for conserved regions and transcription-associated factors are boxed in red. Not all features have data available for all cell lines.

The presence of transcripts that originate from the human IGS implies that transcriptional regulators (e.g. promoters, enhancers and insulators) are present in the IGS, and may correspond to some of the conserved regions. Therefore, we mapped publicly available ENCODE ChIP-seq data for histone modifications, RNA polymerase-II and III, transcription factors (TBP, c-Myc and ZNF143), and the insulator binding protein CTCF, a highly conserved protein that is involved in the three-dimensional organization of chromatin [110–112], to the modified human genome assembly. We used ChIP-seq data from the six cell lines that were subjected to RNA-seq analysis, as well as from an additional cancer cell line (A549) from tier-3 of the ENCODE project. Several peaks of enrichment for these factors were identified (**S6–S12 Figs; BedGraph files for ChIP-seq peaks are available at figshare location https://doi.org/10.17608/k6.auckland.6159395.v1)**, with those associated with active transcription being distinct and sharp, while those associated with transcriptional repression are comparatively broad, as previously observed [59]. Cell line HeLa-S3 is an exception as the histone modifications peaks associated with active transcription are broad as well. The GM12878 cell line has fewer prominent histone modification peaks than the other cell lines, probably because of loss of a substantial number of ChIP-seq reads during the quality control step for this cell line. We then integrated the histone modification, CTCF, and Pol-II profiles for all seven cell lines using Segway [113] to determine putative chromatin states in the IGS (**S13 Fig and S23 Table**). Finally, we intersected the RNA-seq, CAGE, and chromatin state datasets with the conserved regions to identify transcripts and chromatin states that are potentially functionally conserved. This analysis revealed three prominent zones in the IGS containing several conserved regions that either show evidence for active transcription or have chromatin states associated with transcription (**Fig 4**). Together, these zones account for 18 of the 38 unknown conserved regions, including 14 of the 23 regions conserved with the common marmoset. The first zone is located near the rRNA transcriptional terminator, and we call this zone-1. It encompasses conserved regions c6 to c23 (~14.8 kb—21.1 kb) (**Fig 4**) and contains a number of both poly(A+) and poly(A-) transcripts common to all cell lines (**S5 Fig)**, many of which appear to be spliced. There are a number of peaks of histone modifications that indicate chromatin states associated with transcription, most prominently in the H1-hESC and HepG2 cell lines. A number of the putative transcripts appear to originate upstream of this zone, in a region that is enriched for chromatin states associated with active transcription and with CAGE peaks but does not show sequence conservation. Zone-1 also contains the previously identified IGS_21_RNA noncoding transcript (**Fig 3**).

The second zone is roughly in the middle of the IGS, and we call this zone-2. It encompasses conserved regions c28-c34 (~28.2 to 32.6 kb; **Fig 4**) and shows strong enrichment for chromatin states associated with transcription and transcriptional regulation. Conserved regions c28-c30 correspond to the previously identified IGS_28_RNA noncoding transcript [18,59], and, consistent with previous results [59], show chromatin states associated with transcriptional activity (**Fig 4**). While we do not detect IGS_28_RNA specifically, we do find transcripts that overlap it. Conserved regions c31-c32 show an enrichment of active chromatin states, as reported previously [59], as well as transcripts in many cell lines (**Figs 4 and S5**). This region also shows a peak of CAGE tags in the same position in all cell lines for which CAGE data are available (**Figs 4 and S5**). Interestingly, there are two oppositely transcribed small RNA peaks in conserved region c31 that may represent transcription from a bidirectional promoter and are only observed in H1-hESC (**Figs 4 and S5**). In general, more CAGE tag peaks map in the stem cell line than the other cell lines, mirroring genome-wide patterns of embryonic stem cell expression [114] and suggesting the rDNA might be in an unusually permissive chromatin state for noncoding transcription in this cell type. Furthermore, zone-2 was the only part of the IGS for which CTCF segmentation states were predicted in all cell lines that had data.

The final zone encompasses the rRNA promoter (**Fig 4**). Noncoding transcripts are found in this zone (**S6–S11 Figs**), including small RNA peaks in the HUVEC cell line. Some of these transcripts may function like the mouse pRNA, a small RNA that influences rRNA transcription [104], with pRNA-like transcripts having been detected in the human rDNA before [59]. This zone also displays chromatin features characteristic of TSSs, promoters, and enhancers, depending on the cell line (**Fig 4**), and again, some of these features might relate to the presence of the pRNA. However, whether humans have a pRNA that is functionally equivalent to the mouse pRNA has not yet been determined.

Our analyses also show a number of poly(A+) and poly(A-) transcripts, small RNAs, and chromatin states associated with transcriptional activity outside of these zones. In some cases these overlap with conserved regions, but in other cases they do not, and it is difficult to determine whether the transcriptional features that overlap conserved regions are associated with the conservation or not. A number of the nonconserved transcriptional features correspond to microsatellite regions (**S12 Fig**), suggesting they might be artifacts of the spurious alignment of reads to IGS microsatellites [99]. However, microsatellites have been shown to act as promoters and/or enhancers [115–119], hence we cannot completely rule out that the chromatin states at these sites are real.

### Replication and double strand break association

**Fig 5:**
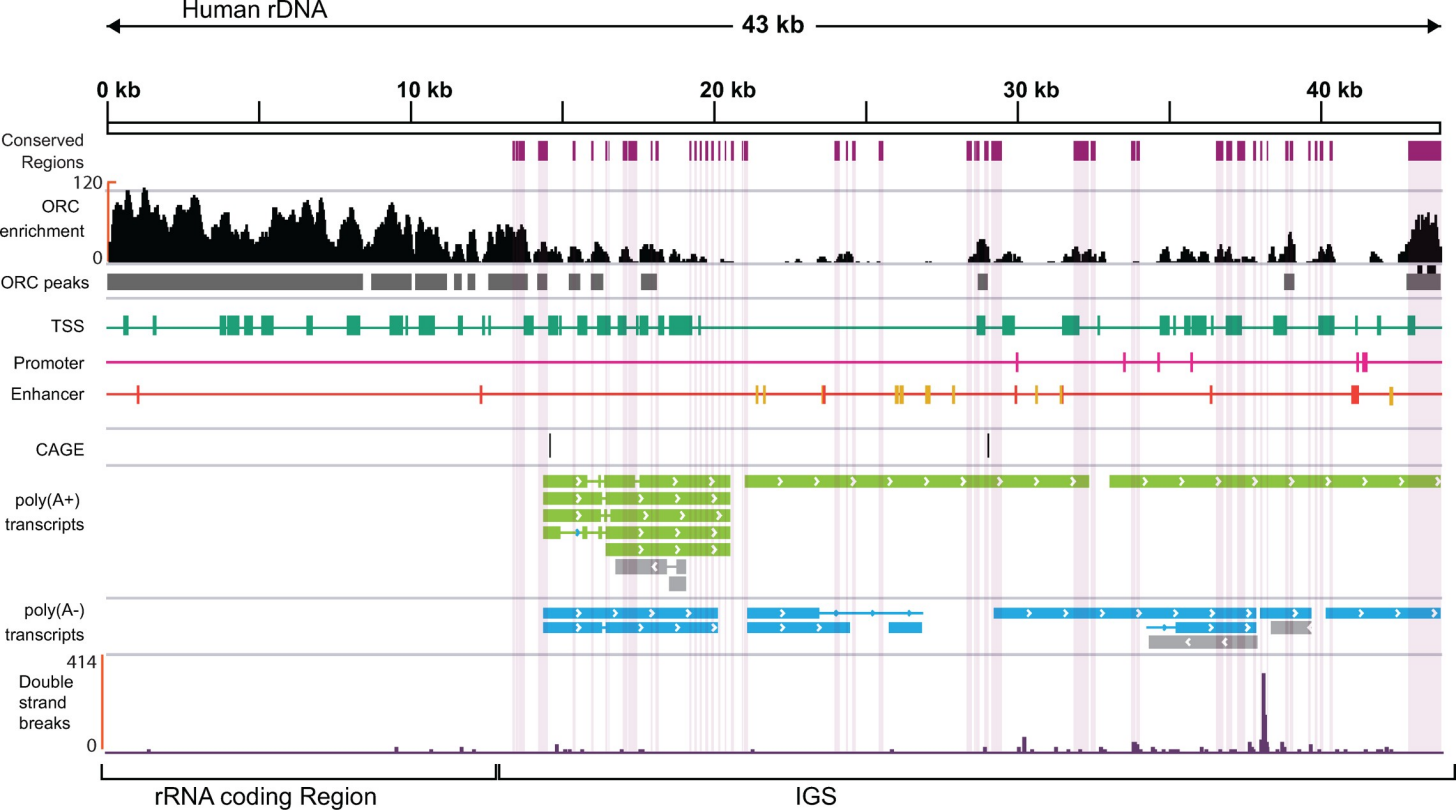
Origin replication complex (ORC) and double strand break (DSB) occurrence in the rDNA. The black plot represents enrichment of ORC in Hela-S3 cells and grey boxes below represent the position of peaks. Scale on the left is the -fold enrichment, and the scale above shows the position in the rDNA. Purple boxes represent conserved regions. The predicted chromatin states: transcription start site (TSS; green boxes), promoter (pink boxes), and enhancer (orange boxes) are shown. CAGE peaks are shown as black boxes (positive strand). Long poly(A+) and poly(A-) transcripts with FPKM values > 1 are shown as green and blue boxes, respectively. Gray arrows show transcripts with FPKM < 1. Arrows indicate the direction of transcription. The purple plot at bottom represents the DSB sites in HEK293T cells.

The presence of origin of replication activity is a conserved feature of the rDNA [46,120–124]. Genome-wide mammalian origins of replication are not defined by sequence and there is not agreement on precisely where replication initiates in the rDNA [122,125–128]. We looked to see whether origin of replication complex association overlaps with conserved regions in case the rDNA initiates replication in a sequence-specific manner. We mapped publicly available origin of replication complex (ORC) ChIP-seq data [79] to the modified human genome assembly. The majority of ORC signal in the rDNA is found distributed across the rRNA coding region and the regions immediately flanking this (**Fig 5**). However, six smaller peaks of ORC enrichment are seen in the IGS, with five of them falling in conserved regions **(Fig 5)**. These results suggest that the majority of replication in the human rDNA initiates in the rRNA coding region and/or the regions flanking it, consistent with reports that mammalian origins of replication are enriched in transcriptionally active regions [79]. Whether there is any biological significance to the minor ORC peaks at the conserved regions in the IGS is unclear.

A key feature of the rDNA repeats in yeast is the presence of double strand breaks (DSB) at a conserved site of unidirectional replication fork stalling known as the replication fork barrier site [49,50,129]. We examined whether recently reported DSB sites in the human rDNA [130] are located around conserved regions, but found no consistent pattern of association (**Fig 5**). Interestingly, however, the major DSB site in the rDNA lies in a region that is close to one peak of ORC enrichment, potentially suggesting the DSB site is a region of replication restart, such as observed at the yeast rDNA [131]. However, this site is at the opposite end of the IGS to where human replication fork barrier activity has been reported [132].

### Long noncoding RNAs are conserved among primates

**Fig 6:**
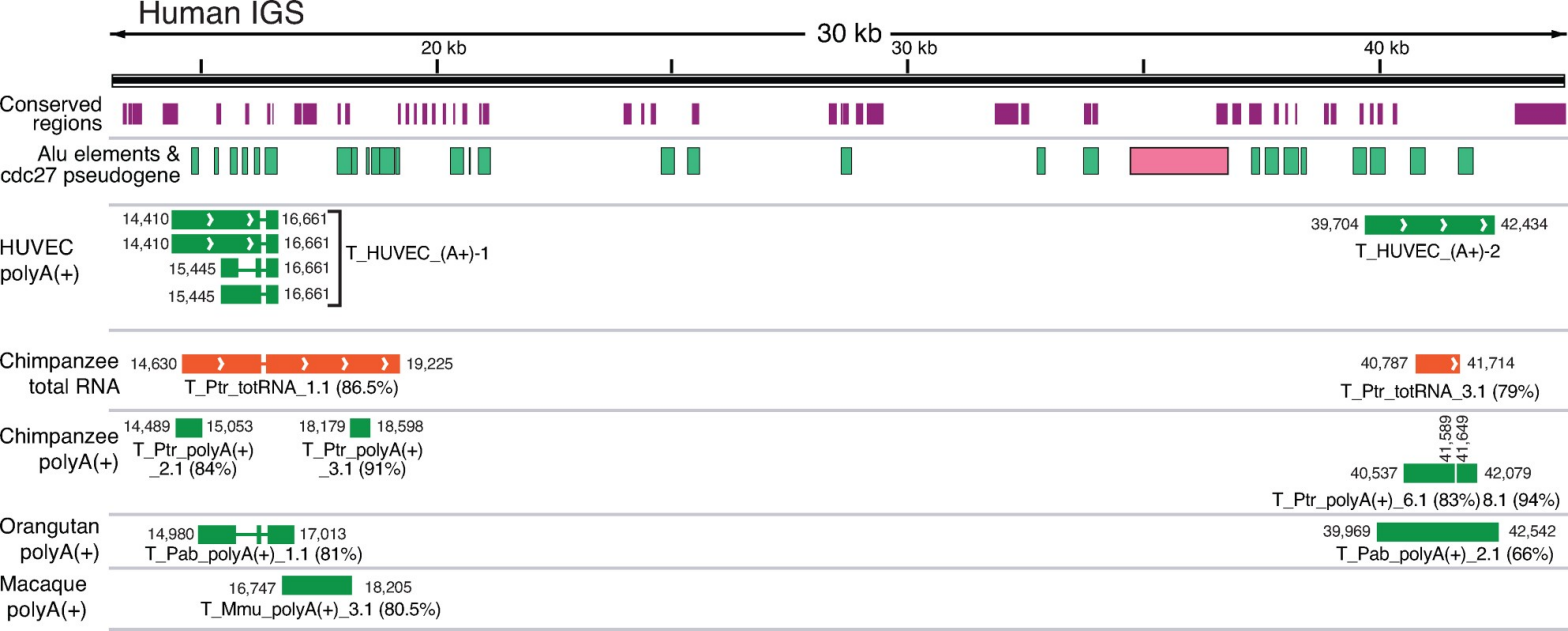
Conservation of human IGS transcripts amongst primates. The human IGS is indicated at top along with the conserved regions (purple boxes), Alu elements (green boxes) and cdc27 pseudogene (pink box). Below are poly(A+) IGS transcripts from the HUVEC cell line, followed by total RNA chimpanzee IGS transcripts (orange), and poly(A+) IGS transcripts from chimpanzee, orangutan, and rhesus macaque (green boxes). Only transcripts that are in common with human are shown. Transcript names and their start/end coordinates are indicated alongside, as are percent identities between each transcript and the human IGS (in parentheses). Arrowheads indicate direction of transcription.

Finally, we reasoned that the presence of transcripts and chromatin states associated with active transcription in conserved regions of the human IGS suggests that similar transcripts should be present in other primates. To test this, we took publicly available paired end total RNA-seq data from liver, lung, and skeletal muscle of chimpanzee [83], and single end poly(A+) RNA-seq data from liver, heart, and cerebellum of chimpanzee, orangutan, and macaque [84]. These data were mapped to the corresponding species’ genome assembly to which the appropriate rDNA sequence had been inserted. We found IGS transcripts in all tissues from chimpanzee and orangutan **(S14–S16 Figs and S24–S27 Tables**), but in macaque such transcripts were only present in liver and heart tissue. We compared the primate IGS transcripts to HUVEC IGS transcripts, as HUVEC is a primary cell line that has a normal karyotype and is not artificially immortalized, hence is likely to be the closest to a “normal” human cell state. Transcripts similar to those found around the human promoter region are also found in chimpanzee and orangutan. In addition, transcripts similar to those found in zone-1 in the human IGS are found in all primate species we analyzed (**Fig 6**). Strikingly, there is conservation of splice junctions between human, chimpanzee and orangutan, even though the full lengths of the transcripts are not the same. No transcripts corresponding to zone-2 were found for the non-human primates analyzed here, and only one IGS transcript was found in macaque in zone-1, although this transcript does not overlap the HUVEC transcripts. Therefore, some but not all of the IGS transcripts that emanate from conserved regions in human are conserved across the apes, supporting the idea that these regions may have been conserved to maintain this transcriptional function. However, the lack of IGS transcripts in macaque suggests that transcriptional conservation does not extend as far as the monkeys, although we cannot rule out that the appropriate macaque tissues have not been sampled to find these IGS transcripts, or that their absence simply reflects a loss that is unique to macaque. The lack of transcripts from zone-2 in apes suggests that enrichment of transcriptional regulatory features in conserved regions in this zone may be involved with determining a specific chromatin structure, or that the production of transcripts is tissue-specific, such as the potentially stem cell-specific bidirectional RNA we identified in this region.

## Discussion

In this study, we combined phylogenetic footprinting, a powerful tool to identify novel functional regions that are conserved over evolutionary time, with genomic datasets to overcome the challenges posed by the highly repetitive nature of the rDNA. In total, we identified 49 conserved regions in the human rDNA IGS. Several of these regions correspond to known functional elements, including the rRNA promoter and terminators, IGS noncoding transcripts, and protein binding sites, while others are novel. The novel conserved regions are dispersed throughout the IGS and correspond to both unique regions and repeat elements. The conserved regions identified here are restricted to elements that share a potential function with most of the primate species examined, and therefore do not include functional IGS elements that have evolved more recently in the lineage leading to humans. However, it may be possible to detect potential human-specific elements via determination of human accelerated regions [133]. Nevertheless, our results catalogue a large suite of potentially functional, uncharacterized regions in the human rDNA that will allow targeted investigations of their functionality. Our work has also provided complete rDNA reference sequences for six primate species that were previously unavailable. These new sequences will facilitate a better understanding of the rDNA in these primates and offer a strong comparative base for additional studies on the human rDNA.

Following the IGS chromatin state characterizations made by Scacheri and colleagues [59], we used several publicly available sequence databases to determine whether the conserved regions show distinctive chromatin states and/or noncoding transcripts that could provide evidence for the functions they putatively play. We found numerous long poly(A+) and poly(A-) transcripts in the human IGS, including many that have not been previously reported, suggesting there is pervasive transcription of the human IGS that is consistent with pervasive transcription in other regions of the genome [109,134,135]. Long noncoding RNAs from the IGS have been reported to be involved in regulating rRNA transcription [104] and stress response [18], therefore some of the novel long IGS transcripts we identified here may also be functional, and a number are conserved in part or whole. However, as for much of the genome-wide pervasive transcription, further work is required to determine what functions, if any, the novel IGS transcripts we document here have.

Mapping of chromatin datasets to the rDNA revealed several regions with chromatin structures that are consistent with transcriptional activity in the IGS, and with those previously reported [59]. Importantly, many of these putatively regulatory regions overlap conserved regions. In particular, two zones show a preponderance of conserved regions and features associated with transcription. Long poly(A+) transcription from zone-1 and near the promoter region is consistently observed in all the cell lines we examined, and some, but not all, of this transcriptional activity is reinforced by chromatin marks associated with active transcription. The presence of transcripts emanating upstream of zone-1 in all cell lines is striking (**Figs 4 and S5**), although the exonic structure of these transcripts is variable and their expression in different cell lines is also variable (**S17 Fig**). While these could represent read-through rRNA transcription, there are three reasons to suggest they do not. First, they are present as both polyA+ and polyA- transcripts, whereas if they were read-through rRNA transcripts, polyA- signals would be expected to predominate. Second, there is no reason to expect read-through rRNA transcripts to be spliced. Third, they appear to originate downstream of coding region, whereas read-through transcripts should be contiguous with the coding region. Indeed, all cell lines show a peak of CAGE tags in the general vicinity of the start of these transcripts. Neither the start of the transcripts nor the CAGE tag peaks fall in conserved regions, suggesting that either these transcripts are not conserved, the transcriptional start site does not need to be conserved at the sequence level, the conserved elements are too small to pass our threshold for a conserved block, or the conserved regulatory elements are located upstream or downstream of the TSS. The presence of transcripts, including some with the same splice junctions, in zone-1 in apes is further evidence that transcription in these regions may have functional significance. In contrast, zone-2 consistently shows chromatin marks associated with transcriptional activity across the cell lines we examined, but less consistent signals of actual transcripts. In addition, zone-2 lacks conserved IGS transcripts in any primate species we surveyed, suggesting that the conserved regions may not be associated with transcription. The pattern of conserved regions and open chromatin features in this zone suggest the conserved regions may have a function not associated with transcription. We suggest that enrichment of marks associated with active chromatin may be the result of these regions maintaining chromatin states that are important for rDNA function. Overall, given that the majority of IGS conserved regions fall into these zones and that the presence of active chromatin states has been documented in these regions previously [59,136], testing these zones for function is a high priority.

A major limitation of this and other studies looking at the rDNA is that the transcription and chromatin mapping results only give an average picture across all rDNA repeats, as mapping of sequence reads cannot currently distinguish individual rDNA repeats. Therefore, it is not possible to categorically associate factors such as chromatin marks of active transcription with transcripts, as the signals may come from physically distinct repeats. For example, there is evidence that some rDNA repeats exist outside of the nucleolus [137], and these may have a different transcriptional or chromatin profile to those located within the nucleolus. Similar limitations exist for trying to determine whether different histone modifications and transcription factors are located in the same rDNA repeats or not. Therefore, the chromatin profiles we observe might be an artificial composite of multiple, distinct states that exist in different rDNA units. Systems that are able to distinguish individual repeat units will be required to resolve these multi-copy issues of the rDNA.

The distinct nature of the embryonic cell line compared to the other cell lines is striking. This is most clearly seen in zone-2, where there are bi-directional small RNA peaks and a number of strong CAGE tag peaks that are specific to the stem cell line. Bi-directional small RNAs can act as enhancer RNAs [138,139], therefore it is possible that the bi-directional small RNA identified here is acting as a development-specific enhancer in embryonic stem cells [140]. rRNA transcriptional enhancers have been reported from *Xenopus*, *Drosophila*, mouse, and rat [141–145], but not human to date. Therefore, if this bi-directional small RNA is acting as an enhancer, it may be enhancing rRNA transcriptional activity. Evidence suggests that rRNA transcription is elevated in embryonic cell lines and is downregulated to initiate differentiation [31–33,146]. Moreover, rRNA expression has been reported to be higher in certain embryonic cell lines than cancer cell lines [147]. Therefore, it will be interesting to determine whether this bi-directional small RNA plays any role in rRNA transcriptional regulation and pluripotency.

The rDNA units are arranged in loops inside the nucleolus [148], and this is facilitated by c-Myc [149]. This loop arrangement results from interactions between regions close to the rRNA promoter and terminators that are enriched for c-Myc [150], and interestingly these correspond to the promoter and zone-1, respectively. Recently, it has been shown that looping of rDNA units is also promoted by other regions of the IGS that interact with nucleolar matrix [151]. These regions correspond to conserved regions c15-c18, c31-c32, c33-c39, and c49, which also have c-Myc binding sites and many of which are enriched for c-Myc [151]. Interestingly, CTCF segmentation states that overlap c31-32 were predicted in zone-2 by Segway in all cell lines that had data. Based on our results and the roles that CTCF and c-Myc play in rRNA transcriptional regulation and genome organization [152,153], we speculate that some of the conserved regions play a role in mediating the three-dimensional organization of the rDNA repeats in the nucleolus, facilitated by the association of CTCF and c-Myc with these regions [110,111,154].

In summary, our results provide a platform for comprehensively characterizing the functional landscape of the human IGS, and for developing a better understanding of the biological processes occurring in the rDNA and the nucleolus. They provide numerous predictions for functional elements in the IGS, in the form of conserved regions, and integrate a rich compendium of functional data to begin interpretation of the roles of these conserved regions. The strong association between the rDNA and human disease provides the impetus for characterizing functional elements in the IGS to better understand how they contribute to human health and wellbeing, and our results provide the basis from which to focus this functional characterization of the human rDNA.

## Supporting information

S1 Appendix Human rDNA sequence extracted from BAC clone GL000220.1, and the primate rDNA sequences generated in this study. (FASTA)

S2 Appendix PhastCons phylogenetic models for conserved and nonconserved regions used in this study. (ZIP)

S3 Appendix Details of ChIP-seq, RNA-seq and CAGE data used in this study. (XLSX)

S4 Appendix Additional supporting information for this study. (DOCX)

S5 Appendix Sequence alignment between human rDNA reference sequence and KY962518. (PDF)

S6 Appendix Multiple sequence alignment of the human rDNA and other primate rDNA sequences used in this study. (ALN)

S1 File Supporting methods. (DOCX)

S1 Fig Estimating the lengths of rDNA units in primate BAC clones. The rDNA BAC clones for A) Gorilla B) Orangutan C) Gibbon D) Rhesus macaque and E) Common marmoset were used to determine the rDNA unit lengths for these species. For each primate, undigested (U) and I-*Ppo*I digested (D) BACs were run on a FIGE gel (left panels) to determine rDNA unit size. The gels were probed with an 18S rDNA fragment (Southern blots; right panels) to verify the bands contain rDNA. Arrows indicate the rDNA bands. A) Gorilla rDNA BAC bands are ~42 kb. In the digested CH276-103L10 lane, the band above the rDNA band is undigested DNA, as it is the same size as the band in the undigested lane (U). In the digested CH276-120P14 lane, the band above the rDNA band is likely to be *E*. *coli* genomic DNA as it is the same size as the band in the undigested lane (D) and has no corresponding signal in the Southern blot. B) Orangutan rDNA BAC bands are ~42 kb. In the digested CH276-103L10 lane, the band above the rDNA band is undigested DNA, as it is the same size as the band in the undigested lane (U). In the digested CH276-120P14 lane, the band above the rDNA band in the gel is *E*. *coli* genomic DNA as it is the same size as the band in the undigested lane and has no corresponding signal in the Southern blot (D). C) Gibbon rDNA BAC bands are ~44 kb. In the digested lanes, the band above the rDNA band is undigested DNA as it is the same size as the band in the undigested lane (U). The bands below the rDNA band in CH271-470I24 are probably the BAC backbone. D) Rhesus macaque rDNA BAC bands are ~42.5 kb. In the digested CH250-26D15 lane, the two bands above the rDNA band are a complete rDNA unit with a partial unit (lower band) and *E*. *coli* genomic DNA (upper band; same size as the band in the undigested lane and no corresponding signal in the Southern blot). In the digested CH250-46L14 lane, the band above the rDNA band is a complete rDNA unit with a partial unit. In the digested CH259-119I6 lane, the band above the rDNA band is undigested DNA, as it is the same size as the band in the undigested lane (U). In the digested CH250-701 lane, the two bands above the rDNA band are a complete rDNA unit with a partial unit (lower band) and *E*. *coli* genomic DNA (upper band; same size as the band in the undigested lane and no corresponding signal in the Southern blot). E) Common marmoset rDNA BAC bands are ~40 kb. In the digested CH259-137E18 lane, the band above the rDNA band is *E*. *coli* genomic DNA as it is the same size as the band in the undigested lane and has no corresponding signal in the Southern blot (D). In the digested CH259-119I6 lane, the band above the rDNA band is undigested DNA as it is the same size as the band in the undigested lane (U). Numbers on the left are the 5 kb ladder sizes used to estimate rDNA unit size.
(PDF)

S2 Fig Repeat elements in the IGS of different primate species. The IGS is shown as a grey line. Repeat elements are indicated above and below the IGS as follows: Alu elements (green boxes), LTRs (blue boxes), LINEs (brown boxes), and satellites (orange boxes), with the names alongside. The cdc27 pseudogene is shown as a pink box. Elements above the rDNA are on the forward strand; elements below are on the reverse strand. The start and end coordinates of the IGS are indicated.
(PDF)

S3 Fig Sequence conservation of human rRNA transcriptional regulators. A) Alignment of the human rRNA promoter region, which encompasses the upstream control element (UCE; brown box) and core control elements (CCE; green box) as indicated by the numbering below relative to the transcription start site. Bases that match human are in black, mismatches are in grey. B) Alignments of potential rRNA terminators (Sal boxes) in the human IGS. The name of the terminator is indicated on the top of each alignment, and the coordinates relative to the human rDNA sequence are indicated. The nucleotides that match the 11 bp human rRNA terminator consensus sequence (GGGTCGACCAG) (Haltiner et al. 1986) are in black, mismatches are in grey. Absence of a terminator is indicated by hyphens. Alignments corresponding to conserved regions identified in this study are shown in pink boxes, with the name of the corresponding conserved region indicated below. Numbering above the alignments refers to the positions in the human rDNA sequence.
(PDF)

S4 Fig Sequence conservation of potential c-Myc binding sites in the human IGS. Alignments of all 29 potential c-Myc binding sites in the human IGS are shown. Alignments corresponding to conserved regions identified in this study are shown in pink boxes, with the name of the corresponding conserved region indicated below. The coordinates of each c-Myc binding site relative to the human rDNA sequence are indicated on the top of each alignment. The nucleotides that are conserved with human are shown in black and that mismatches are in grey. Absence of an orthologous c-Myc binding site is indicated by hyphens.
(PDF)

S5 Fig The transcriptomic landscape of the human IGS in different cell lines. The human IGS with conserved regions (purple boxes) and Alu elements (green boxes) is shown at the top. The diagonal shaded region shows the position of the cdc27 pseudogene. Each cell line is separated by thick black lines. The black (plus strand) and red (minus strand) boxes represent CAGE tag signals. Long polyA(+) (green boxes) and polyA(-) (blue boxes) transcripts with FPKM value > 0.5 are shown. The arrowheads show the direction of transcription. Grey boxes represent polyA(+)and polyA(-) transcripts (depending on the lane the box is present in) with FPKM value < 1. The small RNA (< 200 bp) signals are shown as pink peaks with the scale (pink bracket on the left) representing the number of reads (negative values represent the reverse strand). The cell line is indicated to the left, although the CAGE stem cell data come from H9-hESC, not H1-hESC.
(PDF)

S6 Fig Chromatin, transcription factor and transcript landscape of the IGS in the umbilical vein endothelial cell line, HUVEC. The scale at the top shows the position in the rDNA unit, and the start of the IGS is indicated by the pink vertical line. Purple boxes with purple shaded regions below represent the conserved regions. The position of cdc27 pseudogene is shown as a diagonally shaded region. Each row represents phastcon signal (pink boxes), the enrichment for active histone modifications (green signals), repressive histone modifications (red signals), CTCF (orange signals), RNA polymerase II (Pol-II; blue signals), CAGE peaks (black boxes), long polyA(+) transcripts (green boxes), and long polyA(-) transcripts (blue boxes). The scales on the left represent the levels of enrichment. Grey rows represent the absence of the data or no signal in the human IGS for the ENCODE dataset.
(PDF)

S7 Fig Chromatin, transcription factor and transcript landscape of the IGS in the lymphoblastoid cell line, GM12878. Figure as for S6 Fig, except that the blue signals represent transcription factors and RNA polymerases, and pink signals indicated small RNA transcripts.
(PDF)

S8 Fig Chromatin, transcription factor and transcript landscape of the IGS in the embryonic stem cell line, H1-hESC. Figure as for S7 Fig. The CAGE data come from H9-hESC, not H1-hESC.
(PDF)

S9 Fig Chromatin, transcription factor and transcript landscape of the IGS in the hepatocellular carcinoma cell line, HepG2. Figure as for S7 Fig.
(PDF)

S10 Fig Chromatin, transcription factor and transcript landscape of the IGS in the leukemia cell line, K562. Figure as for S7 Fig.
(PDF)

S11 Fig Chromatin, transcription factor and transcript landscape of the IGS in the cervical carcinoma cell line, HeLa-S3. Figure as for S7 Fig.
(PDF)

S12 Fig Chromatin, transcription factor and transcript landscape of the IGS in the adenocarcinoma cell line, A549. Figure as for S7 Fig.
(PDF)

S13 Fig Genomic segmentation showing functional annotation states in the human IGS. The segmentation states were obtained by merging histone modification, Pol II and CTCF peaks using Segway. The conserved regions (purple boxes) in the human IGS, also shown as grey shadows through the cell lines, are indicated at the top along with Alu elements (green boxes) and microsatellites (grey boxes). The diagonally shaded region represents the cdc27 pseudogene. Segmentation states for each cell line are boxed below, with the name of the cell line indicated to the left. The predicted states shown are: transcription start sites (TSS; green boxes), promoters (pink boxes), and enhancers (orange boxes). CAGE peaks are shown as black boxes (positive strand) and red boxes (reverse strand). The CAGE stem cell data come from H9-hESC, not H1-hESC. Long poly(A+) and poly(A-) transcripts with FPKM values > 1 are shown as green and blue arrows, respectively. Gray arrows show transcripts with FPKM < 1. The arrows indicate the direction of transcription. Small RNA peaks are shown in pink. Not all features have data available for all cell lines.
(PDF)

S14 Fig Transcripts in the Chimpanzee IGS. Transcriptome assemblies were performed using stranded total (top half; orange colors) and unstranded polyA(+) (bottom half; green colors) RNA-seq data from different tissues of chimpanzee. The first (dark orange boxes) and seventh (dark green boxes) rows represent the consensus transcripts obtained by merging together the individual IGS transcripts (light orange/green boxes) from different tissues that are shown in the rows below the respective consensus rows. The names of the consensus transcripts are indicated underneath them. Tissue source and replicate number are indicated to the left. The direction of transcription is indicated by the arrowheads. The scale above shows the position in the chimpanzee rDNA IGS.
(PDF)

S15 Fig Transcripts in the orangutan IGS. Transcriptome assemblies were performed using unstranded polyA(+) RNA-seq data from the rhesus macaque tissues indicated to the left. The first row represents consensus transcripts (dark green boxes) obtained by merging the individual IGS transcripts (light green boxes) from the different tissues (rows beneath). The names of the consensus transcripts are indicated next to them. The scale above shows the position in the rhesus macaque rDNA IGS.
(PDF)

S16 Fig Transcripts in rhesus macaque IGS. Transcriptome assemblies were performed using unstranded polyA(+) RNA-seq data from the orangutan tissues indicated to the left. The first row represents consensus transcripts (dark green boxes) obtained by merging the individual IGS transcripts (light green boxes) from the different tissues (rows beneath). The names of the consensus transcripts are indicated next to them. The scale above shows the position in the orangutan rDNA IGS.
(PDF)

S17 Fig Quantification of the expression level of IGS transcripts. A) Abundance of the poly(A+) transcript splice variant from Zone-1 (represented by HUVEC poly(A+) transcript 1 in S5 Fig) that is shared between all cell lines. B) Abundance of the poly(A-) transcript from the promoter region (represented by HUVEC poly(A-) transcript 1 in S5 Fig) that is present in all cell lines. Abundances were calculated from RNA-seq data as FPKM (Fragments Per Kilobase of transcript per Million mapped reads; vertical axis) for the six cell lines (horizontal axis). The abundances represent the total expression from all rDNA units in the genome of the region of each transcript that is shared between all six cell lines. The error bars represent 95% confidence intervals for transcript abundance.
(PDF)

S1 Table Details of WGS data for the primates. (XLS)

S2 Table Assembly statistics for the primate whole genome assemblies. (XLS)

S3 Table. rDNA containing BAC clones identified by screening high-density BAC filters (XLS)

S4 Table rDNA sequence comparison between human and the six primate species. (XLS)

S5 Table The length variation between the WGA and BAC rDNA sequences of the six primate species. (XLS)

S6 Table Pairwise comparison between BAC clone rDNA sequences and WGA rDNA. (XLS)

S7 Table Pairwise sequence comparisons showing the level of sequence conservation between human and ape Alu elements. (XLS)

S8 Table Details of conserved regions in the human IGS (base position corresponds to the human rDNA sequence extracted from BAC clone GL000220.1). (XLS)

S9 Table Sequence identity of the conserved regions with common marmoset and mouse rDNA. (XLS)

S10 Table Details of long poly(A+) IGS transcripts in HUVEC cell line (base position corresponds to the human rDNA sequence extracted from BAC clone GL000220.1). (XLS)

S11 Table Details of long poly(A+) IGS transcripts in GM12878 cell line (base position corresponds to the human rDNA sequence extracted from BAC clone GL000220.1). (XLS)

S12 Table Details of long poly(A+) IGS transcripts in the H1-hESC cell line (base position corresponds to the human rDNA sequence extracted from BAC clone GL000220.1). (XLS)

S13 Table Details of long poly(A+) IGS transcripts in HepG-2 cell line (base position corresponds to the human rDNA sequence extracted from BAC clone GL000220.1). (XLS)

S14 Table Details of long poly(A+) IGS transcripts in K562 cell line (base position corresponds to the human rDNA sequence extracted from BAC clone GL000220.1). (XLS)

S15 Table Details of long poly(A+) IGS transcripts in HeLa-S3 cell line (base position corresponds to the human rDNA sequence extracted from BAC clone GL000220.1). (XLS)

S16 Table Details of long poly(A-) IGS transcripts in the HUVEC cell line (base position corresponds to the human rDNA sequence extracted from BAC clone GL000220.1). (XLS)

S17 Table Details of long poly(A-) IGS transcripts in the GM12878 cell line (base position corresponds to the human rDNA sequence extracted from BAC clone GL000220.1). (XLS)

S18 Table Details of long poly(A-) IGS transcripts in the H1-hESC cell line (base position corresponds to the human rDNA sequence extracted from BAC clone GL000220.1). (XLS)

S19 Table Details of long poly(A-) IGS transcripts in the HepG-2 cell line (base position corresponds to the human rDNA sequence extracted from BAC clone GL000220.1). (XLS)

S20 Table Details of long poly(A-) IGS transcripts in the K562 cell line (base position corresponds to the human rDNA sequence extracted from BAC clone GL000220.1). (XLS)

S21 Table Details of long poly(A-) IGS transcripts in the HeLa-S3 cell line (base position corresponds to the human rDNA sequence extracted from BAC clone GL000220.1). (XLS)

S22 Table Details of the CAGE peaks in the human IGS in the selected cell lines (base position corresponds to the human rDNA sequence extracted from BAC clone GL000220.1). (XLS)

S23 Table Coordinates of TSS, enhancer, promoters and CTCF sites in human rDNA predicted by Segway in different cell types. (XLS)

S24 Table Details of long noncoding RNA Chimpanzee IGS transcripts. (XLS)

S25 Table Details of poly(A+) Chimpanzee IGS transcripts. (XLS)

S26 Table Details of poly(A+) Orangutan IGS transcripts. (XLS)

S27 Table Details of poly(A+) RNA Rhesus macaque IGS transcripts. (XLS)

## References

[1] Gonzalez IL, Sylvester JE (1995) Complete sequence of the 43-kb human ribosomal DNA repeat: analysis of the intergenic spacer. Genomics 27: 320–328. 10.1006/geno.1995.1049 7557999 10.1006/geno.1995.1049

[2] Henderson AS, Warburton D, Atwood KC (1972) Location of ribosomal DNA in the human chromosome complement. Proc Natl Acad Sci U S A 69: 3394–3398. 4508329 10.1073/pnas.69.11.3394 PMC389778

[3] Tantravahi R, Miller DA, Dev VG, Miller OJ (1976) Detection of nucleolus organizer regions in chromosomes of human, chimpanzee, gorilla, orangutan and gibbon. Chromosoma 56: 15–27. 61844 10.1007/BF00293725

[4] Schmickel RD (1973) Quantitation of human ribosomal DNA: hybridization of human DNA with ribosomal RNA for quantitation and fractionation. Pediatr Res 7: 5–12. 10.1203/00006450-197301000-00002 4687000 10.1203/00006450-197301000-00002

[5] Stults DM, Killen MW, Pierce HH, Pierce AJ (2008) Genomic architecture and inheritance of human ribosomal RNA gene clusters. Genome Research 18: 13–18. 10.1101/gr.6858507 18025267 10.1101/gr.6858507 PMC2134781

[6] Gibbons JG, Branco AT, Yu S, Lemos B (2014) Ribosomal DNA copy number is coupled with gene expression variation and mitochondrial abundance in humans. Nat Commun 5: 4850 10.1038/ncomms5850 25209200 10.1038/ncomms5850

[7] Parks MM, Kurylo CM, Dass RA, Bojmar L, Lyden D, et al (2018) Variant ribosomal RNA alleles are conserved and exhibit tissue-specific expression. Sci Adv 4: eaao0665 10.1126/sciadv.aao0665 29503865 10.1126/sciadv.aao0665 PMC5829973

[8] Hannan KM, Hannan RD, Rothblum LI (1998) Transcription by RNA polymerase I. Proteins 4: 3. 10.2741/a282 9514985

[9] Russell J, Zomerdijk JC (2005) RNA-polymerase-I-directed rDNA transcription, life and works. Trends Biochem Sci 30: 87–96. 10.1016/j.tibs.2004.12.008 15691654 10.1016/j.tibs.2004.12.008 PMC3858833

[10] Grummt I (2013) The nucleolus-guardian of cellular homeostasis and genome integrity. Chromosoma 122: 487–497. 10.1007/s00412-013-0430-0 24022641 10.1007/s00412-013-0430-0

[11] Kobayashi T, Ganley AR (2005) Recombination regulation by transcription-induced cohesin dissociation in rDNA repeats. Science 309: 1581–1584. 10.1126/science.1116102 16141077 10.1126/science.1116102

[12] Paredes S, Maggert KA (2009) Ribosomal DNA contributes to global chromatin regulation. Proc Natl Acad Sci U S A 106: 17829–17834. 10.1073/pnas.0906811106 19822756 10.1073/pnas.0906811106 PMC2764911

[13] Derenzini M, Montanaro L, Chilla A, Tosti E, Vici M, et al (2005) Key role of the achievement of an appropriate ribosomal RNA complement for G1-S phase transition in H4-II-E-C3 rat hepatoma cells. J Cell Physiol 202: 483–491. 10.1002/jcp.20144 15389582 10.1002/jcp.20144

[14] Deisenroth C, Zhang Y (2010) Ribosome biogenesis surveillance: probing the ribosomal protein-Mdm2-p53 pathway. Oncogene 29: 4253–4260. 10.1038/onc.2010.189 20498634 10.1038/onc.2010.189

[15] Boisvert FM, van Koningsbruggen S, Navascues J, Lamond AI (2007) The multifunctional nucleolus. Nat Rev Mol Cell Biol 8: 574–585. 10.1038/nrm2184 17519961 10.1038/nrm2184

[16] Sirri V, Urcuqui-Inchima S, Roussel P, Hernandez-Verdun D (2008) Nucleolus: the fascinating nuclear body. Histochem Cell Biol 129: 13–31. 10.1007/s00418-007-0359-6 18046571 10.1007/s00418-007-0359-6 PMC2137947

[17] Ma H, Pederson T (2008) Nucleostemin: a multiplex regulator of cell-cycle progression. Trends in cell biology 18: 575–579. 10.1016/j.tcb.2008.09.003 18951797 10.1016/j.tcb.2008.09.003

[18] Audas TE, Jacob MD, Lee S (2012) Immobilization of proteins in the nucleolus by ribosomal intergenic spacer noncoding RNA. Mol Cell 45: 147–157. 10.1016/j.molcel.2011.12.012 22284675 10.1016/j.molcel.2011.12.012

[19] Zhang LF, Huynh KD, Lee JT (2007) Perinucleolar targeting of the inactive X during S phase: evidence for a role in the maintenance of silencing. Cell 129: 693–706. 10.1016/j.cell.2007.03.036 17512404 10.1016/j.cell.2007.03.036

[20] Gottlieb S, Esposito RE (1989) A new role for a yeast transcriptional silencer gene, SIR2, in regulation of recombination in ribosomal DNA. Cell 56: 771–776. 2647300 10.1016/0092-8674(89)90681-8

[21] Ganley AR, Kobayashi T (2014) Ribosomal DNA and cellular senescence: new evidence supporting the connection between rDNA and aging. FEMS yeast research 14: 49–59. 10.1111/1567-1364.12133 24373458 10.1111/1567-1364.12133

[22] Sinclair DA, Guarente L (1997) Extrachromosomal rDNA circles—a cause of aging in yeast. Cell 91: 1033–1042. 9428525 10.1016/s0092-8674(00)80493-6

[23] Nemeth A, Conesa A, Santoyo-Lopez J, Medina I, Montaner D, et al (2010) Initial genomics of the human nucleolus. PLoS Genet 6: e1000889 10.1371/journal.pgen.1000889 20361057 10.1371/journal.pgen.1000889 PMC2845662

[24] Yu S, Lemos B (2018) The long-range interaction map of ribosomal DNA arrays. PLoS Genet 14: e1007258 10.1371/journal.pgen.1007258 29570716 10.1371/journal.pgen.1007258 PMC5865718

[25] Uemura M, Zheng Q, Koh CM, Nelson WG, Yegnasubramanian S, et al (2012) Overexpression of ribosomal RNA in prostate cancer is common but not linked to rDNA promoter hypomethylation. Oncogene 31: 1254–1263. 10.1038/onc.2011.319 21822302 10.1038/onc.2011.319 PMC3298623

[26] Bywater MJ, Pearson RB, McArthur GA, Hannan RD (2013) Dysregulation of the basal RNA polymerase transcription apparatus in cancer. Nat Rev Cancer 13: 299–314. 10.1038/nrc3496 23612459 10.1038/nrc3496

[27] White RJ (2005) RNA polymerases I and III, growth control and cancer. Nat Rev Mol Cell Biol 6: 69–78. 10.1038/nrm1551 15688068 10.1038/nrm1551

[28] Montanaro L, Trere D, Derenzini M (2008) Nucleolus, ribosomes, and cancer. Am J Pathol 173: 301–310. 10.2353/ajpath.2008.070752 18583314 10.2353/ajpath.2008.070752 PMC2475768

[29] Nakhoul H, Ke J, Zhou X, Liao W, Zeng SX, et al (2014) Ribosomopathies: mechanisms of disease. Clin Med Insights Blood Disord 7: 7–16. 10.4137/CMBD.S16952 25512719 10.4137/CMBD.S16952 PMC4251057

[30] Narla A, Ebert BL (2010) Ribosomopathies: human disorders of ribosome dysfunction. Blood 115: 3196–3205. 10.1182/blood-2009-10-178129 20194897 10.1182/blood-2009-10-178129 PMC2858486

[31] Zhang Q, Shalaby NA, Buszczak M (2014) Changes in rRNA transcription influence proliferation and cell fate within a stem cell lineage. Science 343: 298–301. 10.1126/science.1246384 24436420 10.1126/science.1246384 PMC4084784

[32] Larson DE, Xie W, Glibetic M, O'Mahony D, Sells BH, et al (1993) Coordinated decreases in rRNA gene transcription factors and rRNA synthesis during muscle cell differentiation. Proceedings of the National Academy of Sciences 90: 7933–7936. 10.1073/pnas.90.17.7933 PMC47261 8396256

[33] Hayashi Y, Kuroda T, Kishimoto H, Wang C, Iwama A, et al (2014) Downregulation of rRNA transcription triggers cell differentiation. PloS one 9: e98586 10.1371/journal.pone.0098586 24879416 10.1371/journal.pone.0098586 PMC4039485

[34] Brombin A, Joly JS, Jamen F (2015) New tricks for an old dog: ribosome biogenesis contributes to stem cell homeostasis. Curr Opin Genet Dev 34: 61–70. 10.1016/j.gde.2015.07.006 26343009 10.1016/j.gde.2015.07.006

[35] McStay B (2016) Nucleolar organizer regions: genomic 'dark matter' requiring illumination. Genes Dev 30: 1598–1610. 10.1101/gad.283838.116 27474438 10.1101/gad.283838.116 PMC4973289

[36] Floutsakou I, Agrawal S, Nguyen TT, Seoighe C, Ganley AR, et al (2013) The shared genomic architecture of human nucleolar organizer regions. Genome Res 23: 2003–2012. 10.1101/gr.157941.113 23990606 10.1101/gr.157941.113 PMC3847771

[37] Xu B, Li H, Perry JM, Singh VP, Unruh J, et al (2017) Ribosomal DNA copy number loss and sequence variation in cancer. PLoS Genet 13: e1006771 10.1371/journal.pgen.1006771 28640831 10.1371/journal.pgen.1006771 PMC5480814

[38] Gibbons JG, Branco AT, Godinho SA, Yu S, Lemos B (2015) Concerted copy number variation balances ribosomal DNA dosage in human and mouse genomes. Proc Natl Acad Sci U S A 112: 2485–2490. 10.1073/pnas.1416878112 25583482 10.1073/pnas.1416878112 PMC4345604

[39] Kim JH, Dilthey AT, Nagaraja R, Lee HS, Koren S, et al (2018) Variation in human chromosome 21 ribosomal RNA genes characterized by TAR cloning and long-read sequencing. Nucleic Acids Res 46: 6712–6725. 10.1093/nar/gky442 29788454 10.1093/nar/gky442 PMC6061828

[40] Wai HH, Vu L, Oakes M, Nomura M (2000) Complete deletion of yeast chromosomal rDNA repeats and integration of a new rDNA repeat: use of rDNA deletion strains for functional analysis of rDNA promoter elements in vivo. Nucleic Acids Research 28: 3524–3534. 10982872 10.1093/nar/28.18.3524 PMC110729

[41] Kobayashi T, Heck DJ, Nomura M, Horiuchi T (1998) Expansion and contraction of ribosomal DNA repeats in Saccharomyces cerevisiae: requirement of replication fork blocking (Fob1) protein and the role of RNA polymerase I. Genes & development 12: 3821–3830. 9869636 10.1101/gad.12.24.3821 PMC317266

[42] Oakes M, Siddiqi I, Vu L, Aris J, Nomura M (1999) Transcription factor UAF, expansion and contraction of ribosomal DNA (rDNA) repeats, and RNA polymerase switch in transcription of yeast rDNA. Molecular and cellular biology 19: 8559–8569. 10567580 10.1128/mcb.19.12.8559 PMC84978

[43] Oakes ML, Johzuka K, Vu L, Eliason K, Nomura M (2006) Expression of rRNA genes and nucleolus formation at ectopic chromosomal sites in the yeast Saccharomyces cerevisiae. Molecular and cellular biology 26: 6223–6238. 10.1128/MCB.02324-05 16880531 10.1128/MCB.02324-05 PMC1592796

[44] Challice J, Segall J (1989) Transcription of the 5 S rRNA gene of Saccharomyces cerevisiae requires a promoter element at+ 1 and a 14-base pair internal control region. Journal of Biological Chemistry 264: 20060–20067. 2684967

[45] Veldman GM, Klootwijk J, Leer RJ, Planta R (1980) The transcription termination site of the ribosomal RNA operon in yeast. Nucleic acids research 8: 5179–5192. 6258138 10.1093/nar/8.22.5179 PMC324293

[46] Muller M, Lucchini R, Sogo JM (2000) Replication of yeast rDNA initiates downstream of transcriptionally active genes. Mol Cell 5: 767–777. 10882113 10.1016/s1097-2765(00)80317-2

[47] Miller CA, Kowalski D (1993) cis-acting components in the replication origin from ribosomal DNA of Saccharomyces cerevisiae. Molecular and cellular biology 13: 5360–5369. 8355687 10.1128/mcb.13.9.5360-5369.1993 PMC360237

[48] Nomura M, Nogi Y, Oakes M (2004) Transcription of rDNA in the yeast Saccharomyces cerevisiae In: Olson MOJ, editor. The Nucleolus: Springer Science & Business Media pp. 128–153.

[49] Brewer BJ, Fangman WL (1988) A Replication Fork Barrier at the 3 ‘ End of Yeast Ribosomal RNA Genes. Cell 56: 637–643. 10.1016/0092-8674(88)90222-x 3052854

[50] Kobayashi T, Hidaka M, Nishizawa M, Horiuchi T (1992) Identification of a site required for DNA replication fork blocking activity in the rRNA gene cluster in Saccharomyces cerevisiae. Molecular and General Genetics MGG 233: 355–362. 1620093 10.1007/BF00265431

[51] Ganley AR, Hayashi K, Horiuchi T, Kobayashi T (2005) Identifying gene-independent noncoding functional elements in the yeast ribosomal DNA by phylogenetic footprinting. Proc Natl Acad Sci U S A 102: 11787–11792. 10.1073/pnas.0504905102 16081534 10.1073/pnas.0504905102 PMC1182552

[52] Voelkel-Meiman K, Keil RL, Roeder GS (1987) Recombination-stimulating sequences in yeast ribosomal DNA correspond to sequences regulating transcription by RNA polymerase I. Cell 48: 1071–1079. 3548996 10.1016/0092-8674(87)90714-8

[53] Kobayashi T, Nomura M, Horiuchi T (2001) Identification of DNA cis Elements Essential for Expansion of Ribosomal DNA Repeats inSaccharomyces cerevisiae. Molecular and cellular biology 21: 136–147. 10.1128/MCB.21.1.136-147.2001 11113188 10.1128/MCB.21.1.136-147.2001 PMC88787

[54] Haltiner MM, Smale ST, Tjian R (1986) Two distinct promoter elements in the human rRNA gene identified by linker scanning mutagenesis. Mol Cell Biol 6: 227–235. 3785147 10.1128/mcb.6.1.227-235.1986 PMC367502

[55] Pfleiderer C, Smid A, Bartsch I, Grummt I (1990) An undecamer DNA sequence directs termination of human ribosomal gene transcription. Nucleic Acids Res 18: 4727–4736. 2395639 10.1093/nar/18.16.4727 PMC331929

[56] Gonzalez IL, Tugendreich S, Hieter P, Sylvester JE (1993) Fixation times of retroposons in the ribosomal DNA spacer of human and other primates. Genomics 18: 29–36. 10.1006/geno.1993.1423 8276415 10.1006/geno.1993.1423

[57] Grandori C, Gomez-Roman N, Felton-Edkins ZA, Ngouenet C, Galloway DA, et al (2005) c-Myc binds to human ribosomal DNA and stimulates transcription of rRNA genes by RNA polymerase I. Nat Cell Biol 7: 311–318. 10.1038/ncb1224 15723054 10.1038/ncb1224

[58] Kern SE, Kinzler KW, Bruskin A, Jarosz D, Friedman P, et al (1991) Identification of p53 as a sequence-specific DNA-binding protein. Science 252: 1708–1711. 2047879 10.1126/science.2047879

[59] Zentner GE, Saiakhova A, Manaenkov P, Adams MD, Scacheri PC (2011) Integrative genomic analysis of human ribosomal DNA. Nucleic Acids Res 39: 4949–4960. 10.1093/nar/gkq1326 21355038 10.1093/nar/gkq1326 PMC3130253

[60] Hamperl S, Wittner M, Babl V, Perez-Fernandez J, Tschochner H, et al (2013) Chromatin states at ribosomal DNA loci. Biochim Biophys Acta 1829: 405–417. 10.1016/j.bbagrm.2012.12.007 23291532 10.1016/j.bbagrm.2012.12.007

[61] McStay B, Grummt I (2008) The epigenetics of rRNA genes: from molecular to chromosome biology. Annu Rev Cell Dev Biol 24: 131–157. 10.1146/annurev.cellbio.24.110707.175259 18616426 10.1146/annurev.cellbio.24.110707.175259

[62] Tran DA, Wong TC, Schep AN, Drewell RA (2010) Characterization of an ultra-conserved putative cis-regulatory module at the mammalian telomerase reverse transcriptase gene. DNA and cell biology 29: 499–508. 10.1089/dna.2009.0994 20438356 10.1089/dna.2009.0994

[63] Bejerano G, Lowe CB, Ahituv N, King B, Siepel A, et al (2006) A distal enhancer and an ultraconserved exon are derived from a novel retroposon. Nature 441: 87–90. 10.1038/nature04696 16625209 10.1038/nature04696

[64] Nishihara H, Smit AF, Okada N (2006) Functional noncoding sequences derived from SINEs in the mammalian genome. Genome Res 16: 864–874. 10.1101/gr.5255506 16717141 10.1101/gr.5255506 PMC1484453

[65] Nielsen MM, Tehler D, Vang S, Sudzina F, Hedegaard J, et al (2014) Identification of expressed and conserved human noncoding RNAs. RNA 20: 236–251. 10.1261/rna.038927.113 24344320 10.1261/rna.038927.113 PMC3895275

[66] Tagle DA, Koop BF, Goodman M, Slightom JL, Hess DL, et al (1988) Embryonic epsilon and gamma globin genes of a prosimian primate (Galago crassicaudatus). Nucleotide and amino acid sequences, developmental regulation and phylogenetic footprints. J Mol Biol 203: 439–455. 3199442 10.1016/0022-2836(88)90011-3

[67] Batzoglou S, Jaffe DB, Stanley K, Butler J, Gnerre S, et al (2002) ARACHNE: A Whole-Genome Shotgun Assembler. Genome Research 12: 177–189. 10.1101/gr.208902 11779843 10.1101/gr.208902 PMC155255

[68] Jaffe DB, Butler J, Gnerre S, Mauceli E, Lindblad-Toh K, et al (2003) Whole-genome sequence assembly for mammalian genomes: Arachne 2. Genome Res 13: 91–96. 10.1101/gr.828403 12529310 10.1101/gr.828403 PMC430950

[69] Agrawal S, Ganley ARD (2016) Complete Sequence Construction of the Highly Repetitive Ribosomal RNA Gene Repeats in Eukaryotes Using Whole Genome Sequence Data In: Németh A, editor. The Nucleolus: Methods and Protocols. New York, NY: Springer New York pp. 161–181. 10.1007/978-1-4939-3792-9_13 27576718

[70] Cox MP, Peterson DA, Biggs PJ (2010) SolexaQA: At-a-glance quality assessment of Illumina second-generation sequencing data. BMC Bioinformatics 11: 485 10.1186/1471-2105-11-485 20875133 10.1186/1471-2105-11-485 PMC2956736

[71] Katoh K, Asimenos G, Toh H (2009) Multiple alignment of DNA sequences with MAFFT. Methods Mol Biol 537: 39–64. 10.1007/978-1-59745-251-9_3 19378139 10.1007/978-1-59745-251-9_3

[72] Wheeler TJ, Clements J, Eddy SR, Hubley R, Jones TA, et al (2013) Dfam: a database of repetitive DNA based on profile hidden Markov models. Nucleic Acids Res 41: D70–82. 10.1093/nar/gks1265 23203985 10.1093/nar/gks1265 PMC3531169

[73] Noe L, Kucherov G (2005) YASS: enhancing the sensitivity of DNA similarity search. Nucleic Acids Res 33: W540–543. 10.1093/nar/gki478 15980530 10.1093/nar/gki478 PMC1160238

[74] Altschul SF, Gish W, Miller W, Myers EW, Lipman DJ (1990) Basic local alignment search tool. J Mol Biol 215: 403–410. 10.1016/S0022-2836(05)80360-2 2231712 10.1016/S0022-2836(05)80360-2

[75] Katoh K, Kuma K, Toh H, Miyata T (2005) MAFFT version 5: improvement in accuracy of multiple sequence alignment. Nucleic Acids Res 33: 511–518. 10.1093/nar/gki198 15661851 10.1093/nar/gki198 PMC548345

[76] Gottgens B, Gilbert JG, Barton LM, Grafham D, Rogers J, et al (2001) Long-range comparison of human and mouse SCL loci: localized regions of sensitivity to restriction endonucleases correspond precisely with peaks of conserved noncoding sequences. Genome Res 11: 87–97. 11156618 10.1101/gr.153001 PMC311011

[77] Siepel A, Bejerano G, Pedersen JS, Hinrichs AS, Hou M, et al (2005) Evolutionarily conserved elements in vertebrate, insect, worm, and yeast genomes. Genome Res 15: 1034–1050. 10.1101/gr.3715005 16024819 10.1101/gr.3715005 PMC1182216

[78] Cooper GM, Stone EA, Asimenos G, Program NCS, Green ED, et al (2005) Distribution and intensity of constraint in mammalian genomic sequence. Genome Res 15: 901–913. 10.1101/gr.3577405 15965027 10.1101/gr.3577405 PMC1172034

[79] Dellino GI, Cittaro D, Piccioni R, Luzi L, Banfi S, et al (2013) Genome-wide mapping of human DNA-replication origins: levels of transcription at ORC1 sites regulate origin selection and replication timing. Genome Res 23: 1–11. 10.1101/gr.142331.112 23187890 10.1101/gr.142331.112 PMC3530669

[80] Dobin A, Davis CA, Schlesinger F, Drenkow J, Zaleski C, et al (2013) STAR: ultrafast universal RNA-seq aligner. Bioinformatics 29: 15–21. 10.1093/bioinformatics/bts635 23104886 10.1093/bioinformatics/bts635 PMC3530905

[81] Trapnell C, Roberts A, Goff L, Pertea G, Kim D, et al (2012) Differential gene and transcript expression analysis of RNA-seq experiments with TopHat and Cufflinks. Nat Protoc 7: 562–578. 10.1038/nprot.2012.016 22383036 10.1038/nprot.2012.016 PMC3334321

[82] Forrest AR, Kawaji H, Rehli M, Baillie JK, de Hoon MJ, et al (2014) A promoter-level mammalian expression atlas. Nature 507: 462–470. 10.1038/nature13182 24670764 10.1038/nature13182 PMC4529748

[83] Peng X, Thierry-Mieg J, Thierry-Mieg D, Nishida A, Pipes L, et al (2015) Tissue-specific transcriptome sequencing analysis expands the non-human primate reference transcriptome resource (NHPRTR). Nucleic Acids Res 43: D737–742. 10.1093/nar/gku1110 25392405 10.1093/nar/gku1110 PMC4383927

[84] Brawand D, Soumillon M, Necsulea A, Julien P, Csardi G, et al (2011) The evolution of gene expression levels in mammalian organs. Nature 478: 343–348. 10.1038/nature10532 22012392 10.1038/nature10532

[85] Encode Project Consortium (2012) An integrated encyclopedia of DNA elements in the human genome. Nature 489: 57–74. 10.1038/nature11247 22955616 10.1038/nature11247 PMC3439153

[86] Feng J, Liu T, Qin B, Zhang Y, Liu XS (2012) Identifying ChIP-seq enrichment using MACS. Nat Protoc 7: 1728–1740. 10.1038/nprot.2012.101 22936215 10.1038/nprot.2012.101 PMC3868217

[87] Koch CM, Andrews RM, Flicek P, Dillon SC, Karaoz U, et al (2007) The landscape of histone modifications across 1% of the human genome in five human cell lines. Genome Res 17: 691–707. 10.1101/gr.5704207 17567990 10.1101/gr.5704207 PMC1891331

[88] Ganley AR, Kobayashi T (2008) Phylogenetic footprinting to find functional DNA elements In: ggg, editor. Comparative Genomics: Springer pp. 367–379. 10.1007/978-1-59745-514-5_23 17993686

[89] Perelman P, Johnson WE, Roos C, Seuanez HN, Horvath JE, et al (2011) A molecular phylogeny of living primates. PLoS Genet 7: e1001342 10.1371/journal.pgen.1001342 21436896 10.1371/journal.pgen.1001342 PMC3060065

[90] Marques-Bonet T, Ryder OA, Eichler EE (2009) Sequencing primate genomes: what have we learned? Annual review of genomics and human genetics 10: 355–386. 10.1146/annurev.genom.9.081307.164420 19630567 10.1146/annurev.genom.9.081307.164420 PMC6662594

[91] Woods CG, Bond J, Enard W (2005) Autosomal recessive primary microcephaly (MCPH): a review of clinical, molecular, and evolutionary findings. The American Journal of Human Genetics 76: 717–728. 10.1086/429930 15806441 10.1086/429930 PMC1199363

[92] McGowan PO, Sasaki A, Huang TC, Unterberger A, Suderman M, et al (2008) Promoter-wide hypermethylation of the ribosomal RNA gene promoter in the suicide brain. PloS one 3: e2085 10.1371/journal.pone.0002085 18461137 10.1371/journal.pone.0002085 PMC2330072

[93] Ganley AR, Kobayashi T (2007) Highly efficient concerted evolution in the ribosomal DNA repeats: total rDNA repeat variation revealed by whole-genome shotgun sequence data. Genome Research 17: 184–191. 10.1101/gr.5457707 17200233 10.1101/gr.5457707 PMC1781350

[94] Stage DE, Eickbush TH (2007) Sequence variation within the rRNA gene loci of 12 Drosophila species. Genome research 17: 1888–1897. 10.1101/gr.6376807 17989256 10.1101/gr.6376807 PMC2099596

[95] James SA, O'Kelly MJ, Carter DM, Davey RP, van Oudenaarden A, et al (2009) Repetitive sequence variation and dynamics in the ribosomal DNA array of Saccharomyces cerevisiae as revealed by whole-genome resequencing. Genome Res 19: 626–635. 10.1101/gr.084517.108 19141593 10.1101/gr.084517.108 PMC2665781

[96] Warburton D, Henderson AS, Atwood KC (1975) Localization of rDNA and Giemsa-banded chromosome complement of white-handed gibbon, Hylobates lar. Chromosoma 51: 35–40. 49260 10.1007/BF00285805

[97] Henderson AS, Atwood KC, Warburton D (1976) Chromosomal distribution of rDNA in Pan paniscus, Gorilla gorilla beringei, and Symphalangus syndactylus: comparison to related primates. Chromosoma 59: 147–155. 1009815 10.1007/BF00328483

[98] Higuchi R, Stang HD, Browne JK, Martin MO, Huot M, et al (1981) Human ribosomal RNA gene spacer sequences are found interpersed elsewhere in the genome. Gene 15: 177–186. 6271641 10.1016/0378-1119(81)90127-x

[99] Treangen TJ, Salzberg SL (2012) Repetitive DNA and next-generation sequencing: computational challenges and solutions. Nat Rev Genet 13: 36–46. 10.1038/nrg3117 PMC3324860 22124482

[100] Hillis DM, Dixon MT (1991) Ribosomal DNA: molecular evolution and phylogenetic inference. Q Rev Biol 66: 411–453. 1784710 10.1086/417338

[101] Gonzalez IL, Sylvester JE, Smith TF, Stambolian D, Schmickel RD (1990) Ribosomal RNA gene sequences and hominoid phylogeny. Mol Biol Evol 7: 203–219. 10.1093/oxfordjournals.molbev.a040600 2359361 10.1093/oxfordjournals.molbev.a040600

[102] Gray MW, Schnare MN (1990) Evolution of the modular structure of rRNA In: Hill WE, Dahlberg A, Garrett RA, Moore PB, Schlessinger D et al, editors. The Ribosome: Structure, Function, & Evolution: American Society for Microbiology, Washington, D.C. pp. 589–597.

[103] Sáfrány G, Kominami R, Muramatsu M, Hidvégi EJ (1989) Transcription of human rihosomal DNA may terminate at multiple sites. Gene 79: 299–307. 2792766 10.1016/0378-1119(89)90212-6

[104] Mayer C, Neubert M, Grummt I (2008) The structure of NoRC-associated RNA is crucial for targeting the chromatin remodelling complex NoRC to the nucleolus. EMBO Rep 9: 774–780. 10.1038/embor.2008.109 18600236 10.1038/embor.2008.109 PMC2515205

[105] Jacob MD, Audas TE, Mullineux ST, Lee S (2012) Where no RNA polymerase has gone before: novel functional transcripts derived from the ribosomal intergenic spacer. Nucleus 3: 315–319. 10.4161/nucl.20585 22688644 10.4161/nucl.20585

[106] Mayer C, Schmitz KM, Li J, Grummt I, Santoro R (2006) Intergenic transcripts regulate the epigenetic state of rRNA genes. Mol Cell 22: 351–361. 10.1016/j.molcel.2006.03.028 16678107 10.1016/j.molcel.2006.03.028

[107] Saka K, Ide S, Ganley AR, Kobayashi T (2013) Cellular senescence in yeast is regulated by rDNA noncoding transcription. Current Biology 23: 1794–1798. 10.1016/j.cub.2013.07.048 23993840 10.1016/j.cub.2013.07.048

[108] Houseley J, Kotovic K, El Hage A, Tollervey D (2007) Trf4 targets ncRNAs from telomeric and rDNA spacer regions and functions in rDNA copy number control. EMBO J 26: 4996–5006. 10.1038/sj.emboj.7601921 18007593 10.1038/sj.emboj.7601921 PMC2080816

[109] Djebali S, Davis CA, Merkel A, Dobin A, Lassmann T, et al (2012) Landscape of transcription in human cells. Nature 489: 101–108. 10.1038/nature11233 22955620 10.1038/nature11233 PMC3684276

[110] Ong CT, Corces VG (2014) CTCF: an architectural protein bridging genome topology and function. Nat Rev Genet 15: 234–246. 10.1038/nrg3663 24614316 10.1038/nrg3663 PMC4610363

[111] Millau JF, Gaudreau L (2011) CTCF, cohesin, and histone variants: connecting the genome. Biochem Cell Biol 89: 505–513. 10.1139/o11-052 21970734 10.1139/o11-052

[112] Phillips JE, Corces VG (2009) CTCF: master weaver of the genome. Cell 137: 1194–1211. 10.1016/j.cell.2009.06.001 19563753 10.1016/j.cell.2009.06.001 PMC3040116

[113] Hoffman MM, Buske OJ, Wang J, Weng Z, Bilmes JA, et al (2012) Unsupervised pattern discovery in human chromatin structure through genomic segmentation. Nat Methods 9: 473–476. 10.1038/nmeth.1937 22426492 10.1038/nmeth.1937 PMC3340533

[114] Chen T, Dent SY (2014) Chromatin modifiers and remodellers: regulators of cellular differentiation. Nat Rev Genet 15: 93–106. 10.1038/nrg3607 24366184 10.1038/nrg3607 PMC3999985

[115] Gangwal K, Sankar S, Hollenhorst PC, Kinsey M, Haroldsen SC, et al (2008) Microsatellites as EWS/FLI response elements in Ewing's sarcoma. Proc Natl Acad Sci U S A 105: 10149–10154. 10.1073/pnas.0801073105 18626011 10.1073/pnas.0801073105 PMC2481306

[116] Gangwal K, Lessnick SL (2008) Microsatellites are EWS/FLI response elements: genomic "junk" is EWS/FLI's treasure. Cell Cycle 7: 3127–3132. 10.4161/cc.7.20.6892 18927503 10.4161/cc.7.20.6892

[117] Meloni R, Albanese V, Ravassard P, Treilhou F, Mallet J (1998) A tetranucleotide polymorphic microsatellite, located in the first intron of the tyrosine hydroxylase gene, acts as a transcription regulatory element in vitro. Hum Mol Genet 7: 423–428. 9466999 10.1093/hmg/7.3.423

[118] Iglesias AR, Kindlund E, Tammi M, Wadelius C (2004) Some microsatellites may act as novel polymorphic cis-regulatory elements through transcription factor binding. Gene 341: 149–165. 10.1016/j.gene.2004.06.035 15474298 10.1016/j.gene.2004.06.035

[119] Li Y-C, Korol AB, Fahima T, Nevo E (2004) Microsatellites within genes: structure, function, and evolution. Molecular biology and evolution 21: 991–1007. 10.1093/molbev/msh073 14963101 10.1093/molbev/msh073

[120] Van't Hof J, Hernandez P, Bjerknes CA, Lamm SS (1987) Location of the replication origin in the 9-kb repeat size class of rDNA in pea (Pisum sativum). Plant Mol Biol 9: 87–95. 10.1007/BF00015641 24276898 10.1007/BF00015641

[121] Brewer BJ, Fangman WL (1991) Mapping replication origins in yeast chromosomes. Bioessays 13: 317–322. 10.1002/bies.950130702 1759974 10.1002/bies.950130702

[122] Gencheva M, Anachkova B, Russev G (1996) Mapping the sites of initiation of DNA replication in rat and human rRNA genes. J Biol Chem 271: 2608–2614. 8576229 10.1074/jbc.271.5.2608

[123] Gogel E, Langst G, Grummt I, Kunkel E, Grummt F (1996) Mapping of replication initiation sites in the mouse ribosomal gene cluster. Chromosoma 104: 511–518. 8625739 10.1007/BF00352115

[124] Hyrien O, Mechali M (1993) Chromosomal replication initiates and terminates at random sequences but at regular intervals in the ribosomal DNA of Xenopus early embryos. The EMBO journal 12: 4511 8223461 10.1002/j.1460-2075.1993.tb06140.x PMC413880

[125] Coffman FD, Georgoff I, Fresa KL, Sylvester J, Gonzalez I, et al (1993) In vitro replication of plasmids containing human ribosomal gene sequences: origin localization and dependence on an aprotinin-binding cytosolic protein. Exp Cell Res 209: 123–132. 10.1006/excr.1993.1292 7693499 10.1006/excr.1993.1292

[126] Little RD, Platt TH, Schildkraut CL (1993) Initiation and termination of DNA replication in human rRNA genes. Mol Cell Biol 13: 6600–6613. 8413256 10.1128/mcb.13.10.6600-6613.1993 PMC364718

[127] Yoon Y, Sanchez JA, Brun C, Huberman JA (1995) Mapping of replication initiation sites in human ribosomal DNA by nascent-strand abundance analysis. Mol Cell Biol 15: 2482–2489. 7739533 10.1128/mcb.15.5.2482 PMC230478

[128] Dimitrova DS (2011) DNA replication initiation patterns and spatial dynamics of the human ribosomal RNA gene loci. Journal of cell science 124: 2743–2752. 10.1242/jcs.082230 21807939 10.1242/jcs.082230

[129] Burkhalter MD, Sogo JM (2004) rDNA enhancer affects replication initiation and mitotic recombination: Fob1 mediates nucleolytic processing independently of replication. Mol Cell 15: 409–421. 10.1016/j.molcel.2004.06.024 15304221 10.1016/j.molcel.2004.06.024

[130] Tchurikov NA, Fedoseeva DM, Sosin DV, Snezhkina AV, Melnikova NV, et al (2015) Hot spots of DNA double-strand breaks and genomic contacts of human rDNA units are involved in epigenetic regulation. J Mol Cell Biol 7: 366–382. 10.1093/jmcb/mju038 25280477 10.1093/jmcb/mju038 PMC4524424

[131] Shyian M, Mattarocci S, Albert B, Hafner L, Lezaja A, et al (2016) Budding Yeast Rif1 Controls Genome Integrity by Inhibiting rDNA Replication. PLoS Genet 12: e1006414 10.1371/journal.pgen.1006414 27820830 10.1371/journal.pgen.1006414 PMC5098799

[132] Akamatsu Y, Kobayashi T (2015) The Human RNA Polymerase I Transcription Terminator Complex Acts as a Replication Fork Barrier That Coordinates the Progress of Replication with rRNA Transcription Activity. Mol Cell Biol 35: 1871–1881. 10.1128/MCB.01521-14 25776556 10.1128/MCB.01521-14 PMC4405639

[133] Hubisz MJ, Pollard KS (2014) Exploring the genesis and functions of Human Accelerated Regions sheds light on their role in human evolution. Curr Opin Genet Dev 29: 15–21. 10.1016/j.gde.2014.07.005 25156517 10.1016/j.gde.2014.07.005

[134] Banfai B, Jia H, Khatun J, Wood E, Risk B, et al (2012) Long noncoding RNAs are rarely translated in two human cell lines. Genome Research 22: 1646–1657. 10.1101/gr.134767.111 22955977 10.1101/gr.134767.111 PMC3431482

[135] Yang L, Duff MO, Graveley BR, Carmichael GG, Chen LL (2011) Genomewide characterization of non-polyadenylated RNAs. Genome Biol 12: R16 10.1186/gb-2011-12-2-r16 21324177 10.1186/gb-2011-12-2-r16 PMC3188798

[136] Yu F, Shen X, Fan L, Yu Z (2015) Analysis of histone modifications at human ribosomal DNA in liver cancer cell. Scientific reports 5. 10.1038/srep18100 PMC4676023 26657029

[137] Pontvianne F, Blevins T, Chandrasekhara C, Mozgova I, Hassel C, et al (2013) Subnuclear partitioning of rRNA genes between the nucleolus and nucleoplasm reflects alternative epiallelic states. Genes Dev 27: 1545–1550. 10.1101/gad.221648.113 23873938 10.1101/gad.221648.113 PMC3731543

[138] Kim TK, Hemberg M, Gray JM, Costa AM, Bear DM, et al (2010) Widespread transcription at neuronal activity-regulated enhancers. Nature 465: 182–187. 10.1038/nature09033 20393465 10.1038/nature09033 PMC3020079

[139] Lam MT, Li W, Rosenfeld MG, Glass CK (2014) Enhancer RNAs and regulated transcriptional programs. Trends Biochem Sci 39: 170–182. 10.1016/j.tibs.2014.02.007 24674738 10.1016/j.tibs.2014.02.007 PMC4266492

[140] Arner E, Daub CO, Vitting-Seerup K, Andersson R, Lilje B, et al (2015) Transcribed enhancers lead waves of coordinated transcription in transitioning mammalian cells. Science 347: 1010–1014. 10.1126/science.1259418 25678556 10.1126/science.1259418 PMC4681433

[141] Pape LK, Windle JJ, Mougey E, Sollner-Webb B (1989) The Xenopus ribosomal DNA 60-and 81-base-pair repeats are position-dependent enhancers that function at the establishment of the preinitiation complex: analysis in vivo and in an enhancer-responsive in vitro system. Molecular and cellular biology 9: 5093–5104. 2601710 10.1128/mcb.9.11.5093-5104.1989 PMC363661

[142] Kuhn A, Deppert U, Grummt I (1990) A 140-base-pair repetitive sequence element in the mouse rRNA gene spacer enhances transcription by RNA polymerase I in a cell-free system. Proc Natl Acad Sci U S A 87: 7527–7531. 2217183 10.1073/pnas.87.19.7527 PMC54780

[143] Pikaard CS, Pape LK, Henderson SL, Ryan K, Paalman MH, et al (1990) Enhancers for RNA polymerase I in mouse ribosomal DNA. Mol Cell Biol 10: 4816–4825. 2388626 10.1128/mcb.10.9.4816-4825.1990 PMC361088

[144] Dixit A, Garg LC, Chao W, Jacob ST (1987) An enhancer element in the far upstream spacer region of rat ribosomal RNA gene. J Biol Chem 262: 11616–11622. 3624227

[145] Grimaldi G, Di Nocera PP (1988) Multiple repeated units in Drosophila melanogaster ribosomal DNA spacer stimulate rRNA precursor transcription. Proc Natl Acad Sci U S A 85: 5502–5506. 2840664 10.1073/pnas.85.15.5502 PMC281785

[146] Woolnough JL, Atwood BL, Liu Z, Zhao R, Giles KE (2016) The Regulation of rRNA Gene Transcription during Directed Differentiation of Human Embryonic Stem Cells. PLoS One 11: e0157276 10.1371/journal.pone.0157276 27299313 10.1371/journal.pone.0157276 PMC4907514

[147] Zaidi SK, Boyd JR, Grandy R, Medina R, Lian JB, et al (2016) Expression of Ribosomal RNA and Protein Genes in Human Embryonic Stem Cells Is Associated with the Activating H3K4me3 Histone Mark. Journal of cellular physiology. 10.1002/jcp.25309 PMC4879020 26755341

[148] Cheutin T, O'Donohue MF, Beorchia A, Vandelaer M, Kaplan H, et al (2002) Three-dimensional organization of active rRNA genes within the nucleolus. J Cell Sci 115: 3297–3307. 12140261 10.1242/jcs.115.16.3297

[149] Shiue CN, Berkson RG, Wright AP (2009) c-Myc induces changes in higher order rDNA structure on stimulation of quiescent cells. Oncogene 28: 1833–1842. 10.1038/onc.2009.21 19270725 10.1038/onc.2009.21

[150] Nemeth A, Guibert S, Tiwari VK, Ohlsson R, Langst G (2008) Epigenetic regulation of TTF-I-mediated promoter-terminator interactions of rRNA genes. EMBO J 27: 1255–1265. 10.1038/emboj.2008.57 18354495 10.1038/emboj.2008.57 PMC2367401

[151] Shiue CN, Nematollahi-Mahani A, Wright AP (2014) Myc-induced anchorage of the rDNA IGS region to nucleolar matrix modulates growth-stimulated changes in higher-order rDNA architecture. Nucleic Acids Res 42: 5505–5517. 10.1093/nar/gku183 24609384 10.1093/nar/gku183 PMC4027186

[152] Poortinga G, Quinn LM, Hannan RD (2015) Targeting RNA polymerase I to treat MYC-driven cancer. Oncogene 34: 403–412. 10.1038/onc.2014.13 24608428 10.1038/onc.2014.13

[153] Huang K, Jia J, Wu C, Yao M, Li M, et al (2013) Ribosomal RNA gene transcription mediated by the master genome regulator protein CCCTC-binding factor (CTCF) is negatively regulated by the condensin complex. J Biol Chem 288: 26067–26077. 10.1074/jbc.M113.486175 23884423 10.1074/jbc.M113.486175 PMC3764810

[154] Ghirlando R, Felsenfeld G (2016) CTCF: making the right connections. Genes Dev 30: 881–891. 10.1101/gad.277863.116 27083996 10.1101/gad.277863.116 PMC4840295

