# Supplementary material for: The conservation landscape of the human ribosomal RNA gene repeats
Source: PLoS One. 2018 Dec 5;13(12):e0207531. doi: 10.1371/journal.pone.0207531 (PMC6281188; doi:10.1371/journal.pone.0207531)
Supplement: S4 Appendix — (DOCX) [file pone.0207531.s004.docx]

**Supporting information**


## Variation between the human rDNA (extracted from BAC clone GL000220.1) and GenBank U13369 rDNA sequences

## The coding sequences of the human rDNA and U13369 are 98.2% identical, with nucleotide variants distributed throughout the sequence. The IGS sequences are 88.3% identical, with the primary reason for the lower identity being copy number variation in previously reported R-repeats and microsatellite regions (1). Specifically, there are only two R-repeat blocks present in the human rDNA unit compared to three in U13369 (the R1-repeat block at coordinates 13,481-14,279 in U13369 is absent from the human rDNA). Furthermore, a 2 kb [TCTC]n microsatellite at 21,894-23,859 and a 50 bp [TCT]n microsatellite at 40,625-40,677 in the human rDNA are absent from U13369. Similarly, a 200 bp [TCTC]n microsatellite at 26,341-26,542 and a 165 bp [CTTG]n microsatellite at 40,106-40,271 are present in U13369 but are absent from the human rDNA

## Alu element conservation amongst the apes

Gonzalez *et. al.*(2) used Southern hybridization to demonstrate Alu element conservation in the IGS, hence they can only confirm the positional conservation of the Alu elements but not the level of sequence identity. To determine the sequence identity, human Alu elements were compared with the corresponding primate IGS Alu elements. Macaque and common marmoset rDNA sequences have higher numbers of Alu elements compared to apes, therefore it is difficult demarcate the Alu elements orthologous to a human Alu element and they were not included in this comparison. Most Alu elements have high sequence identity (from 89%-97%). The pairwise comparisons show that as we move from human to gibbon, the level of sequence identity for certain Alu elements (Alu_human_2, 11, and 18) decreases more rapidly than others, suggesting that a selective constraint exists for some of the Alu elements and therefore that they may have some functional role. Several Alu elements in the human genome are transcribed to form noncoding RNAs, therefore it is possible that the conserved Alu elements have a similar role. A previous study showed that about 30% of the gorilla genome has a higher DNA sequence identity to human or chimpanzee than human and chimpanzee have to each other, and this is more pronounced for regions surrounding the genes than for the genes themselves (3). Interestingly, Alu_human_1, 9, 15, 19, 20 and 23 have this same pattern of higher sequence identity between human and gorilla than human and chimp.

**References**

1. Gonzalez, I.L. and Sylvester, J.E. (1995) Complete sequence of the 43-kb human ribosomal DNA repeat: analysis of the intergenic spacer. *Genomics*, **27**, 320-328.

2. Gonzalez, I.L., Tugendreich, S., Hieter, P. and Sylvester, J.E. (1993) Fixation times of retroposons in the ribosomal DNA spacer of human and other primates. *Genomics*, **18**, 29-36.

3. Scally, A., Dutheil, J.Y., Hillier, L.W., Jordan, G.E., Goodhead, I., Herrero, J., Hobolth, A., Lappalainen, T., Mailund, T., Marques-Bonet, T. *et al.* (2012) Insights into hominid evolution from the gorilla genome sequence. *Nature*, **483**, 169-175.
