# Supplementary material for: The conservation landscape of the human ribosomal RNA gene repeats
Source: PLoS One. 2018 Dec 5;13(12):e0207531. doi: 10.1371/journal.pone.0207531 (PMC6281188; doi:10.1371/journal.pone.0207531)
Supplement: S5 Appendix — (PDF) [file pone.0207531.s005.pdf]

```
                  20                  40                  60                  80                  100                 120
KY962518_1  gctgacacgctgtcctctggcgacctgtcgctggagaggttgggcctccggatgcgcgcggggctctggcctaccggtgacccggctagccggccgcgctcctgcttgagccgcctgccggggcccgcgg 130
human_rDNA  gctgacacgctgtcctctggcgacctgtcgctggagaggttgggcctccggatgcgcgcggggctctggcctaccggtgacccggctagccggccgcgctcctgcttgagccgcctgccggggcccgcgg 130
            140                 160                 180                 200                 220                 240                 260
KY962518_1  gcctgctgttctctcgcgcgtccgagcgtcccgactcccggtgccggcccgggtccgggtctctgacccacccgggggcggcggggaaggcggcgagggccaccgtgccccgtgcgctctccgctgcgg 260
human_rDNA  gcctgctgttctctcgcgcgtccgagcgtcccgactcccggtgccggcccgggtccgggtctctgacccacccgggggcggcggggaaggcggcgagggccaccgtgccccgtgcgctctccgctgcgg 260
                  280                 300                 320                 340                 360                 380
KY962518_1  gcgcccggggcggccgcgacaaccccaccccgctggctccgtgccgtgcgtgtcaggcgttctcgtctccgcggggttgtccgccgccccttccccggagtggggggttggccggagccgatcggctcgc 390
human_rDNA  gcgcccggggcggccgcgacaaccccaccccgctggctccgtgccgtgcgtgtcaggcgttctcgtctccgcggggttgtccgccgccccttccccggagtggggggttggccggagccgatcggctcgc 390
            400                 420                 440                 460                 480                 500                 520
KY962518_1  tggccggccggccggcctccgctcccggggggctcttcgtgatcgatgtggtgacgtcgtgctctcccgggccgggtccgagccgcgacgggcgaggggcggacgttcgtggcgaacgggaccgtccttc 520
human_rDNA  tggccggccggccggcctccgctcccggggggctcttcgtgatcgatgtggtgacgtcgtgctctcccgggccgggtccgagccgcgacgggcgaggggcggacgttcgtggcgaacgggaccgtccttc 520
                  540                 560                 580                 600                 620                 640
KY962518_1  tcgctccgccccgcggggggtcccctcgtctctcctctccccgcccgccggcggtgcgtgtgggaaggcgtggggtgcggaccccggcccgacctcgccgtcccgcccgccgccttctgcgtcgcgggtgc 650
human_rDNA  tcgctccgccccgcggggggtcccctcgtctctcctctccccgcccgccggcggtgcgtgtgggaaggcgtggggtgcggaccccggcccgacctcgccgtcccgcccgccgccttctgcgtcgcggg-gc 649
            660                 680                 700                 720                 740                 760                 780
KY962518_1  gggccggcggggtcctctgacgcggcagacagccctcgctgtcgcctccagtggttgtcgacttgcgggcggcccccctccgcggcggtgggggtgccgtcccgccggcccgtcgtgctgccctctcggg 780
human_rDNA  gggccggcggggtcctctgacgcggcagacagccctcgctgtcgcctccagtggttgtcgacttgcgggcggcccccctccgcggcggtgggggtgccgtcccgccggcccgtcgtgctgccctctcggg 779
                  800                 820                 840                 860                 880                 900
KY962518_1  gggtttgcgcgagcgtcggctccgcctgggcccttgcggtgctcctggagcgctccgggttgtccctcaggtgcccgaggccgaacggtggtgtgtcgttcccgcccccggcgcccccctcctccggtcgc 910
human_rDNA  gggtttgcgcgagcgtcggctccgcctgggcccttgcggtgctcctggagcgctccgggttgtccctcaggtgcccgaggccgaacggtggtgtgtcgttcccgcccccggcgcccccctcctccggtcgc 909
            920                 940                 960                 980                 1,000               1,020               1,040
KY962518_1  cgccgcggtgtccgcgcgtgggtcctgagggagctcgtcggtgtggggttcggggcggtttgagtgagacgagacgagacgcgcccctcccacgcggggaagggcgcccgcctgctctcggtgagcgcac 1040
human_rDNA  cgccgcggtgtccgcgcgtgggtcctgagggagctcgtcggtgtggggttcgaggcggtttgagtgagacgagacgagacgcgcccctcccacgcggggaagggcgcccgcctgctctcggtgagcgcac 1039
                  1,060               1,080               1,100               1,120               1,140               1,160
KY962518_1  gtcccgtgctcccctctggcgggtgcgcgcgggccgtgtgagcgatcgcggtgggttcgggccggtgtgacgcgtgcgccggccggccgccgaggggctgccgttctgcctccgaccggtcgtgtgtggg 1170
human_rDNA  gtcccgtgctcccctctggcgggtgcgcgcgggccgtgtgagcgatcgcggtgggttcgggccggtgtgacgcgtgcgccggccggccgccgaggggctgccgttctgcctccgaccggtcgtgtgtggg 1169
            1,180               1,200               1,220               1,240               1,260               1,280               1,300
KY962518_1  ttgacttcggaggcgctctgcctcggaaggaaggaggtgggtggacggggggcctggtggggttgcgcgcacgcgcgcaccggccgggccccgccctgaacgcgaacgctcgaggtggccgcgcgcag 1300
human_rDNA  ttgacttcggaggcgctctgcctcggaaggaaggaggtgggtggacggggggcctggtggggttgcgcgcacgcgcgcaccggccgggccccgccctgaacgcgaacgctcgaggtggccgcgcgcag 1299
                  1,320               1,340               1,360               1,380               1,400               1,420
KY962518_1  gtgtttcctcgtaccgcagggcccccctcccttccccaggcgtccctcggcgcctctgcgggcccgaggaggagcggctggcgggtggggggagtgtgacccaccctcggtgagaaaagccttctctagcg 1430
human_rDNA  gtgtttcctcgtaccgcagggcccccctcccttccccaggcgtccctcggcgcctctgcgggcccgaggaggagcggctggcgggtggggggagtgtgacccaccctcggtgagaaaagccttctctagcg 1429
            1,440               1,460               1,480               1,500               1,520               1,540               1,560
KY962518_1  atctgagaggcgtgccttgggggtaccggatccccgggccgccgcctctgtctctgcctccgttatggtagcgctgccgtagcgacccgctcgcagaggaccctcctccgcttccccctcgacggggtt 1560
human_rDNA  atctgagaggcgtgccttgggggtaccggatccccgggccgccgcctctgtctctgcctccgttatggtagcgctgccgtagcgacccgctcgcagaggaccctcctccgcttccccctcgacggggtt 1559
                  1,580               1,600               1,620               1,640               1,660               1,680
KY962518_1  gggggggagaagcgagggttccgccggccaccgcggtggtggccgagtgcggctcgtcgcctactgtggcccgcgcctcccccttccgagtcgggggaggatcccgccgggccggcccggcg-tcccag 1689
human_rDNA  gggggggagaagcgagggttccgccggccaccgcggtggtggccgagtgcggctcgtcgcctactgtggcccgcgcctcccccttccgagtcgggggaggatcccgccgggccggcccggcgttcccag 1689
            1,700               1,720               1,740               1,760               1,780               1,800               1,820
KY962518_1  cgggttgggacgcggcggccggcgggcggtgggtgtgcgcgcccggcgctctgtccggcgcgtgactccctccgccgcgagtcggctctccgcccgctcccgtgccgagtcgtgaccggtgccgacgacc 1819
human_rDNA  cgggttgggacgcggcggccggcgggcggtgggtgtgcgcgcccggcgctctgtccggcgcgtgaccccctccgccgcgagtcggctctccgcccgctcccgtgccgagtcgtgaccggtgccgacgacc 1819
                  1,840               1,860               1,880               1,900               1,920               1,940
KY962518_1  gcgtttgcgtggcacggggtcgggcccgcctggccctgggaaagcgtcccacggtgggggcgcgccggtctcccggagcgggaccgggtcggaggatggacgagaatcacgagcgacggtggtggtggcg 1949
human_rDNA  gcgtttgcgtggcacggggtcgggcccgcctggccctgggaaagcgtcccacggtgggggcgcgccggtctcccggagcgggaccgggtcggaggatggacgagaatcacgagcgacggtggtgcgggcg 1949
            1,960               1,980               2,000               2,020               2,040               2,060               2,080
KY962518_1  tgtcgggttcgtggctgcggtcgctccggggccccccggtggcggggccccggggctcgcgaggcggttctcggtgggggccgaggccgtccggcgtcccaggcggggcgccgcgggaccgccctcgtgt 2079
human_rDNA  tgtcgggttcgtggctgcggtcgctccggggccccccggtggcggggccccggggctcgcgaggcggttctcggtgggggccgaggccgtccggcgtcccaggcggggcgccgcgggaccgccctcgtgt 2079
```

```
            2,100      2,120      2,140      2,160      2,180      2,200
KY962518_1  ctgtggcggtgggatcccgcggccgtgttttcctggtggcccggccgtgcctgaggtttctcccggagccgccgcctctgcgggctcccgggtgcccttgccctcgcggtccccggccctcgcccgtctg 2209
human_rDNA  ctgtggcggtgggatcccgcggccgtgttttcctggtggcccggccgtgcctgaggtttctccccgagccgccgcctctgcgggctcccgggtgcccttgccctcgcggtccccggccctcgcccgtctg 2209
        2,220      2,240      2,260      2,280      2,300      2,320      2,340
KY962518_1  tgccctcttccccgcccgccgcccgccgatcctcttcttccccccgagcggctcaccggcttcacgtccgttggtggccccgcctgggaccgaacccggcaccgcctcgtggggcgccgccgccggccac 2339
human_rDNA  tgccctcttccccgcccgccgcccgccgatcctcttcttccccccgagcggctcaccggcttcacgtccgttggtggccccgcctgggaccgaacccggcaccgcctcgtggggcgccgccgccggccac 2339
            2,360      2,380      2,400      2,420      2,440      2,460
KY962518_1  tgatcggcccggcgtccgcgtccccggcgcgcgccttggggaccgggtcggtggcgccccgcgtggggcccggtgggcttcccggagggttccgggggtcggcctgcggcgcgtgcgggggaggagacg 2469
human_rDNA  tgatcggcccggcgtccgcgtccccggcgcgcgccttggggaccgggtcggtggcgccccgcgtggggcccggtgggcttcccggagggttccgggggtcggcctgcggcgcgtgcgggggaggagacg 2469
        2,480      2,500      2,520      2,540      2,560      2,580      2,600
KY962518_1  gttccgggggaccggccgcgactgcggcggcggtggtgggggagccgcggggatcgccgagggccggtcggccgcccgggtgccgcgcggtgccgccggcggcggtgaggccccgcgcgtgtgtcccg 2599
human_rDNA  gttccgggggaccggccgcgactgcggcggcggtggtgggggcagccgcggggatcgccgagggccggtcggccgcccgggtgccgcgcggtgccgccggcggcggtgaggccccgcgcgtgtgtcccg 2599
            2,620      2,640      2,660      2,680      2,700      2,720
KY962518_1  gctgcggtcggccgcgctcgaggggtccccgtggcgtccccttccccgccggccgcctttctcgcgccttccccgtcgccccggcctcgcccgtggtctctcgtcttctcccggcccgctcttccgaacc 2729
human_rDNA  gccgcggtcggccgcgctcgaggggtccccgtggcgtccccttccccgccggccgcctttctcgcgccttccccgtcgccccggcctcgcccgtggtctctcgtcttctcccggcccgctcttccgaacc 2729
        2,740      2,760      2,780      2,800      2,820      2,840      2,860
KY962518_1  gggtcggcgcgtcccccgggtgcgcctcgcttcccggcctgccgcggcccttccccgaggcgtccgtcccgggcgtcggcgtcggggagagcccgtcctccccgcgtggcgtcgccccgttcggcgcgc 2859
human_rDNA  gggtcggcgcgtcccccgggtgcgcctcgcttcccggcctgccgcggcccttccccgaggcgtccgtcccgggcgtcggcgtcggggagagcccgtcctccccgcgtggcgtcgccccgttcggcgcgc 2859
            2,880      2,900      2,920      2,940      2,960      2,980
KY962518_1  gcgtgcgcccgagcgcggcccggtggtccctcccggacaggcgttcgtgcgacgtgtggcgtgggtcgacctccgccttgccggtcgctcgccctctccccgggtcgggggtggggcccggccggggc 2989
human_rDNA  gcgtgcgcccgagcgcggcccggtggtccctgccggacaggcgttcgtgcgacgtgtggcgtgggtcgacctccgccttgccggtcgctcgccctttccccgggtcgggggtggggcccggccggggc 2989
        3,000      3,020      3,040      3,060      3,080      3,100      3,120
KY962518_1  ctcggccccggtcgcggtccccgtcccgggcgggggcgggcgcgccggccggcctcggtc-gccctcccttggccgtcgtgtggcgtgtgccacccctgcgcccgcgcccgccggcggggctcggagcc 3118
human_rDNA  ctcggccccggtcgcggtccccgtcccgggcgggggcgggcgcgccggccggcctcggtcggccctcccttggccgtcgtgtggcgtgtgccacccctgcgcccgcgcccgccggcggggctcggagcc 3119
            3,140      3,160      3,180      3,200      3,220      3,240
KY962518_1  gggcttcggccgggccccgggccctcgaccggaccggtgcgcgggcgctgcggccgcacggcgcgactgtccccggccgggcaccgcggtccgcctctcgctcgccgcccggacgtcggggccgccccg 3248
human_rDNA  gggcttcggccgggccccgggccctcgaccggaccggtgcgcgggcgctgcggccgcacggcgcgactgtccccggccgggcaccgcggtccgcctctcgctcgccgcccggacgtcggggccgccccg 3249
        3,260      3,280      3,300      3,320      3,340      3,360      3,380
KY962518_1  cggggcgggcggagcgccgtccccgcctcgccgccgcccgcgggcgccggccgcgcgcgcgcgcgcgtggccgccggtccctcccggccgccgggcgcgggtcggccgtccgcctcctcgcggcgggc 3378
human_rDNA  cggggcgggcggagcgccgtccccgcctcgccgccgcccgcgggcgccggccgcgcgcgcgcgcgcgtggccgccggtccctcccggccgccgggcgcgggtcggccgtccgcctcctcgcggcgggc 3379
            3,400      3,420      3,440      3,460      3,480      3,500
KY962518_1  gcgacgaagaagcgtcgcgggtctgtggcgcggggccccggtggtcgtgtcgcgtggggggcgggtggttggggcgtccggttcgccgcgccccgccccggccccaccggtcccggccgccgcccccgcg 3508
human_rDNA  gcgacgaagaagcgtcgcgggtctgtggcgcggggccccggtggtcgtgtcgcgtggggggcgggtggttggggcgtccggttcgccgcgccccgccccggccccaccggtcccggccgccgcccccgcg 3509
        3,520      3,540      3,560      3,580      3,600      3,620      3,640
KY962518_1  cccgctcgctccctcccgtccgcccgtccgcggccgtccgtccgtccgtccgtcgtcctcctcgcttgcggggcgccgggcccgtcctcgcgaggcccccggccggccgtccggccgcgtcggggcct 3638
human_rDNA  cccgctcgctccctcccgtccgcccgtccgcggc----ccgtccgtccgtccgtcgtcctcctcgcttgcggggcgccgggcccgtcctcgcgaggcccccggccggccgtccggccgcgtcggggcct 3635
            3,660      3,680      3,700      3,720      3,740      3,760
KY962518_1  cgccgcgctctaccttacctacctggttgatcctgccagtagcatatgcttgtctcaaagattaagccatgcatgtctaagtacgcacggccggtacagtgaaactgcgaatggctcattaaatcagtta 3768
human_rDNA  cgccgcgctctaccttacctacctggttgatcctgccagtagcatatgcttgtctcaaagattaagccatgcatgtctgagtacgcacggccggtacagtgaaactgcgaatggctcattaaatcagtta 3765
        3,780      3,800      3,820      3,840      3,860      3,880      3,900
KY962518_1  tggttcctttggtcgctcgctcctctcctacttggataactgtggtaattctagagctaatacatgccgacgggcgctgacccccttcgcggggggggatgcgtgcatttatcagatcaaaaccaacccgg 3898
human_rDNA  tggttcctttggtcgctcgctcctctcctacttggataactgtggtaattctagagctaatacatgccgacgggcgctgacccccttcgcggggggggatgcgtgcatttatcagatcaaaaccaacccgg 3895
            3,920      3,940      3,960      3,980      4,000      4,020
KY962518_1  tcagcccctctccggccccggccgggggggcgggcgccggcggctttggtgactctagataacctcgggccgatcgcacgccccccgtggcggcgacgacccattcgaacgtctgccctatcaactttcga 4028
human_rDNA  tcagcccctctccggccccggccgggggggcgggcgccggcggctttggtgactctagataacctcgggccgatcgcacgccccccgtggcggcgacgacccattcgaacgtctgccctatcaactttcga 4025
        4,040      4,060      4,080      4,100      4,120      4,140      4,160
KY962518_1  tggtagtcgccgtgcctaccatggtgaccacgggtgacggggaatcagggttcgattccggagagggagcctgagaaacggctaccacatccaaggaaggcagcaggcgcgcaaattacccactcccgac 4158
human_rDNA  tggtagtcgccgtgcctaccatggtgaccacgggtgacggggaatcagggttcgattccggagagggagcctgagaaacggctaccacatccaaggaaggcagcaggcgcgcaaattacccactcccgac 4155
```

```
              4,180               4,200               4,220               4,240               4,260               4,280
KY962518_1   ccggggaggtagtgacgaaaaataacaatacaggactctttcgaggccctgtaattggaatgagtccactttaaatcctttaacgaggatccattggagggcaagtctggtgccagcagccgcggtaatt 4288
human_rDNA   ccggggaggtagtgacgaaaaataacaatacaggactctttcgaggccctgtaattggaatgagtccactttaaatcctttaacgaggatccattggagggcaagtctggtgccagcagccgcggtaatt 4285
          4,300               4,320               4,340               4,360               4,380               4,400               4,420
KY962518_1   ccagctccaatagcgtatattaaagttgctgcagttaaaaagctcgtagttggatcttgggagcgggcgggcggtccgccgcgaggcgagccaccgcccgtccccgccccttgcctctcggcgccccctc 4418
human_rDNA   ccagctccaatagcgtatattaaagttgctgcagttaaaaagctcgtagttggatcttgggagcgggcgggcggtccgccgcgaggcgagccaccgcccgtccccgccccttgcctctcggcgccccctc 4415
              4,440               4,460               4,480               4,500               4,520               4,540
KY962518_1   gatgctcttagctgagtgtcccgcggggcccgaagcgtttactttgaaaaaattagagtgttcaaagcaggcccgagccgcctggataccgcagctaggaataatggaataggaccgcggttctattttg 4548
human_rDNA   gatgctcttagctgagtgtcccgcggggcccgaagcgtttactttgaaaaaattagagtgttcaaagcaggcccgagccgcctggataccgcagctaggaataatggaataggaccgcggttctattttg 4545
          4,560               4,580               4,600               4,620               4,640               4,660               4,680
KY962518_1   ttggttttcggaactgaggccatgattaagagggacggccgggggcattcgtattgcgccgctagaggtgaaattcttggaccggcgcaagacggaccagagcgaaagcatttgccaagaatgttttcat 4678
human_rDNA   ttggttttcggaactgaggccatgattaagagggacggccgggggcattcgtattgcgccgctagaggtgaaattcttggaccggcgcaagacggaccagagcgaaagcatttgccaagaatgttttcat 4675
              4,700               4,720               4,740               4,760               4,780               4,800
KY962518_1   taatcaagaacgaaagtcggaggttcgaagacgatcagataccgtcgtagttccgaccataaacgatgccgaccggcgatgcggcggcgttattcccatgacccgccgggcagcttccgggaaaccaaag 4808
human_rDNA   taatcaagaacgaaagtcggaggttcgaagacgatcagataccgtcgtagttccgaccataaacgatgccgaccggcgatgcggcggcgttattcccatgacccgccgggcagcttccgggaaaccaaag 4805
          4,820               4,840               4,860               4,880               4,900               4,920               4,940
KY962518_1   tctttgggttccggggggagtatggttgcaaagctgaaacttaaaggaattgacggaagggcaccaccaggagtggagcctgcggcttaatttgactcaacacgggaaacctcacccggcccggacacgg 4938
human_rDNA   tctttgggttccggggggagtatggttgcaaagctgaaacttaaaggaattgacggaagggcaccaccaggagtggagcctgcggcttaatttgactcaacacgggaaacctcacccggcccggacacgg 4935
              4,960               4,980               5,000               5,020               5,040               5,060
KY962518_1   acaggattgacagattgatagctctttctcgattccgtgggtggtggtgcatggccgttcttagttggtggagcgatttgtctggttaattccgataacgaacgagactctggcatgctaactagttacg 5068
human_rDNA   acaggattgacagattgatagctctttctcgattccgtgggtggtggtgcatggccgttcttagttggtggagcgatttgtctggttaattccgataacgaacgagactctggcatgctaactagttacg 5065
          5,080               5,100               5,120               5,140               5,160               5,180               5,200
KY962518_1   cgacccccgagcggtcggcgtcccccaacttcttagagggacaagtggcgttcagccacccgagattgagcaataacaggtctgtgatgcccttagatgtccggggctgcacgcgcgctacactgactgg 5198
human_rDNA   cgacccccgagcggtcggcgtcccccaacttcttagagggacaagtggcgttcagccacccgagattgagcaataacaggtctgtgatgcccttagatgtccggggctgcacgcgcgctacactgactgg 5195
              5,220               5,240               5,260               5,280               5,300               5,320
KY962518_1   ctcagcgtgtgcctaccctacgccggcaggcgcgggtaacccgttgaaccccattcgtgatggggatcggggattgcaattattccccatgaacgaggaattcccagtaagtgcgggtcataagcttgcg 5328
human_rDNA   ctcagcgtgtgcctaccctacgccggcaggcgcgggtaacccgttgaaccccattcgtgatggggatcggggattgcaattattccccatgaacgaggaattcccagtaagtgcgggtcataagcttgcg 5325
          5,340               5,360               5,380               5,400               5,420               5,440               5,460
KY962518_1   ttgattaagtccctgccctttgtacacaccgcccgtcgctactaccgattggatggtttagtgaggccctcggatcggccccgccggggtcggcccacggccctggcggagcgctgagaagacggtcgaa 5458
human_rDNA   ttgattaagtccctgccctttgtacacaccgcccgtcgctactaccgattggatggtttagtgaggccctcggatcggccccgccggggtcggcccacggccctggcggagcgctgagaagacggtcgaa 5455
              5,480               5,500               5,520               5,540               5,560               5,580
KY962518_1   cttgactatctagaggaagtaaaagtcgtaacaaggtttccgtaggtgaacctgcggaaggatcattaacggagcccggagggcgaggcccgcggcgg---cgccgccgccgcgcgcttccctccgcaca 5585
human_rDNA   cttgactatctagaggaagtaaaagtcgtaacaaggtttccgtaggtgaacctgcggaaggatcattaacggagcccggagggcgaggcccgcggcggcgccgccgccgccgcgcgcttccctccgcaca 5585
          5,600               5,620               5,640               5,660               5,680               5,700               5,720
KY962518_1   cccacccccccaccgcgacgcggcgcgtgcgcgggcggggcccgcgtgcccgttcgttcgctcgctcgttcgttcgccgcccggccccgccggccgcgagagccggagaactcgggagggagacggggga 5715
human_rDNA   cccacccccccaccgcgacgcggcgcgtgcgcgggcggggcccgcgtgcccgttcgttcgctcgctcgttcgttcgccgcccggccccgccggccgcgagagccggagaactcgggagggagacggggga 5715
              5,740               5,760               5,780               5,800               5,820               5,840
KY962518_1   gagagagagagagagagaaagagaaagaagggcgtgtcgttggtgtgcgcgtgtcgtggggccggcgggcggcggggagcggtccccggcagcggccccgacggcgtgggtgtcggcgggcgcgggggcg 5845
human_rDNA   gagagagagagagagagaaagagaaagaagggcgtgtcgttggtgtgcgcgtgtcgtggggccggcgggcggcggggagcggtccccggccgcggccccgacgacgtgggtgtcggcgggcgcgggggcg 5845
          5,860               5,880               5,900               5,920               5,940               5,960               5,980
KY962518_1   gttctcggcggcgtcgcggcgggtct--gggggtctcggtgccctcctccccgccggggcccgtcgtccggccccgccgcgccggctccccgtcttcggggccggccggattcccgtcgcctccgccgcg 5973
human_rDNA   gttctcggcggcgtcgcggcgggtctgggggggtctcggtgccctcctccccgccggggcccgtcgtccggccccgccgcgccggctccccgtcttcggggccggccggattcccgtcgcctccgccgcg 5975
              6,000               6,020               6,040               6,060               6,080               6,100
KY962518_1   ccgctccgcgccgccgggcacggccccgctcgctctccccggccttcccgctagggcgtctcgagggtcgggggccggacgccggtcccctcccccgcctcctcgtccgcccccccgccgtccaggtacc 6103
human_rDNA   ccgctccgcgccgccgggcacggccccgctcgctctccccggccttcccgctagggcgtctcgagggtcgggggccggacgccggtcccctcccccgcctcctcgtccgcccccccgccgtccaggtacc 6105
          6,120               6,140               6,160               6,180               6,200               6,220               6,240
KY962518_1   tagcgcgttccggcgcggaggtttaaagaccccttggggggatcgcccgtccgcccgtgggtcgggggcggtggtgggcccgcgggggagtcccgtcgggaggggcccggcccctcccgcgcctccaccg 6233
human_rDNA   tagcgcgttccggcgcggaggtttaaagaccccttggggggatcgcccgtccgcccgtgggtcgggggcggtggtgggcccgcgggggagtcccgtcgggaggggcccggcccctcccgcgcctccaccg 6235
```

```
6,260 6,280 6,300 6,320 6,340 6,360
KY962518_1  cggactccgctccccggccggggccgcgccgccgccgccgccgcggcggccgtcgggtgggggctttacccggcggccgtcgcgcgcctgccgcgcgtgtggcgtgcgccccgcgccgtgggggcgggaa 6363
human_rDNA  cggactccgctccccggccggggccgcgccgccgccgccgccgcggcggccgtcgggtgggggctttacccggcggccgtcgcgcgcctgccgcgcgtgtggcgtgcgccccgcgccgtgggggcgggaa 6365
6,380 6,400 6,420 6,440 6,460 6,480 6,500
KY962518_1  cccccgggcgcctgtggggtggtgtccgcgctcgcccccgcgtgggcggcgcgcgcctccccgtggtgtgaaaccttccgacccctctccggagtccggtcccgtttgctgtctcgtctggccggcctga 6493
human_rDNA  cccccgggcgcctgtggggtggtgtccgcgctcgcccccgcgtgggcggcgcgcgcctccccgtggtgtgaaaccttccgacccctctccggagtccggtcccgtttgctgtctcgtctggccggcctga 6495
6,520 6,540 6,560 6,580 6,600 6,620
KY962518_1  ggcaaccccctctcctcttgg--gggggggcgggggacgtgccgcgccaggaagggcctcctcccggtgcgtcgtcgggagcgccctcgccaaatcgacctcgtacgactcttagcggtggatcactcgg 6621
human_rDNA  ggcaaccccctctcctcttgggcggggggggggggacgtgccgcgccaggaagggcctcctcccggtgcgtcgtcgggagcgccctcgccaaatcgacctcgtacgactcttagcggtggatcactcgg 6625
6,640 6,660 6,680 6,700 6,720 6,740 6,760
KY962518_1  ctcgtgcgtcgatgaagaacgcagctagctgcgagaattaatgtgaattgcaggacacattgatcatcgacacttcgaacgcacttgcggccccgggttcctcccggggctacgcctgtctgagcgtcgc 6751
human_rDNA  ctcgtgcgtcgatgaagaacgcagctagctgcgagaattaatgtgaattgcaggacacattgatcatcgacacttcgaacgcacttgcggccccgggttcctcccggggctacgcctgtctgagcgtcgc 6755
6,780 6,800 6,820 6,840 6,860 6,880
KY962518_1  ttgccgatcaatcgcccccggggggtgcctccgggctcctcggggtgcgcggctgggggttccctcgcaggcccgccgggggccctccgtccccctaagcgcagacccggcggcgtccgccctcctcttg 6881
human_rDNA  ttgccgatcaatcgcccccggggggtgcctccgggctcctcggggtgcgcggctgggggttccctcgcaggcccgccgggggccctccgtccccctaagcgcagacccggcggcgtccgccctcctcttg 6885
6,900 6,920 6,940 6,960 6,980 7,000 7,020
KY962518_1  ccgccgcgcccgccccttccccct-cccccgcgggccctgcgtggtcacgcgtcgggtggcgggggggagaggggggcgcgcccggctgagagagacggggagggcggcgccgccgccgcccgcgaagac 7010
human_rDNA  ccgccgcgcccgccccttccccctcccccgcgggccctgcgtggtcacgcgtcgggtggcgggggggagaggggggcgcgcccggctgagagagacggggagggcggcgccgccgccgcccgcgaagac 7015
7,040 7,060 7,080 7,100 7,120 7,140
KY962518_1  ggagagggaaagagagagccggctcggccgagttcccgtggccgccgcctgcggtccgggttcctccctcggggggctccctcgcgccgcgcgcggctcggggttcggggttcgtcggccccggccggg 7140
human_rDNA  ggagagggaaagagagagccggctcggccgagttcccgtggccgccgcctgcggtccgggttcctccctcggggggctccctcgcgccgcgcgcggctcggggttcggggttcgtcggccccggccggg 7145
7,160 7,180 7,200 7,220 7,240 7,260 7,280
KY962518_1  tggaaggtcccgtgcc---cgtcgtcgtcgtcgcgcgtcgtcggcggtgggggcgtgttgcgtgcggtgtggtggtgggggaggaggaaggcgggtccggaaggggaagggtgccggcggggagaga 7267
human_rDNA  tggaaggtcccgtgcccgtcgtcgtcgtcgtcgcgcgtcgtcggcggtgggggcgtgttgcgtgcggtgtggtggtgggggaggaggaaggcgggtccggaaggggaagggtgccggcggggagaga 7275
7,300 7,320 7,340 7,360 7,380 7,400
KY962518_1  gggtcgggggagcgcgtcccggtcgccgcggttcgccgcccgcccccggtggcggcccggcgtccggccgaccgccgctcccgcgcccctcctcctccccgccgcccctcctccgaggccccgcccgtc 7397
human_rDNA  gggtcgggggagcgcgtcccggtcgccgcggttcgccgcccgcccccggtggcggcccggcgtccggccgaccgccgctcccgcgcccctcctcct-ccccgccgcccctcctccgaggccccgcccgtc 7404
7,420 7,440 7,460 7,480 7,500 7,520 7,540
KY962518_1  ctcctcgccctccccgcgcgtacgcgcgcgcgcccgcccgcccggctcgcctcgcggcgcgtcggccggggccgggagcccgccccgcggcccgcccggccgcgcccgtggccgcggcgccggggttcgc 7527
human_rDNA  ctcctcgccctccccgcgcgta----cgcgcgcccgcccgcccggctcgcctcgcggcgcgtcggccggggccgggagcccgccccgcggcccgcccggccgcgcccgtggccgcggcgccggggttcgc 7530
7,560 7,580 7,600 7,620 7,640 7,660
KY962518_1  gtgtccccggcggcgacccgcgggacgccgcggtgtcgtccgccgtcgcgcgcccgcctccggctcgcggccgcgccgcgccgcgccggggccccgtcccgagcttccgcgtcggggcggggcggctccg 7657
human_rDNA  gtgtccccggcggcgacccgcgggacgccgcggtgtcgtccgccgtcgcgcgcccgcctccggctcgcggccgcgccgcgccgcgccggggccccgtcccgagcttccgcgtcggggcggggcggctccg 7660
7,680 7,700 7,720 7,740 7,760 7,780 7,800
KY962518_1  ccgccgcgtcctcggacccgtcccccgacctccgcgggggagacgggtcggggcgtgcggcgcccgtcccgccccggcccgtgcccctccctccggtcgtcccgctccggcggggcggcgcgggggtg 7787
human_rDNA  ccgccgcgtcctcggacccgtcccccgacctccgcgggggagacgggtcggggcgtgcggcgcccgtcccgccccggcccgtgcccctccctccggtcgtcccgctccggcggggcggcgcgggggtg 7790
7,820 7,840 7,860 7,880 7,900 7,920
KY962518_1  ccctcggccgcg-gctctctctcccgtcgcctctccccctcgccgggcccgtctcccgacggagcgtcgggcgggcggtcgggccggcgcgattccgtccgtccgtccgccgagcggcccgtccccctcc 7916
human_rDNA  ccgccggccgcgcgctctctctcccgtcgcctctccccctcgccgggcccgtctcccgacggagcgtcgggcgggcggtcgggccggcgcgattccgtccgtccgtccgccgagcggcccgtccccctcc 7920
7,940 7,960 7,980 8,000 8,020 8,040 8,060
KY962518_1  gagacgcgacctcagatcagacgtggcgacccgctgaatttaagcatattagtcagcggaggaaaagaaactaaccaggattccctcagtaacggcgagtgaacagggaagagcccagcgccgaatcccc 8046
human_rDNA  gagacgcgacctcagatcagacgtggcgacccgctgaatttaagcatattagtcagcggaggagaagaaactaaccaggattccctcagtaacggcgagtgaacagggaagagcccagcgccgaatcccc 8050
8,080 8,100 8,120 8,140 8,160 8,180
KY962518_1  gccccgcggcggggcgcgggacatgtggcgtacggaagacccgctccccggcgccgctcgtggggggcccaagtccttctgatcgaggcccagcccgtggacggtgtgaggccggtagcggcccccggcg 8176
human_rDNA  gccccgcggcggggcgcgggacatgtggcgtacggaagacccgctccccggcgccgctcgtggggggcccaagtccttctgatcgaggcccagcccgtggacggtgtgaggccggtagcggcccccggcg 8180
8,200 8,220 8,240 8,260 8,280 8,300 8,320
KY962518_1  cgccgggcccgggtcttcccggagtcgggttgcttgggaatgcagcccaaagcgggtggtaaactccatctaaggctaaataccggcacgagaccgatagtcaacaagtaccgtaagggaaagttgaaaa 8306
human_rDNA  cgccgggcccgggtcttcccggagtcgggttgcttgggaatgcagcccaaagcgggtggtaaactccatctaaggctaaataccggcacgagaccgatagtcaacaagtaccgtaagggaaagttgaaaa 8310
```

8,340 8,360 8,380 8,400 8,420 8,440
KY962518_1 gaactttgaagagagagttcaagagggcgtgaaaccgttaagaggtaaacgggtggggtccgcgcagtccgcccggaggattcaacccggcggcgggtccggccgtgtcggcggcccggcggatctttcc 8436
human_rDNA gaactttgaagagagagttcaagagggcgtgaaaccgttaagaggtaaacgggtggggtccgcgcagtccgcccggaggattcaacccggcggcgggtccggccgtgtcggcggcccggcggatctttcc 8440

8,460 8,480 8,500 8,520 8,540 8,560 8,580
KY962518_1 cgccccccgttcctcccgacccctccacccgccctcccttccccgccgcccctcctcctcctccccggagggggcgggctccggcgggtgcgggggtgggcgggcggggccgggggtggggtcggcggg 8566
human_rDNA cgccccccgttcctcccgacccctccacccgccctcccttccccgccgcccctcctcctcctccccggagggggcgggctccggcgggtgcgggggtgggcgggcggggccgggggtggggtcggcggg 8570

8,600 8,620 8,640 8,660 8,680 8,700
KY962518_1 ggaccgtccccgaccggcgaccggccgccgccgggcgcatttccaccgcggcggtgcgccgcgaccggctccgggacggctgggaaggcccggcggggaaggtggctcggggggccccgtccgtccgtc 8696
human_rDNA ggaccgtccccgaccggcgaccggccgccgccgggcgcatttccaccgcggcggtgcgccgcgaccggctccgggacggctgggaaggcccggcggggaaggtggctcggggggccccgtccgtccgtc 8700

8,720 8,740 8,760 8,780 8,800 8,820 8,840
KY962518_1 cgtccgtcctcctcctcccccgtctccgccccccggccccgcgtcctccctcgggagggcgcgcgggtcgg------ggcggcggcggcggtggcggcggcggcggcggcggcgggaccgaaa-cccccc 8819
human_rDNA cgtccgtcctcctcctcccccgtctccgccccccggccccgcgtcctccctcgggagggcgcgcgggtcggggcggcggcggcggcggcggtggcggcggcggcggcggcggcgggaccgaaaccccccc 8830

8,860 8,880 8,900 8,920 8,940 8,960
KY962518_1 cgagtgttacagcccccccggcagcagcactcgccgaatcccggggccgagggagcgagacccgtcgccgcgctctccccccctcccggcgcccacccccgcggggaatcccccgcgaggggggtctcccc 8949
human_rDNA cgagtgttacagcccccccggcagcagcactcgccgaatcccggggccgagggagcgagacccgtcgccgcgctctccccccctcccggcgcccacccccgcggggaatcccccgcgaggggggtctcccc 8960

8,980 9,000 9,020 9,040 9,060 9,080 9,100
KY962518_1 cgcggggggcgcgccggcgtctcctcgtgggggggccgggccaccccctcccacggcgcgaccgctctcccacccctcctccccgcgcccccgccccggcgacggggggggtgccgcgcgcgggtcgggggg 9079
human_rDNA cgcggggggcgcgccggcgtctcctcgtgggggggccgggccaccccctcccacggcgcgaccgctctcccacccctcctccccgcgcccccgccccggcgacggggggggtgccgcgcgcgggtcgggggg 9090

9,120 9,140 9,160 9,180 9,200 9,220
KY962518_1 cggggcggactgtccccagtgcgccccgggcgggtcgcgccgtcgggcccgggggggaggttctctcggggccacgcgcgcgtcccccgaagaggggggacggcggagcgagcgcacggggtcggcggcgac 9209
human_rDNA cggggcggactgtccccagtgcgccccgggcgggtcgcgccgtcgggccc-ggggggaggttctctcggggccacgcgcgcgtcccccgaagaggggggacggcggagcgagcgcacggggtcggcggcgac 9219

9,240 9,260 9,280 9,300 9,320 9,340 9,360
KY962518_1 gtcggctacccacccgacccgtcttgaaacacggaccaaggagtctaacacgtgcgcgagtcggggggctcgcacgaaagccgccgtggcgcaatgaaggtgaaggccggcgcgctcgccggccgaggtgg 9339
human_rDNA gtcggctacccacccgacccgtcttgaaacacggaccaaggagtctaacacgtgcgcgagtcggggggctcgcacgaaagccgccgtggcgcaatgaaggtgaaggccggcgcgctcgccggccgaggtgg 9349

9,380 9,400 9,420 9,440 9,460 9,480
KY962518_1 gatcccgaggcctctccagtccgccgagggcgcaccaccggcccgtctcgcccgccgcgccggggaggtggagcacgagcgcacgtgttaggacccgaaagatggtgaactatgcctgggcagggcgaag 9469
human_rDNA gatcccgaggcctctccagtccgccgagggcgcaccaccggcccgtctcgcccgccgcgccggggaggtggagcacgagcgcacgtgttaggacccgaaagatggtgaactatgcctgggcagggcgaag 9479

9,500 9,520 9,540 9,560 9,580 9,600 9,620
KY962518_1 ccagaggaaactctggtggaggtccgtagcggtcctgacgtgcaaatcggtcgtccgacctgggtataggggcgaaagactaatcgaaccatctagtagctggttccctccgaagtttccctcaggatag 9599
human_rDNA ccagaggaaactctggtggaggtccgtagcggtcctgacgtgcaaatcggtcgtccgacctgggtataggggcgaaagactaatcgaaccatctagtagctggttccctccgaagtttccctcaggatag 9609

9,640 9,660 9,680 9,700 9,720 9,740
KY962518_1 ctggcgctctcgcagacccgacgcacccccgcccacgcagttttatccggtaaagcgaatgattagaggtcttggggccgaaacgatctcaacctattctcaaactttaaatgggtaagaagcccggctcg 9729
human_rDNA ctggcgctctcgcagacccgacgcacccccgcccacgcagttttatccggtaaagcgaatgattagaggtcttggggccgaaacgatctcaacctattctcaaactttaaatgggtaagaagcccggctcg 9739

9,760 9,780 9,800 9,820 9,840 9,860 9,880
KY962518_1 ctggcgtggagccgggcgtggaatgcgagtgcctagtgggccacttttggtaagcagaactggcgctgcgggatgaaccgaacgccgggttaaggcgcccgatgccgacgctcatcagaccccagaaaag 9859
human_rDNA ctggcgtggagccgggcgtggaatgcgagtgcctagtgggccacttttggtaagcagaactggcgctgcgggatgaaccgaacgccgggttaaggcgcccgatgccgacgctcatcagaccccagaaaag 9869

9,900 9,920 9,940 9,960 9,980 10,000
KY962518_1 gtgttggttgatatagacagcaggacggtggccatggaagtcggaatccgctaaggagtgtgtaacaactcacctgccgaatcaactagccctgaaaatggatggcgctggagcgtcggcccatacccg 9989
human_rDNA gtgttggttgatatagacagcaggacggtggccatggaagtcggaatccgctaaggagtgtgtaacaactcacctgccgaatcaactagccctgaaaatggatggcgctggagcgtcggcccatacccg 9999

10,020 10,040 10,060 10,080 10,100 10,120 10,140
KY962518_1 gccgtcgccggcagtcgagagtggacgggagcggcgggggcggcgcgcgcgcgcgcgcgtgtggtgtgcgtcggagggcggcggcggcggcgg----gggtgtgtggggtcctcccc---cgcccccccc 10112
human_rDNA gccgtcgccggcagtcgagagtggacgggagcggcgggggcggcgcgcgcgcgcgcgcgtgtggtgtgcgtcggagggcggcggcggcggcggcggcgggggtgtggggtccttccccccgcccccccccc 10129

10,160 10,180 10,200 10,220 10,240 10,260
KY962518_1 cacgcctcctcccctcctcccgcccacgccccgctccccgccccccggagccccgcggacgctacgccgcgacgagtaggagggccgctgcggtgagccttgaagcctagggcgcgggcccgggtggagcc 10242
human_rDNA cacgcctcctcccctcctcccgcccacgccccgctccccgccccccggagccccgcggacgctacgccgcgacgagtaggagggccgctgcggtgagccttgaagcctagggcgcgggcccgggtggagcc 10259

10,280 10,300 10,320 10,340 10,360 10,380 10,400
KY962518_1 gccgcaggtgcagatcttggtggtagtagcaaatattcaaacgagaactttgaaggccgaagtggagaagggttccatgtgaacagcagttgaacatgggtcagtcggtcctgagagatggcgagcgcc 10372
human_rDNA gccgcaggtgcagatcttggtggtagtagcaaatattcaaacgagaactttgaaggccgaagtggagaagggttccatgtgaacagcagttgaacatgggtcagtcggtcctgagagatggcgagcgcc 10389

```
             10,420 10,440 10,460 10,480 10,500 10,520
KY962518_1   gttctgaagggacgggcgatggcctccgttgccctcggccgatcgaaagggagtcgggttcagatccccgaatccggagtggcggagatgggcgccgcgaggcgtccagtgcggtaacgcgaccgatccc 10502
human_rDNA   gttccgaagggacgggcgatggcctccgttgccctcggccgatcgaaagggagtcgggttcagatccccgaatccggagtggcggagatgggcgccgcgaggcgtccagtgcggtaacgcgaccgatccc 10519
             10,540 10,560 10,580 10,600 10,620 10,640 10,660
KY962518_1   ggagaagccggcgggagccccggggagagttctcttttctttgtgaagggcagggcgccctggaatgggttcgccccgagagaggggcccgtgccttggaaagcgtcgcggttccggcggcgtccggtga 10632
human_rDNA   ggagaagccggcgggagccccggggagagttctcttttctttgtgaagggcagggcgccctggaatgggttcgccccgagagaggggcccgtgccttggaaagcgtcgcggttccggcggcgtccggtga 10649
             10,680 10,700 10,720 10,740 10,760 10,780
KY962518_1   gctctcgctggcccttgaaaatccgggggagagggtgtaaatctcgcgccgggccgtacccatatccgcagcaggtctccaaggtgaacagcctctggcatgttggaacaatgtaggtaagggaagtcgg 10762
human_rDNA   gctctcgctggcccttgaaaatccgggggagagggtgtaaatctcgcgccgggccgtacccatatccgcagcaggtctccaaggtgaacagcctctggcatgttggaacaatgtaggtaagggaagtcgg 10779
             10,800 10,820 10,840 10,860 10,880 10,900 10,920
KY962518_1   caagccggatccgtaacttcgggataaggattggctctaagggctgggtcggtcgggctggggcgcgaagcggggctgggcgcgcgccgcggctggacgaggcgccgccgccccccccacgcccggggca 10892
human_rDNA   caagccggatccgtaacttcgggataaggattggctctaagggctgggtcggtcgggctggggcgcgaagcggggctgggcgcgcgccgcggctggacgaggcgccgccgccccccccacgcccggggca 10909
             10,940 10,960 10,980 11,000 11,020 11,040
KY962518_1   cccccctcgcggccctccccgccccaccccgcgcgcgccgctcgctccctccccaccccgcgccctctctctctctctctcccccgctccccgtcctccccccctccccggggggagcgccgcgtgggggc 11022
human_rDNA   cccccctcgcggccctccccgccccaccccgcgcgcgccgctcgctccctccccgccccgcgccctctctctctctctctcccccgctccccgtcctccccccctccccggggggagcgccgcgtgggggc 11039
             11,060 11,080 11,100 11,120 11,140 11,160 11,180
KY962518_1   ggcggcggggggagaagggtcggggcggcaggggccggcggcgg-ccgccgcggggccccggcggcgggggcacggtccccgcgaggggggcccgggcacccggggggccggcggcggcggcgactctg 11151
human_rDNA   ggcggcggggggagaagggtcggggcggcaggggccggcggcggcccgccgcggggccccggcggcgggggcacggtccccgcgaggggggcccgggcacccggggggccggcggcggcggcgactctg 11169
             11,200 11,220 11,240 11,260 11,280 11,300
KY962518_1   gacgcgagccgggcccttcccgtggatcgccccagctgcggcgggcgtcgcggccgccccccggggagcccggcgggcgccggcgcg-ccccccccccaccccacgtctcgtcgcgcgcgcgtccgctggg 11280
human_rDNA   gacgcgagccgggcccttcccgtggatcgccccagctgcggcgggcgtcgcggccgccccccggggagcccggcgggcgccggcgcgcccccccccccaccccacgtctcgtcgcgcgcgcgtccgctggg 11299
             11,320 11,340 11,360 11,380 11,400 11,420 11,440
KY962518_1   ggcggggagcggtcggcggcggcggtcggcgggcggcggggcggggcggttcgtccccccgccctacccccccggccccgtccgccccccgttccccccctcctcctcggcgcgcggcggcggcggcg-- 11408
human_rDNA   ggcggggagcggtcggcggcggcggtcggcgggcggcggggcggggcggttcgtccccccgccctacccccccggccccgtccgccccccgttccccccctcctcctcggcgcgcggcggcggcggcggc 11429
             11,460 11,480 11,500 11,520 11,540 11,560
KY962518_1   -ggcggcggaggggccgcgggccggtcccccccgccgggtccgcccccggggccgcggttccgcgcggcgcctcgcctcggccggcgcctagcagccgacttagaactggtgcggaccaggggaatccga 11537
human_rDNA   aggcggcggaggggccgcgggccggtcccccccgccgggtccgcccccggggccgcggttccgcgcggcgcctcgcctcggccggcgcctagcagccgacttagaactggtgcggaccaggggaatccga 11559
             11,580 11,600 11,620 11,640 11,660 11,680 11,700
KY962518_1   ctgtttaattaaaacaaagcatcgcgaaggcccgcggcgggtgttgacgcgatgtgatttctgcccagtgctctgaatgtcaaagtgaagaaattcaatgaagcgcgggtaaacggcgggagtaactatg 11667
human_rDNA   ctgtttaattaaaacaaagcatcgcgaaggcccgcggcgggtgttgacgcgatgtgatttctgcccagtgctctgaatgtcaaagtgaagaaattcaatgaagcgcgggtaaacggcgggagtaactatg 11689
             11,720 11,740 11,760 11,780 11,800 11,820
KY962518_1   actctcttaaggtagccaaatgcctcgtcatctaattagtgacgcgcatgaatggatgaacgagattcccactgtccctacctactatccagcgaaaccacagccaaggaacgggcttggcggaatcag 11797
human_rDNA   actctcttaaggtagccaaatgcctcgtcatctaattagtgacgcgcatgaatggatgaacgagattcccactgtccctacctactatccagcgaaaccacagccaaggaacgggcttggcggaatcag 11819
             11,840 11,860 11,880 11,900 11,920 11,940 11,960
KY962518_1   cggggaaagaagaccctgttgagcttgactctagtctggcacggtgaagagacatgagaggtgtagaataagtgggaggcccccggcgcccccccggtgtccccgcgagggccccgggggcggggtccgcc 11927
human_rDNA   cggggaaagaagaccctgttgagcttgactctagtctggcacggtgaagagacatgagaggtgtagaataagtgggaggcccccggcgcccccccggtgtccccgcgagggccccgggggcggggtccgcc 11949
             11,980 12,000 12,020 12,040 12,060 12,080
KY962518_1   ggccctgcgggccgccggtgaaataccactactctgatcgttttttcactgacccggtgaggcgggggggcgagccccgaggggctctcgcttctggcgccaagcgcccggccgcgcgccggccgggcgc 12057
human_rDNA   ggccctgcgggccgccggtgaaataccactactctgatcgttttttcactgacccggtgaggcgggggggcgagccccgaggggctctcgcttctggcgccaagcgcccggccgcgcgccggccgggcgc 12079
             12,100 12,120 12,140 12,160 12,180 12,200 12,220
KY962518_1   gacccgctccggggacagtgccaggtggggagtttgactggggcggtacacctgtcaaacggtaacgcaggtgtcctaaggcgagctcagggaggacagaaacctcccgtggagcagaagggcaaaagct 12187
human_rDNA   gacccgctccggggacagtgccaggtggggagtttgactggggcggtacacctgtcaaacggtaacgcaggtgtcctaaggcgagctcagggaggacagaaacctcccgtggagcagaagggcaaaagct 12209
             12,240 12,260 12,280 12,300 12,320 12,340
KY962518_1   cgcttgatcttgattttcagtacgaatacagaccgtgaaagcggggcctcacgatccttctgaccttttgggttttaagcaggaggtgtcagaaaagttaccacagggataactggcttgtggcggccaa 12317
human_rDNA   cgcttgatcttgattttcagtacgaatacagaccgtgaaagcggggcctcacgatccttctgaccttttgggttttaagcaggaggtgtcagaaaagttaccacagggataactggcttgtggcggccaa 12339
             12,360 12,380 12,400 12,420 12,440 12,460 12,480
KY962518_1   gcgttcatagcgacgtcgcttttgatccttcgatgtcggctcttcctatcattgtgaagcagaattcaccaagcgttggattgttcacccactaatagggaacgtgagctgggtttagaccgtcgtgag 12447
human_rDNA   gcgttcatagcgacgtcgcttttgatccttcgatgtcggctcttcctatcattgtgaagcagaattcaccaagcgttggattgttcacccactaatagggaacgtgagctgggtttagaccgtcgtgag 12469
```

```
                12,500              12,520              12,540              12,560              12,580              12,600
KY962518_1  acaggttagttttaccctactgatgatgtgttgttgccatggtaatcctgctcagtacgagaggaaccgcaggttcagacatttggtgtatgtgcttggctgaggagccaatggggcgaagctaccatct 12577
human_rDNA  acaggttagttttaccctactgatgatgtgttgttgccatggtaatcctgctcagtacgagaggaaccgcaggttcagacatttggtgtatgtgcttggctgaggagccaatggggcgaagctaccatct 12599
        12,620              12,640              12,660              12,680              12,700              12,720              12,740
KY962518_1  gtgggattatgactgaacgcctctaagtcagaatcccgcccaggcggaacgatacggcagcgccgcggagcctcggttggcctcggatagccggtcccccgcctgtccccgccggcgggccg-ccccccc 12706
human_rDNA  gtgggattatgactgaacgcctctaagtcagaatcccgcccaggcggaacgatacggcagcgccgcggagcctcggttggcctcggatagccggtcccccgcctgtccccgccggcgggccgcccccccc 12729
                12,760              12,780              12,800              12,820              12,840              12,860
KY962518_1  ctccacgcgccccgcgcgcgcgggagggcgcgtgccccgccgcgcgccgggaccggggtccggtgcggagtgcccttcgtcctgggaaacggggcgcggctggaaaggcggccgcccccctcgcccgtcac 12836
human_rDNA  ctccacgcgccccgcgcgcgcgggagggcgcgtgccccgccgcgcgccgggaccggggtccggtgcggagtgcccttcgtcctgggaaacggggcgcggccggagaggcggccgcccccctcgcccgtcac 12859
        12,880              12,900              12,920              12,940              12,960              12,980              13,000
KY962518_1  gcaccgcacgttcgtggggaacctggcgctaaaccattcgtagacgacctgcttctgggtcggggtttcgtacgtagcagagcagctccctcgctgcgatctattgaaagtcagccctcgacacaaggt 12966
human_rDNA  gcaccgcacgttcgtggggaacctggcgctaaaccattcgtagacgacctgcttctgggtcggggtttcgtacgtagcagagcagctccctcgctgcgatctattgaaagtcagccctcgacacaaggt 12989
                13,020              13,040              13,060              13,080              13,100              13,120
KY962518_1  ttgtccgcgcgcgcgcgcgcgcgtgcgtgcgggggcccggcggggcgtgcgcgtccggcgccgtccgtccttccgttcgtcttcctccctcccggcctctcccgccgaccgcggcgtggtggtgggg- 13095
human_rDNA  ttgtccgcgcgcgcgcgcgcgcgcgcgtgcgggggcccggcggggcgtgcgcgtccggcgccgtccgtccttccgttcgtcttcctccctcccggcctctcccgccgaccgcggcgtggtggtggggg 13119
        13,140              13,160              13,180              13,200              13,220              13,240              13,260
KY962518_1  -gtgggggggagggcgcgcgaccccggtcggcgcgccccgcttcttcggttcccgcctcctccccgttcaccgccggggcggctcgtccgctccgggccgggacggggtccggggagcgtggtttgggag 13224
human_rDNA  tgtgggggggagggcgcgcgaccccggtcggcgcgccccgcttcttcggttcccgcctcctccccgttcaccgccggggcggctcgtccgctccgggccgggacggggtccggggagcgtggtttgggag 13249
                13,280              13,300              13,320              13,340              13,360              13,380
KY962518_1  ccgcggaggcggccgcgccgagccgggcccgtggcccgccggtccccgtcccggggggttggccgcgcgggccccggtggggcggccacccggggtcccggccctcgcgcgtccttcctcctcgctcctcc 13354
human_rDNA  ccgcggaggcggccgcgccgagccgggcccgtggcccgccggtccccgtcccggggggttggccgcgcgggccccggtggggcggccacccggggtcccggccctcgcgcgtccttcctcctcgctcctcc 13379
        13,400              13,420              13,440              13,460              13,480              13,500              13,520
KY962518_1  gcacgggtcgaccagcagaccgcgggtggtgggcggcgggcggcgaggccccacggggcgtccgcgcacccggccgacctccgctcgtgacctctcctcggtcgggcctccggggtcgaccgcctgccgc 13484
human_rDNA  gcacgggtcgaccagcagaccgcgggtggtgggcggcgggcggcgaggccccacggggcgtccccgcacccggccgacctccgctcgcgacctctcctcggtcgggcctccggggtcgaccgcctgccgc 13509
                13,540              13,560              13,580              13,600              13,620              13,640
KY962518_1  ccgcgggcgtgagactcagccggcgtctcgccgtgtcccgggtcgaccggcgggccttctccaccgagcggcgtgtaggagtgcccgtcgggacgaaccgcaaccggagcgtccccgtctcggtcggcac 13614
human_rDNA  ccgcgggcgtgggactcagccggcgtctcgccgtgtcccgggtcgaacggcgggccttctccaccgagcggcgtgtaggagtgcccgtcgggacgaaccgcaaccggagcgtccccgtctcggtcggcac 13639
        13,660              13,680              13,700              13,720              13,740              13,760              13,780
KY962518_1  ctccggggtcgaccagctgccgcccgcgagctccggacttagccggcgcctgcacgtgtcccgggtcgaccagcaggcggccgccggacgctgcggcgcaccgacgcgagggcgtcgattcccgttcgcg 13744
human_rDNA  ctccggggtcgaccagctgccgcccgcgagctccggacttagccggcgcctgcacgtgtcccgggtcgaccagcaggcggccgccggacgctgcggcgcaccgacgcgagggcgctgattcccgttcacg 13769
                13,800              13,820              13,840              13,860              13,880              13,900
KY962518_1  cgcccgcgacctccaccggcctcggcccgcggtggagctgggaccacgcggaactccctctctcacatttttttcagccccaccgcgagtttgcgtccgcgggacttttaagagggagtcactgctgccg 13874
human_rDNA  cgcccgcgacctccaccggcctcggcccgccgtggagctgggaccacgcggaactccctctcccacatttttttcagccccaccgcgagtttgcgtccgcgggacttttaagagggagtcactgctgccg 13899
        13,920              13,940              13,960              13,980              14,000              14,020              14,040
KY962518_1  tcagccagtaatgcttcctccttttttgcttttaggttttgtcttgccttttttttttttttctttctttctttctttctttctttttctttctttctttctttctttctcgctctcgctctctccctcc 14004
human_rDNA  tcagccagtaatgcttcctccttttttgcttttaggttttgtcttgcc---------------------------------------------------------------------------------- 13947
                14,060              14,080              14,100              14,120              14,140              14,160
KY962518_1  ctcgctcgttttctctctctctcgctcttgccctctcgctctctccctcgctcgttttctctctctctctctctctctctctctgtctctcgctctcgccctctctctctctctgtctctctgtctct 14134
human_rDNA  ---------------------------------------------------------------------------------------------------------------------------------- 13947
        14,180              14,200              14,220              14,240              14,260              14,280              14,300
KY962518_1  ctctctctctctctctctctctctctctctctctgtcgctctcgccctctcgctctctctctgtctctgtctgtgtctctctctctccctccctccctccctccctccctccctccctccctccctcc 14264
human_rDNA  ---------------------------------------------------------------------------------------------------------------------------------- 13947
                14,320              14,340              14,360              14,380              14,400              14,420
KY962518_1  ccttccttggtgccttctcggctcttgagacttagccgctgtctcgccgtgccccgggtcgaccggcgggccttctccaccgagcggcgtgtaagagtgcccgtcgggacgagccggacccgccgcgtcc 14394
human_rDNA  ---------------------------------------------------------------------------------------------------------------------------------- 13947
        14,440              14,460              14,480              14,500              14,520              14,540              14,560
KY962518_1  ccgtctcggtcggcacctccggggtcgaccagctgccgcccgcgagctccggacttagccggcgtctgcacgtgtcccgggtcgaccagcaggcggccgccggacgctgcggcgcaccgacgcgagggcg 14524
human_rDNA  ---------------------------------------------------------------------------------------------------------------------------------- 13947
```

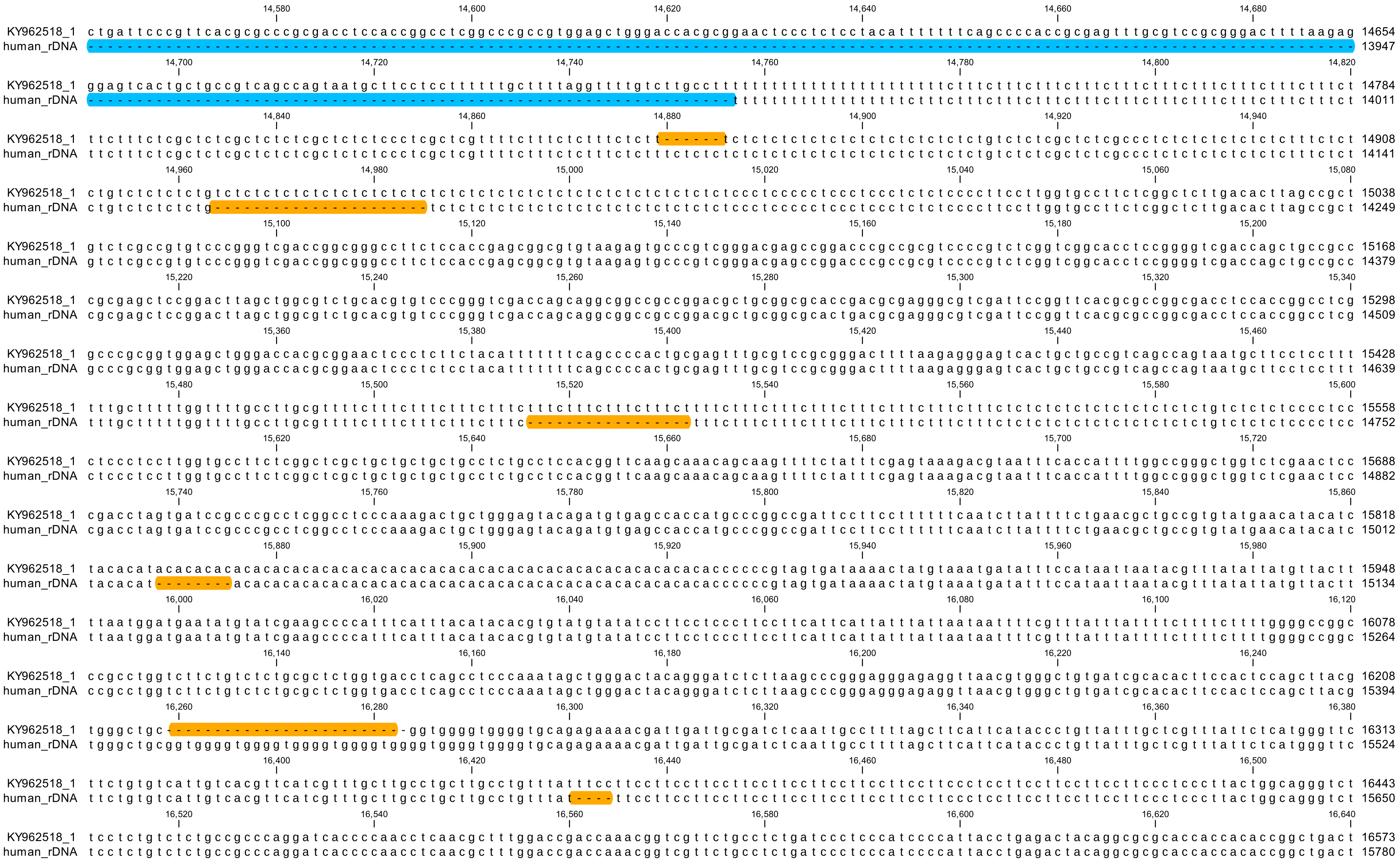
KY962518_1
human_rDNA

```
                 16,660                   16,680                   16,700                   16,720                   16,740                   16,760
KY962518_1  tttatgttgtttctcatgttttccgtaggtaggtgtgtgtgtgtgtgtgtgtgtgtgtgtgtgtgtgtgtgtgtgtgtgtgtgtgtgtatctatgtatgtacgtatgtatgtatgtatgtgagtgagatg 16703
human_rDNA  tttatgttgtttctcatgttttccgtaggtaggtatgtgtgtgtgtgtgtgtgtgtgtgtgtgtgtgtgtgtgtgtgtgtg------------tgtatctatgtatgtatgtatgtatgtgagtgagatg 15898
          16,780                   16,800                   16,820                   16,840                   16,860                   16,880                   16,900
KY962518_1  ggtttcggggttctatcatgttgcccacgctggtctcgaactcctgtcctcaagcaatccgcctgcctgcctcggccgcccacactgctgctattacaggcgtgagacgctgcgcctggctccttctaca 16833
human_rDNA  ggtttcggggttctatcatgttgcccacgctggtctcgaactcctgtcctcaagcaatccgcctgcctgcctcggccgcccacactgctgctattacaggcgtgagacgctgcgcctggctccttctaca 16028
                 16,920                   16,940                   16,960                   16,980                   17,000                   17,020
KY962518_1  tttgcctgcctgcctgcctgcctgcctgcctgcctgcctgcctgcctgcctgcctgcctatcaatcgtcttctttttagtacggatgtgctctcgctttattgtccatgctctgggcacacgtggtctct 16963
human_rDNA  tt--------tgcctgcctgcctgcctgcctgcctgcctgcctgcctgcctgcctgcctatcaatcgtcttctttttagtacggatgtgctctcgctttattgtccatgctctgggcacacgtggtctct 16150
          17,040                   17,060                   17,080                   17,100                   17,120                   17,140                   17,160
KY962518_1  tttcaaacttctatgattattattattgtaggcgtcatctcacgtgtcgaggtgatctcgaacttttaggctccagagatcctcccgcatcggcctcccggagtgctgtgatgacacgcgtgggcacggt 17093
human_rDNA  tttcaaacttctatgattattattattgtaggcgtcatctcacgtgtcgaggtgatctcgaacttttaggctccagagatcctcccgcatcggcctcccggagtgctgtgatgacacgcgtgggcacggt 16280
                 17,180                   17,200                   17,220                   17,240                   17,260                   17,280
KY962518_1  acgctctggtcgtgtttgtcgtgggtcggttctttccgttttaatacggggactgcgaacgaagaaaatttccagacgcatctcaccgatccgccttttcgttctttctttttattctctttagacgga 17223
human_rDNA  acgctctggtcgtgtttgtcgtgggtcggttctttccgttttaatacggggactgcgaacgaagaaaatttccagacgcatctcaccgatccgccttttcgttctttctttttattctctttagacgga 16410
          17,300                   17,320                   17,340                   17,360                   17,380                   17,400                   17,420
KY962518_1  gtttcactcttgtcgcccagggtggagtacgatggcggctctcggctcaccgcaccctccgcctcccaggttcaagtgattctcctgcctcagccttcccgagtagctggaatgacagagatgagccatc 17353
human_rDNA  gtttcactcttgtcgcccagggtggagtacgatggcggctctcggctcaccgcaccctccgcctcccaggttcaagtgattctcctgcctcagccttcccgagtagctggaatgacagagatgagccatc 16540
                 17,440                   17,460                   17,480                   17,500                   17,520                   17,540
KY962518_1  gtgcccggctaatttttctatttttactacagatggggtttctccatcttggtcaggctggtcttcaacttccgaccgttggagaatcttaactttcttggtggtggttgttttcctttttcttttttttt 17483
human_rDNA  gtgcccggctaatttttctatttttactacagatggggtttctccatcttggtcaggctggtcttcaacttccgaccgttggagaatcttaactttcttggtggtggttgttttcctttttcttttttttt 16670
          17,560                   17,580                   17,600                   17,620                   17,640                   17,660                   17,680
KY962518_1  cttttcttttctttccttctcctcccccccacccccccttgtcgtcgtcctcctcctcctcctcctcctcctcctcctcctcctcctcctcctcctctttcatttctttcagctgggctctcctacgtg 17613
human_rDNA  cttttcttttctttccttctcctcccccccacccccccttgtcgtcgtcctcctcctcctcctcctcctcctcctcctcctcctcctcctcctcctctttcatttctttcagctgggctctcctacgtg 16800
                 17,700                   17,720                   17,740                   17,760                   17,780                   17,800
KY962518_1  tgttgctctgttgctcacgctggtctcaaactcctggccttgacgcttctcccgtcacatccgccgtctggttgttgaaatgagcatctctcgtaaaatggaaaagatgaaagaaataaacacgaagacg 17743
human_rDNA  tgttgctctgttgctcacgctggtctcaaactcctggccttgacgcttctcccgtcacatccgccgtctggttgttgaaatgagcatctctcgtaaaatggaaaagatgaaagaaataaacacgaagacg 16930
          17,820                   17,840                   17,860                   17,880                   17,900                   17,920                   17,940
KY962518_1  gaaagcacggtgtgaacgtttctcttgccgtctcccggggtgtaccttggacccggaaacacggagggagcttggctgagtgggttttcggtgccgaaacctcccgaggcctccttccctctcccccctt 17873
human_rDNA  gaaagcacggtgtgaacgtttctcttgccgtctcccggggtgtaccttggacccggaaacacggagggagcttggctgagtgggttttcggtgccgaaacctcccgaggcctccttccctctcccccctt 17060
                 17,960                   17,980                   18,000                   18,020                   18,040                   18,060
KY962518_1  gtccccgcttctcccccagccgaggctcccaccgccgccctggcattttccataggagaggtatgggagaggactgacacgccttccagatctatatcctgccggacgtctctggctcggcgtgccccac 18003
human_rDNA  gtccccgcttctcccccagccgaggctcccaccgccgccctggcattttccataggagaggtatgggagaggactgacacgccttccagatctatatcctgccggacgtctctggctcggcgtgccccac 17190
          18,080                   18,100                   18,120                   18,140                   18,160                   18,180                   18,200
KY962518_1  cggctacctgccaccttccagggagctctgaggcggatgcgacccccacccccccgtcacgtcccgctaccctcccccggctggcctttgccgggcgaccccaggggaaccgcgttgatgctgccttcgg 18133
human_rDNA  cggctacctgccaccttccagggagctctgaggcggatgcgacccccacccccccgtcacgtcccgctaccctcccccggctggcctttgccgggcgaccccaggggaaccgcgttgatgctgccttcgg 17320
                 18,220                   18,240                   18,260                   18,280                   18,300                   18,320
KY962518_1  atcctccggcgaagacttccaccggatgccccgggtgggccggttgggatcagactggaccaccccggaccgtgctgttcttgggggtgggttgacgtacagggtggactggcagccccagcattgtaaa 18263
human_rDNA  atcctccggcgaagacttccaccggatgccccgggtgggccggttgggatcagactggaccaccccggaccgtgctgttcttgggggtgggttgacgtacagggtggactggcagccccagcattgtaaa 17450
          18,340                   18,360                   18,380                   18,400                   18,420                   18,440                   18,460
KY962518_1  gggtgcgtgggtatggaaatgtcacctaggatgccctccttcccttcggtctgccttcagctgcctcaggcgtgaagacaacttcccatcggaacctcttctcttccctttctccagcacacagatgaga 18393
human_rDNA  gggtgcgtgggtatggaaatgtcacctaggatgccctccttcccttcggtctgccttcagctgcctcaggcgtgaagacaacttcccatcggaacctcttctcttccctttctccagcacacagatgaga 17580
                 18,480                   18,500                   18,520                   18,540                   18,560                   18,580
KY962518_1  cgcacgagagggagaaacagctcaatagataccgctgaccttcatttgtggaatcctcagtcatcgacacacaagacaggtgactaggcagggacacagatcaaacactatttccgggtcctcgtggtgg 18523
human_rDNA  cgcacgagagggagaaacagctcaatagataccgctgaccttcatttgtggaatcctcagtcatcgacacacaagacaggtgactaggcagggacacagatcaaacactatttccgggtcctcgtggtgg 17710
          18,600                   18,620                   18,640                   18,660                   18,680                   18,700                   18,720
KY962518_1  gattggtctctctctctctctctctctctctctctctctctctctctcgcacgcgcacgcgcgcacacacacacacaatttccatatctagttcacagagcacactcacttccccttttcacagtacg 18653
human_rDNA  gattgg------------tctctctctctctctctctctctctctctcgcacgcgcacgcgcgcacacacacacacaatttccatatctagttcacagagcacactcacttccccttttcacagtacg 17828
```

18,740 18,760 18,780 18,800 18,820 18,840
KY962518_1 caggctgagtaaaacccgccccaccctccacccgttggctgacgaaaccccttctctacaattgatgaaaaagatgatctgggccgggcacgctagctcacgcctgtcactccggcactttgggaggccg 18783
human_rDNA caggctgagtaaaacccgccccaccctccacccgttggctgacgaaaccccttctctacaattgatgaaaaagatgatctgggccgggcacgctagctcacgcctgtcactccggcactttgggaggccg 17958

18,860 18,880 18,900 18,920 18,940 18,960 18,980
KY962518_1 aggcgggtggatcgcttggggccgggagttcgagaccaggctggccgacgtggcgaaaccccgtctctctgaaaaatagaacgattagccgggcctggtggcgtgggcttggaatcacgaccgctcggga 18913
human_rDNA aggcgggtggatcgcttggggccgggagttcgagaccaggctggccgacgtggcgaaaccccgtctctctgaaaaatagaacgattagccgggcctggtggcgtgggcttggaatcacgaccgctcggga 18088

19,000 19,020 19,040 19,060 19,080 19,100
KY962518_1 gactggggcgggcgacttgttccaaccggggaggccgaggttgcgatgagctgagatcgtgccgtggcgatgcggcctggatgacggagcgagaccccgtgtcgagagaatcatgatgttattataagat 19043
human_rDNA gactggggcgggcgacttgttccaaccggggaggccgaggttgcgatgagctgagatcgtgccgtggcgatgcggcctggatgacggagcgagaccccgtctcgagagaatcatgatgttattataagat 18218

19,120 19,140 19,160 19,180 19,200 19,220 19,240
KY962518_1 gagttgtgcgcggtgatggccgcctgtagtcgcggctactcgggaggctgagacgaggagaagatcacttgaggccccacaggtcgaggcttcggtcggccgtgacccactgtatcctgggcagtcaccg 19173
human_rDNA gagttgtgcgcggtgatggccgcctgtagtcgcggctactcgggaggctgagacgaggagaagatcacttgaggccccacaggtcgaggcttcggtcggccgtgacccactgtatcctgggcagtcaccg 18348

19,260 19,280 19,300 19,320 19,340 19,360
KY962518_1 gtcaaggagatatgccccttccccgtttgctttttcttttcttcccttctcttttcttctttttgcttctctttttctttctttctttctttctttctttctttctttctttttctttttctctcttc 19303
human_rDNA gtcaaggagatatgccccttccccgtttgctttttcttttcttcccttctcttttcttctttttgcttctctt--------tttctttctttctttctttctttctttctttttctttttctctcttc 18470

19,380 19,400 19,420 19,440 19,460 19,480 19,500
KY962518_1 ccctctttctttcctgccttcctgcctttcttcttttcttctttcctcccttcctcccttccttctttcctcccgcctcagcctcccaaagtgctgggatgactggcggaggcaccatgcctgcttggc 19433
human_rDNA ccctctttctttcctgccttcctgcctttcttcttttcttctttcctcccttcctcccttccttctttcctcccgcctcagcctcccaaagtgctgggatgactggcggaggcaccatgcctgcttggc 18600

19,520 19,540 19,560 19,580 19,600 19,620
KY962518_1 ccaaagagaccctcttggaaagtgagacgcagagagcgccttccagtgatctcattgactgatttagagacggcatctcgctccgtcaccccggcagtggtgccgtcgtaactcactccctgcagcgtgg 19563
human_rDNA ccaaagagaccctcttggaaagtgagacgcagagagcgccttccagtgatctcattgactgatttagagacggcatctcgctccgtcaccccggcagtggtgccgtcgtaactcactccctgcagcgtgg 18730

19,640 19,660 19,680 19,700 19,720 19,740 19,760
KY962518_1 acgctcctggactcgagcgatccttccacctcagcctccagagtacagagcctgggaccgcgggcacgcgccactgtgcccacaccgttttaattgtttttttttccccgagacagagtttcactctc 19693
human_rDNA acgctcctggactcgagcgatccttccacctcagcctccagagtacagagcctgggaccgcgggcacgcgccactgtgcccacaccgttttaattgtttttttttccccgagacagagtttcactctc 18860

19,780 19,800 19,820 19,840 19,860 19,880
KY962518_1 gtggcctagactgcagtgcggtggcgcgatcttggctcaccgcaacctctgcctcccggtttcaagcgattctcctgcatcggcctcctgagtagccgggattgcgggcatgcgctgccacgtctggctg 19823
human_rDNA gtggcctagactgcagtgcggtggcgcgatcttggctcaccgcaacctctgcctcccggtttcaagcgattctcctgcatcggcctcctgagtagccgggattgcgggcatgcgctgccacgtctggctg 18990

19,900 19,920 19,940 19,960 19,980 20,000 20,020
KY962518_1 atttcgtatttttagtggagacggggcttctccatgtcgatcgggctggtttcgaactcccgacctcaggtgatccgccctccccggcctccggaagtgctgggatgacaggcgtgagccaccgcgcccg 19953
human_rDNA atttcgtatttttagtggagacggggcttctccatgtcgatcgggctggtttcgaactcccgacctcaggtgatccgccctccccggcctccggaagtgctgggatgacaggcgtgagccaccgcgcccg 19120

20,040 20,060 20,080 20,100 20,120 20,140
KY962518_1 gccttcattttaaatgttttcccacagacggggtctcatcatttctttgcaaccctcctgcccggcgtctcaaagtgctggcgtgacgggcgtgagccactgcgcctggactccggggaatgactcacg 20083
human_rDNA gccttcattttaaatgttttcccacagacggggtctcatcatttctttgcaaccctcctgcccggcgtctcaaagtgctggcgtgacgggcgtgagccactgcgcctggactccggggaatgactcacg 19250

20,160 20,180 20,200 20,220 20,240 20,260 20,280
KY962518_1 accaccatcgctctactgatcctttctttctttctttctttctttctttctttctttctttctttctttctttctttctttcttgatgaattatcttatgatttatttgtgtacttattttcagacggag 20213
human_rDNA accaccatcgctctactgatc--------------------ctttctttctttctttctttctttctttctttctttctttcttgatgaattatcttatgatttatttgtgtacttattttcagacggag 19360

20,300 20,320 20,340 20,360 20,380 20,400
KY962518_1 tctcgctctgggcggggcgaggcgaggcgaggcacagcgcatcgctttggaagccgcggcaacgcctttcaaagccccattcgtatgcacagagccttattcccttcctggagttggagctgatgccttc 20343
human_rDNA tctcgctctgggcggggcgaggcgaggcgaggcacagcgcatcgctttggaagccgcggcaacgcctttcaaagccccattcgtatgcacagagccttattcccttcctggagttggagctgatgccttc 19490

20,420 20,440 20,460 20,480 20,500 20,520 20,540
KY962518_1 cgtagccttgggcttctctccattcggaagctttgacaggcgcaaccccacccagaggctggctgcggctgaggattagggggtgtgttggggctgaaaactgggtcccctatttttgatacctcagccg 20473
human_rDNA cgtagccttgggcttctctccattcggaagctttgacaggcgcaaccccacccagaggctggctgcggctgaggattagggggtgtgttggggctgaaaactgggtcccctatttttgatacctcagccg 19620

20,560 20,580 20,600 20,620 20,640 20,660
KY962518_1 acacatcccccgaccgccatcgcttgctcgccctctgagatccccgcctccaccgccttgcaggctcacctcttactttcatttcttcctttcttgcgtttgaggagggggtgcgggaatgagggtgtg 20603
human_rDNA acacatcccccgaccgccatcgcttgctcgccctctgagatccccgcctccaccgccttgcaggctcacctcttactttcatttcttcctttcttgcgtttgaggagggggtgcgggaatgagggtgtg 19750

20,680 20,700 20,720 20,740 20,760 20,780 20,800
KY962518_1 tgtggggagggggtgcggggtggggacggaggggagcgtcctaagggtcgatttagtgtcatgcctctttcaccaccaccaccaccaccgaagatgacagcaaggatcggctaaataccgcgtgttctca 20733
human_rDNA tgtggggagggggtgcggggtggggacggaggggagcgtcctaagggtcgatttagtgtcatgcctctttcaccaccaccaccaccaccgaagatgacagcaaggatcggctaaataccgcgtgttctca 19880

```
                              20,820              20,840              20,860              20,880              20,900              20,920
KY962518_1  tctagaagtgggaacttacagatgacagttcttgcatgggcagaacgagggggaccggggacgcggaagcctgcttgagggaggagggggtggaaggagagacagcttcaggaagaaaacaaaacacgaat 20863
human_rDNA  tctagaagtgggaacttacagatgacagttcttgcatgggcagaacgagggggaccggggacgcggaagtctgcttgagggaggagggggtggaaggagagacagcttcaggaagaaaacaaaacacgaat 20010
                    20,940              20,960              20,980              21,000              21,020              21,040              21,060
KY962518_1  actgtcggacacagcactgactacccgggtgatgaaatcatctgcacactgaacaccccgtcacaagtttacctatgtcacagtcttgcacatgtatgcttgaacgacaaataaaagttaggggggaga 20993
human_rDNA  actgtcggacacagcactgactacccgggtgatgaaatcatctgcacactgaacaccccgtcacaagtttacctatgtcacaatcttgcacatgtatgcttgaacgacaaataaaagttaggggggaga 20140
                              21,080              21,100              21,120              21,140              21,160              21,180
KY962518_1  agagaggagagagagagagagagagagagacagagagagacagagagagagagagaggagggagagagaaaacgaaacaccacctccttgacctgagtcagggggtttctggccttttgggagaacgttcag 21123
human_rDNA  agagaggagagagagagagagagagagagacagagagagacagagagagagagagaggagggagagagaaaacgaaacaccacctccttgacctgagtcagggggtttctggccttttgggagaacgttcag 20270
                    21,200              21,220              21,240              21,260              21,280              21,300              21,320
KY962518_1  cgacaatgcagtatttgggcccgttcttttttttttcttcttcttttctttcttttttttttggactgagtctctcgctctgtcacccaggctgcggtgcggtggcgctctctcggctcactgaaacctc 21253
human_rDNA  cgacaatgcagtatttgggcccgttcttttttttttcttcttcttttctttcttttttttttggactgagtctctcgctctgtcacccaggctgcggtgcggtggcgctctctcggctcactgaaacctc 20400
                              21,340              21,360              21,380              21,400              21,420              21,440
KY962518_1  tgcttcccgggttccagtgattcttcttcggtagctgggattacaggcgcacaccatgacggccggctcatattcctattttcagtagagacggggtttctccacgttggccacgctggtctcgaactcc 21383
human_rDNA  tgcttcccgggttccagtgattcttcttcggtagctgggattacaggcgcacaccatgacggccggctcatattcctattttcagtagagacggggtttctccacgttggccacgctggtctcgaactcc 20530
                    21,460              21,480              21,500              21,520              21,540              21,560              21,580
KY962518_1  tgacctcaaatgatccgccttcctgggcctcccaaagtgctggaaacgacaggcctgagccgccgggatttcagcctttaaaagcgcgggccctgccacctttcgctgtggcccttacgctcagaatgac 21513
human_rDNA  tgacctcaaatgatccgccttcctgggcctcccaaagtgctggaaacgacaggcctgagccgccgggatttcagcctttaaaagcgcgggccctgccacctttcgctgtggcccttacgctcagaatgac 20660
                              21,600              21,620              21,640              21,660              21,680              21,700
KY962518_1  gtgtcctctctgccgtaggttgactccttgagtcccctaggccattgcactgtagcctgggcagcaagagccaaactccgtcccccacctccccgcgcacataataactaactaacaaactaactaact 21643
human_rDNA  gtgtcctctctgccgtaggttgactccttgagtcccctaggccattgcactgtagcctgggcagcaagagccaaactccgtcccccacctccccgcgcacataataactaactaacaaactaactaact 20790
                    21,720              21,740              21,760              21,780              21,800              21,820              21,840
KY962518_1  aactaaactaactaaataaataaaatctctacacgtcacctctaagtgtgtgttcccgtgaggagtgatttctaagaaatggcactgtacactgaacgcagtggctcacgtctgtcatcccgaggtcagg 21773
human_rDNA  aactaaactaactaaataaataaaatctctacacgtcacctctaagtgtgtgttcccgtgaggagtgatttctaagaaatggcactgtacactgaacgcagtggctcacgtctgtcatcccgaggtcagg 20920
                              21,860              21,880              21,900              21,920              21,940              21,960
KY962518_1  agttcgagaccagcccggccaacgtggtgaaaccccgtctctactgaaaatacgaaatggagtcaggcgccgtggggcaggcacctgtaaccccagctactcgggaggctggggtggaagaattgcttg 21903
human_rDNA  agttcgagaccagcccggccaacgtggtgaaaccccgtctctactgaaaatacgaaatggagtcaggcgccgtggggcaggcacctgtaaccccagctactcgggaggctggggtggaagaattgcttg 21050
                    21,980              22,000              22,020              22,040              22,060              22,080              22,100
KY962518_1  aacctggcaggcggaggctgcagtgacccaagatcgcaccactgcactacagcctgggcgacagagtgagacccggtctccagataaatacgtacataaataaatacacacatacatacatacatacata 22033
human_rDNA  aacctggcaggcggaggctgcagtgacccaagatcgcaccactgcactacagcctgggcgacagagtgagacccggtctccagataaatacgtacataaataaatacacacatacatacatacatacata 21180
                              22,120              22,140              22,160              22,180              22,200              22,220
KY962518_1  catacatacatacatacatacatccatgcatacagatatacaagaaagaaaaaaagaaaagaaaagaaagagaaaatgaaagaaaaggcactgtattgctactgggctagggccttctctctgtctgttt 22163
human_rDNA  catacatacatacatacatacatccatgcatacagatatacaagaaagaaaaaaagaaaagaaaagaaagagaaaatgaaagaaaaggcactgtattgctactgggctagggccttctctctgtctgttt 21310
                    22,240              22,260              22,280              22,300              22,320              22,340              22,360
KY962518_1  ctctctgttcgtctctgtctttctctctgtgtctctttctctgtctgtctgtctctttctttctctctgtctctgtctctgtctttgtctctctctctccctctctgcctgtctcactgtgtctgtcttc 22293
human_rDNA  ctctctgttcgtctctgtctttctctctgtgtctctttctctgtctgtctgtctctttctttctctctgtctctgtctctgtctttgtctctctctctccctctctgcctgtctcactgtgtctgtcttc 21440
                              22,380              22,400              22,420              22,440              22,460              22,480
KY962518_1  tgtcttactctctttctctccccgtctgtctctctctctctctctccctccctgtttgtttctctctctccctccctgtctgtttctctctctctctttctgtctgtttctgtctctctctgtctgtcta 22423
human_rDNA  tgtcttactctctttctctccccgtctgtctctctctctctctctccctccctgtttgtttctctctctccctccctgtctgtttctctctctctctttctgtctgtttctgtctctctctgtctgtcta 21570
                    22,500              22,520              22,540              22,560              22,580              22,600              22,620
KY962518_1  tgtctttctctgtctgtctctttctctgtctgtctgcctctctctttctttttctgtgtctctctgtcggtctctctctctctgtctgtctgtctgtctctctctctctctctgtgcctatcttctgt 22553
human_rDNA  tgtctttctctgtctgtctctttctctgtctgtctgcctctctctttctttttctgtgtctctctgtcggtctctctctctctgtctgtctgtctgtctctctctctctctctgtgcctatcttctgt 21700
                              22,640              22,660              22,680              22,700              22,720              22,740
KY962518_1  cttactctctttctctgcctgtctgtctgtctctccctccctttctgtttctctctctctctctctctctctccccctctccctgtctgtttctctccgtctctctctctttctgtctgtttctcactgt 22683
human_rDNA  cttactctctttctctgcctgtctgtctgtctctccctccctttctgtttctctctctctctctctctctctccccctctccctgtctgtttctctccgtctctctctctttctgtctgtttctcactgt 21830
                    22,760              22,780              22,800              22,820              22,840              22,860              22,880
KY962518_1  ctctctctgtccatctctctctctctctgtctgtctctttcgttctctctgtctgtctgtctctctctctctctctctctctctctctctctctctctctgtctctcactctctgtgtgtatcttctgtc 22813
human_rDNA  ctctctctgtccatctctctctctctctgtctgtctctttcgttctctctgtctgtctg------tctctctctctctctctctctctctctctctttctgtctctcactctctgtgtgtatcttctgtc 21954
```

```
                         22,900                 22,920                 22,940                 22,960                 22,980                 23,000
KY962518_1   ttactctccttctctgcctgtccgtctgtctgtctgtctgtctgtctctctctccctttctgtctctctctctctctgtccctctctctttctgtctgttcctctctctctctctgtctctgtctttctc 22943
human_rDNA   ttactctccttctctgcctgtccgtctgtctgtctgtctgtctgtctctctctccctttctgtctctctctctctctgtccctctctctttctgtctgttcctctctctctctctgtctctgtctttctc 22084
              23,020   23,040   23,060   23,080   23,100   23,120   23,140
KY962518_1   tgtctgtctgcctctctctttctttctctttctgtgtctctctgtctctctctctgtgcctatcttctgtcttactctctttctctgcctgcctgcctgtctgtctgtctgtctctctctgtctctctcc 23073
human_rDNA   tgtctgtctgcctctctctttctttctctttctgtgtctctctgtctctctctctgtgcctatcttctgtcttactctctttctctgcctgcctgcctgtctgtctgtctgtctctctctgtctctctcc 22214
              23,160   23,180   23,200   23,220   23,240   23,260
KY962518_1   ctgcctttctgtttctctctctctctccctctctctctccctctctcgctctctctgtctttctctctttctctctgtttctctgtctctctctgtccgtctctgtctttttctgtctgtctctctcttt 23203
human_rDNA   ctgcctttctgtttctctctctctctccctctctctctccctctctcgctctctctgtctttctctctttctctctgtttctctgtctctctctgtccgtctctgtctttttctgtctgtctctctcttt 22344
              23,280   23,300   23,320   23,340   23,360   23,380   23,400
KY962518_1   ctttctgtctgtctctgtctctgtctctctctctctctctgcttgtctctctcactgtgtctgtcctctgtcttactctccttctctgcctgtccgtctgtctgtctgtctctctctctctctcccctccctt 23333
human_rDNA   ctttctgtctgtctctgtctctgtctctctctctctctctgcttgtctctctcactgtgtctgtcctctgtcttactctccttctctgcctgtccgtctgtctgtctgtctctctctctctctcccctccctt 22474
              23,420   23,440   23,460   23,480   23,500   23,520
KY962518_1   tctgtttctctctcgctctctctctctctctctctctctctctgcctgtttctctttctctctctgtctgtctctgtctttctctgtctgtctctttctctgtctgtctgtctcccttctctctgtctc 23463
human_rDNA   tctgtttctctctcg--ctctctctctctctctctctctctctctgcctgtttctctttctctctctgtctgtctctgtctttctctgtctgtctctttctctgtctgtctgtctcccttctctctgtctc 22602
              23,540   23,560   23,580   23,600   23,620   23,640   23,660
KY962518_1   cgtctctgtctctctctctctgtctctctctctctgcctgtctcactgtgtctgtcttctgtcttattctctttctctgtctgtctgtctctctctctcccttcctgtctctt----tctctctctctct 23589
human_rDNA   cgtctctgtctctctctctctgtctctctctctctgcctgtctcactgtgtctgtcttctgtcttattctctttctctgtctgtctgtctctctctctcccttcctgtctctttctctctctctctctct 22732
              23,680   23,700   23,720   23,740   23,760   23,780
KY962518_1   ctctttctgtctgtttctctctgcctgtctccgtctttctctgtctgcctctctctttcttttctgcgtctctctgtctctctctctctgtgcctatcttctgtcttactctgtttctctgcctgcctg 23719
human_rDNA   ctctttctgtctgtttctctctgcctgtctccgtctttctctgtctgcctctctctttcttttctgcgtctctctgtctctctctctctgtgcctatcttctgtcttactctgtttctctgcctgcctg 22862
              23,800   23,820   23,840   23,860   23,880   23,900   23,920
KY962518_1   tctgtctgtctgtctctctctctctctgtctctctctttctgtctgtttctctctgtctctctgtccatctctgtctttctctgtccgtctctctctttctccctgtctctgtctctgcctctgcctc 23849
human_rDNA   tctgtctgtctgtctctctctctctctgtctctctctttctgtctgtttctctctgtctctctgtccatctctgtctttctctgtccgtctctctctttctccctgtctctgtctctgcctctgcctc 22992
              23,940   23,960   23,980   24,000   24,020   24,040
KY962518_1   tctctctctgtctctctctttctatctgtttctctctgtctctctgtccatctctgtctttctctgtctgtctctctctttctccctgtctctgtctctgcctctctctctctctctctctgtctgtctc 23979
human_rDNA   tctctctctgtctctctctttctatctgtttctctctgtctctctgtccatctctgtctttctctgtctgtctctctctttctccctgtctctgtctctgcctctctctctctctctctctgtctgtctc 23122
              24,060   24,080   24,100   24,120   24,140   24,160   24,180
KY962518_1   tctcactgtgtgtgtgtctctgtctctgcctctctctctctctctctctctgtctgtctctctcactgtgtgtgtctgtcttctgtcttactctccttctctgcctgtccgtctgtctgtctgtctctcc 24109
human_rDNA   tctcactgtgtgtgtgtctctgtctctgcctctctctctctctctctctctgtctgtctctctcactgtgtgtgtctgtcttctgtcttactctccttctctgcctgtccgtctgtctgtctgtctctcc 23252
              24,200   24,220   24,240   24,260   24,280   24,300
KY962518_1   ctctctccctccctttctgtttctctctctctctctctctctttctgtctgtttctctctttctctctctgtctgtctctttctctgtctgtctgtctctctctttctttttctctgtctctctgtct 24239
human_rDNA   ctctctccctccctttctgtttctctctctctctctctctctttctgtctgtttctctctttctctctctgtctgtctctttctctgtctgtctgtctctctctttctttttctctgtctctctgtct 23382
              24,320   24,340   24,360   24,380   24,400   24,420   24,440
KY962518_1   ctctctgtgcctgtctctctgtctgtgcctatcttctgtcttactctctttctctggctgactgcctgtctctctctctctctctctctctctctctctctgcctgtctccgtccctccctccctgtctg 24369
human_rDNA   ctctctgtgcctgtctctctgtctgtgcctatcttctgtcttactctctttctctggctgactgcctgtctctctctctctctctctctctctctctctctgcctgtctccgtccctccctccctgtctg 23512
              24,460   24,480   24,500   24,520   24,540   24,560
KY962518_1   tctgtttctctctctgtccatttctgtctgtctctttctctttctctctctttctttctctctgtctctctctgtctctctctg--tctctctctctctctctctctctctctctctctctctctctctctg 24497
human_rDNA   tctgtttctctctctgtccatttctgtctgtctctttctctttctctctctttctttctctctgtctctctctgtctctctctgtctctctctctctctctctctctctctctctctctctctctctctctg 23642
              24,580   24,600   24,620   24,640   24,660   24,680   24,700
KY962518_1   cctttctctctcactgtgtcggtcttctgtcttactctctttctctgcctgcctctctgtctgtctgtctgtctgtctctctccctccatgtctctctctctctctctctcactcactgtctctccgtctctct 24627
human_rDNA   cctttctctctcactgtgtcggtcttctgtcttactctctttctctgcctgcctctctgtctgtctgtctgtctgtctctctccctccatgtctctctctctctctctctcactcactgtctctccgtctctct 23772
              24,720   24,740   24,760   24,780   24,800   24,820
KY962518_1   ctctttctgtctgtttctctctgtctctgtctttctgtgtgtctgtctgtctctctctctatttgtctttctccctccctgtctgttctctctctctctctctctctctctctctctctctctctctctgtct 24757
human_rDNA   ctctttctgtctgtttctctctgtctctgtctttctgtgtgtctgtctgtctctctctctatttgtctttctccctccctgtctgtt------tctctctctctctctctctctctctctctctccctgtct 23896
              24,840   24,860   24,880   24,900   24,920   24,940   24,960
KY962518_1   gtctgtttctctctatctctcgctgtccatctctgtctttctatgtctgtctctttctctgtcagtctgtcagacaccccgtgccgggtagggccctgccccttccacgaaagtgagaagcgcgtgctt 24887
human_rDNA   gtctgtttctctctatctctcgctgtccatctctgtctttctatgtctgtctctttctctgtcagtctgtcagacaccccgtgccgggtagggccctgccccttccacgaaagtgagaagcgcgtgctt 24026
```

```
                24,980                    25,000                    25,020                    25,040                    25,060                    25,080
KY962518_1  cggtgcttagagaggccgagaggaatctagacaggcgggccttgctgggcttccccactcggtgtacgatttcgggaggtcgaggccgggtccccgcttggatgcgaggggcattttcagacttttctct 25017
human_rDNA  cggtgcttagagaggccgagaggaatctagacaggcgggccttgctgggcttccccactcggtgtatgatttcgggaggtcgaggccgggtccccgcttggatgcgaggggcattttcagacttttctct 24156
        25,100                    25,120                    25,140                    25,160                    25,180                    25,200                    25,220
KY962518_1  cggtcacgtgtggcgtccgtacttctcctatttccccgataagctcctcgacttcaacataaacggcgtcctaagggtcgatttagtgtcatgcctctttcaccgccaccaccgaagatgaaagcaaaga 25147
human_rDNA  cggtcacgtgtggcgtccgtacttctcctatttccccgataagctcctcgacttcaacataaacggcgtcctaagggtcgatttagtgtcatgcctctttcaccgccaccaccgaagatgaaagcaaaga 24286
                25,240                    25,260                    25,280                    25,300                    25,320                    25,340
KY962518_1  tcggctaaataccgcgtgttctcatctagaagtgggaacttacagatgacagttcttgcatgggcagaacgaggggggaccggggacgcggaagcctgcttgagggaggaggggtggaaggagagacagct 25277
human_rDNA  tcggctaaataccgcgtgttctcatctagaagtgggaacttacagatgacagttcttgcatgggcagaacgaggggggaccggggacgcggaagcctgcttgagggaggaggggtggaaggagagacagct 24416
        25,360                    25,380                    25,400                    25,420                    25,440                    25,460                    25,480
KY962518_1  tcaggaagaaaacaaaacacgaatactgtcggacacagcactgactacccgggtgatgaaatcatctgcacactgaacaccccgtcacaagtttacctatgtcacagtcttgctcatgtatgcttgaac 25407
human_rDNA  tcaggaagaaaacaaaacacgaatactgtcggacacagcactgactacccgggtgatgaaatcatctgcacactgaacaccccgtcacaagtttacctatgtcacagtcttgctcatgtatgcttgaac 24546
                25,500                    25,520                    25,540                    25,560                    25,580                    25,600
KY962518_1  gacaaataaaagttc-gggggggagaagagaggagagagagagagagacggggagagaggggg-----------------gagaggggggggggagagagagagagagagagagagagagagagaaagagaag 25519
human_rDNA  gacaaataaaagttcggggggggagaagagaggagagagagagagagacggggagagagggggggagagggggggggggagagagagagagagagagagagagagagagagagagagagagagaaagagaag 24676
        25,620                    25,640                    25,660                    25,680                    25,700                    25,720                    25,740
KY962518_1  taaaaccaaccaccacctccttgacctgagtcaggggggtttctggcctttgggagaacgttcagcgacaatgcagtatttgggcccgttctttttttcttcttcttctttcttctttttttttggac 25649
human_rDNA  taaaaccaaccaccacctccttgacctgagtcaggggggtttctggcctttgggagaacgttcagcgacaatgcagtatttgggcccgttctttttttcttcttcttctttcttctttttttttggac 24806
                25,760                    25,780                    25,800                    25,820                    25,840                    25,860
KY962518_1  tgagtctctctcgctctgtcacccaggctgcggtgcggtggcgctctctcggctcactgaaacctctgcttcccgggttccagtgattcttcttcggtagctgggattacaggtgcgcaccatgacggcc 25779
human_rDNA  tgagtctctctcgctctgtcacccaggctgcggtgcggtggcgctctctcggctcactgaaacctctgcttcccgggttccagtgattcttcttcggtagctgggattacaggtgcgcaccatgacggcc 24936
        25,880                    25,900                    25,920                    25,940                    25,960                    25,980                    26,000
KY962518_1  ggctcatcgttctatttttagtagagacggggtttctccacgttggccacgctggtctcgaactcctgaccacaaatgatccaccttcctgggcctcccaaagtgctggaaacgacaggcctgagccgcc 25909
human_rDNA  ggctcatcgttctatttttagtagagacggggtttctccacgttggccacgctggtctcgaactcctgaccacaaatgatccaccttcctgggcctcccaaagtgctggaaacgacaggcctgagccgcc 25066
                26,020                    26,040                    26,060                    26,080                    26,100                    26,120
KY962518_1  gggatttcagcctttaaaagcgcgggccctgccacctttcgctgcggcccttacgctcagaatgacgtgtcctctctgccataggttgactccttgagtcccctaggccattgcactgtagcctgggcag 26039
human_rDNA  gggatttcagcctttaaaagcgcgggccctgccacctttcgctgcggcccttacgctcagaatgacgtgtcctctctgccataggttgactccttgagtcccctaggccattgcactgtagcctgggcag 25196
        26,140                    26,160                    26,180                    26,200                    26,220                    26,240                    26,260
KY962518_1  caagagccaaactccgtcccccacctccccgcgcacataataactaactaactaactaactaactaaaatctctacacgtcacccataagtgtgtgttcccgtgaggagtgatttctaagaaatggtac 26169
human_rDNA  caagagccaaactccgtcccccacctccccgcgcacataataactaactaactaactaactaactaaaatctctacacgtcacccataagtgtgtgttcccgtgaggagtgatttctaagaaatggtac 25326
                26,280                    26,300                    26,320                    26,340                    26,360                    26,380
KY962518_1  tgtacactgaacgcagtggctcacgtctgtcatcccgaggtcaggagttcgagaccagcccggccaacgtggtgaaaccccgtctctactgaaaatacgaaatggagtcaggcgccgtggggcaggcac 26299
human_rDNA  tgtacactgaacgcagtggctcacgtctgtcatcccgaggtcaggagttcgagaccagcccggccaacgtggtgaaaccccgtctctactgaaaatacgaaatggagtcaggcgccgtggggcaggcac 25456
        26,400                    26,420                    26,440                    26,460                    26,480                    26,500                    26,520
KY962518_1  ctgtaaccccagctactcgggaggctggggtggaagaattgcttgaacctggcaggcggaggctgcagtgacccaagatcgcaccactgcactacagcctgggcgacagagtgagacccggtctccagat 26429
human_rDNA  ctgtaaccccagctactcgggaggctggggtggaagaattgcttgaacctggcaggcggaggctgcagtgacccaagatcgcaccactgcactacagcctgggcgacagagtgagacccggtctccagat 25586
                26,540                    26,560                    26,580                    26,600                    26,620                    26,640
KY962518_1  aaatacgtacataaataaatacacacatacatacatacatacatacatacatacatacatacatacagatatacaagaaagaaaaaaagaaaagaaaagaaagagaaaatgaaagaaaaggcactgtatt 26559
human_rDNA  aaatacgtacataaataaatacacacatacatacatacatacatacatacatacatacatacatacagatatacaagaaagaaaaaaagaaaagaaaagaaagagaaaatgaaagaaaaggcactgtatt 25716
        26,660                    26,680                    26,700                    26,720                    26,740                    26,760                    26,780
KY962518_1  gctactgggctagggccttctctctgtctgtttctctctgttcgtctctgtctttctctctgtgtctctttctctgtctgtctgtctgtctgtctgtctctttctttctttctgtctctgtctttgtccc 26689
human_rDNA  gctactgggctagggccttctctctgtctgtttctctctgttcgtctctgtctttctctctgtgtctctttctctgtctgtctgtctgtctgtctgtctctttctttctttctgtctctgtctttgtccc 25846
                26,800                    26,820                    26,840                    26,860                    26,880                    26,900
KY962518_1  tctctccctctctgcctgtctcactgtgtctgtcttctatcttactctctttctctccccgtctgtctctctctcactccctccctgtctgtttctctctctctctctttctgtctgtttctgtctct 26819
human_rDNA  tctctccctctctgcctgtctcactgtgtctgtcttctatcttactctctttctctccccgtctgtctctctctcactccctccctgtctgtttctctctctctctctttctgtctgtttctgtctct 25976
        26,920                    26,940                    26,960                    26,980                    27,000                    27,020                    27,040
KY962518_1  ctctgtctgcctctctctttctctatctgtctctttctctgtctgtctgcccctctctttcttttttctgtgtctctctgtctgtctctctctctctctgtgcctatcttctgtcttactctctttctctg 26949
human_rDNA  ctctgtctgcctctctctttctctatctgtctctttctctgtctgtctgcccctctctttcttttttctgtgtctctctgtctgtctctctctctctctgtgcctatcttctgtcttactctctttctctg 26106
```

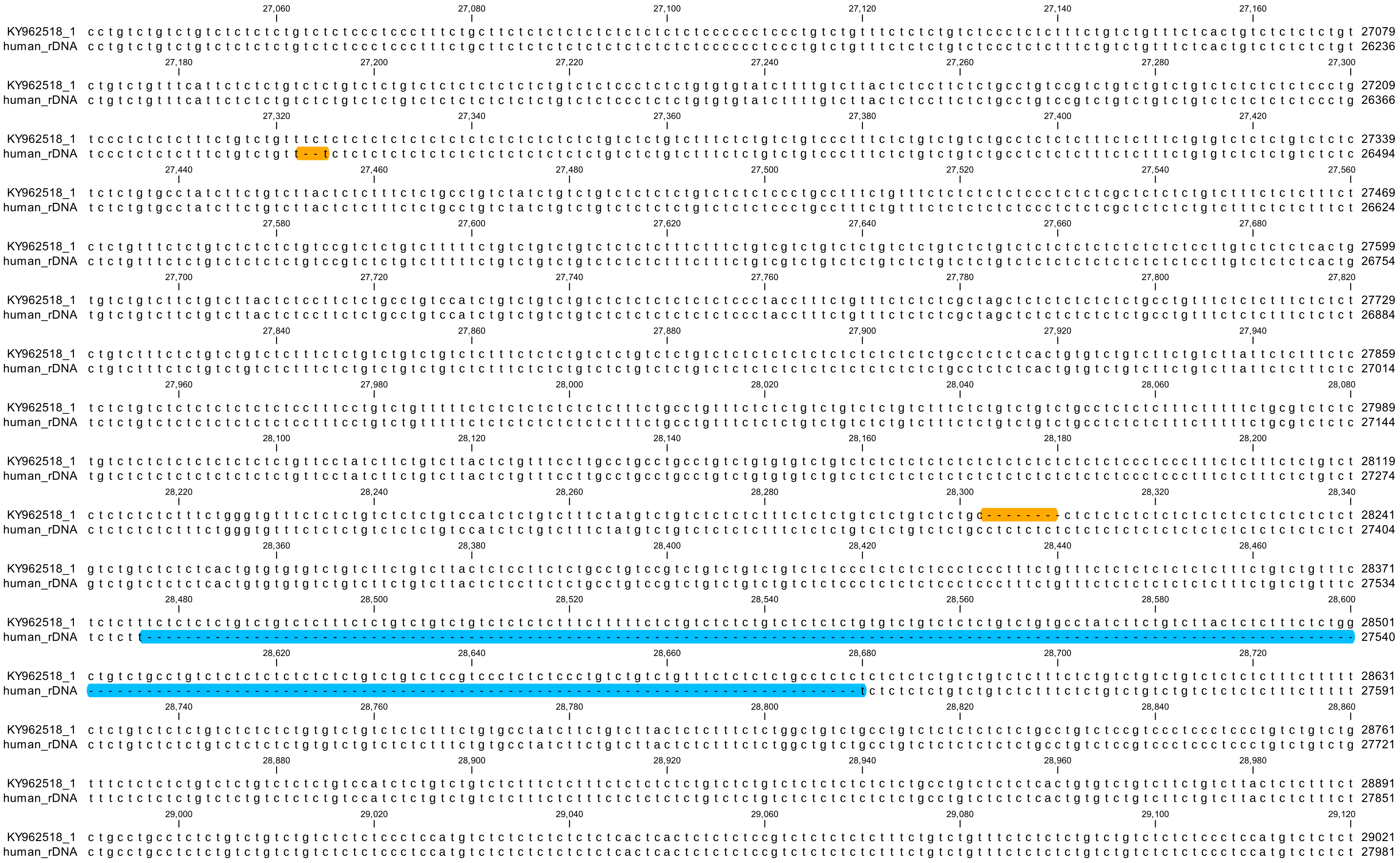
KY962518_1
human_rDNA

29,140 29,160 29,180 29,200 29,220 29,240

KY962518_1 c t c t c t c t c t c a c t c a c t c t c t c t c c g t c t c t c t c t c t c t t t c t g t c t g t t t c t c t c t c t g t c t g t c t c t c t c c c t c c a t g t c t c t c t c t c t c c c t c t c a c t c a c t c t c t c t c c g t c t c t c t c t c t t t 29151
human_rDNA c t c t c t c t c t c a c t c a c t c t c t c t c c g t c t c t c t c t c t c t t t c t g t c t g t t t c t c t c t c t g t c t g t c t c t c t c c c t c c a t g t c t c t c t c t c t c c c t c t c a c t c a c t c t c t c t c c g t c t c t c t c t c t t t 28111

29,260 29,280 29,300 29,320 29,340 29,360 29,380

KY962518_1 c t g t c t g t t t c t c t g t c t g t c t g t c t g t c t g t c t g - - - - - - - - t c t c t c t c t c t c t c t c t c t c t c t c t g t t t g t c t t t c t c c c t c c c t g t c t g t c t g t c t g t c t c t c t c t c t c t g t c t c t g t c t c t g t 29273
human_rDNA c t g t c t g t t t c t c t g t c t g t c t g t c t g t c t g t c t g t c t c t c t c t c t c t c t c t c t c t c t c t c t c t c t c t g t t t g t c t t t c t c c c t c c c t g t c t g t c t g t c t g t c t c t c t c t c t c t g t c t c t g t c t c t g t 28241

29,400 29,420 29,440 29,460 29,480 29,500

KY962518_1 c t c t c t c t c t t t c t c t t t c t g t c t g t t t c t c t c t a t c t c t c g c t g t c c a t c t c t g t c t t t c t a t g t c t g t c t c t t t c t c t g t c a g t c t g t c a g a c a c c c c c g t g c c g g g t a g g c c c t g c c c c t t c c a c g 29403
human_rDNA c t c t c t c t c t t t c t c t t t c t g t c t g t t t c t c t c t a t c t c t c g c t g t c c a t c t c t g t c t t t c t a t g t c t g t c t c t t t c t c t g t c a g t c t g t c a g a c a c c c c c g t g c c g g g t a g g c c c t g c c c c t t c c a c g 28371

29,520 29,540 29,560 29,580 29,600 29,620 29,640

KY962518_1 a g a g t g a g a a g c g c g t g c t t c g g t g c t t a g a g a g g c c g a g a g g a a t c t a g a c a g g c g g g c c t t g c t g g g c t t c c c c a c t c g g t g t a c g a t t t c g g g a g g t c g a g g c c g g g t c c c c g c t t g g a t g c g a g g g 29533
human_rDNA a g a g t g a g a a g c g c g t g c t t c g g t g c t t a g a g a g g c c g a g a g g a a t c t a g a c a g g c g g g c c t t g c t g g g c t t c c c c a c t c g g t g t a c g a t t t c g g g a g g t c g a g g c c g g g t c c c c g c t t g g a t g c g a g g g 28501

29,660 29,680 29,700 29,720 29,740 29,760

KY962518_1 g c a t t t t c a g a c t t t t c t c t c g g t c a c g t g t g g c g t c c g t a c t t c t c c t a t t t c c c c g a t a a g c t c c t c g a c t t c a a c a t a a a c t g t t a a g g c c g g a c g c a a c a c g g c g a a a c c c c g t c t c t a c t a a a a a 29663
human_rDNA g c a t t t t c a g a c t t t t c t c t c g g t c a c g t g t g g c g t c c g t a c t t c t c c t a t t t c c c c g a t a a g c t c c t c g a c t t c a a c a t a a a c t g t t a a g g c c g g a c g c a a c a c g g c g a a a c c c c g t c t c t a c t a a a a a 28631

29,780 29,800 29,820 29,840 29,860 29,880 29,900

KY962518_1 t a c a a a g c t g a g t c g g g a g c g g t g g g g c a g g c c c c t g t a a t g c c a g c t c c t c g g g a g g c t g a g g c g g g a g a a t c g c t t g a a c c a g g g a a g c g g a g g c t g c a g g g a g c c g a g a t c g c g c c a c t g c a c t a c g 29793
human_rDNA t a c a a a g c t g a g t c g g g a g c g g t g g g g c a g g c c c c t g t a a t g c c a g c t c c t c g g g a g g c t g a g g c g g g a g a a t c g c t t g a a c c a g g g a a g c g g a g g c t g c a g g g a g c c g a g a t c g c g c c a c t g c a c t a c g 28761

29,920 29,940 29,960 29,980 30,000 30,020

KY962518_1 g c c c a g g c t g t a g a g t g a g t g a g a c t c g g t c t c t a a a t a a a t a c g g a a a t t a a t t a a t t c a t t a a t t c t t t t c c c t g c t g a c g g a c a t t t g c a g g c a g g c a t c g g t t g t c t t c g g g c a t c a c c t a g c g g c 29923
human_rDNA g c c c a g g c t g t a g a g t g a g t g a g a c t c g g t c t c t a a a t a a a t a c g g a a a t t a a t t a a t t c a t t a a t t c t t t t c c c t g c t g a c g g a c a t t t g c a g g c a g g c a t c g g t t g t c t t c g g g c a t c a c c t a g c g g c 28891

30,040 30,060 30,080 30,100 30,120 30,140 30,160

KY962518_1 c a c t g t t a t t g a a a g t c g a c g t g a c a c g g a g g g a g g t c t c g c c g a c t t c a c c g a g c c t g g g g c a a c g g g t t t c t c t c t c t c c c t t c t g g a g g c c c c t c c c t c t c t c c c t c g t t g c c t a g g g a a c c t c g c c 30053
human_rDNA c a c t g t t a t t g a a a g t c g a c g t g a c a c g g a g g g a g g t c t c g c c g a c t t c a c c g a g c c t g g g g c a a c g g g t t t c t c t c t c t c c c t t c t g g a g g c c c c t c c c t c t c t c c c t c g t t g c c t a g g g a a c c t c g c c 29021

30,180 30,200 30,220 30,240 30,260 30,280

KY962518_1 t a g g g a a c c t c c g c c c t g g c g g g g g c c c t a t t g t t c t t t g a t c g g c g c t t t a c t t t t c t t t g t g t t t t g g c g c c t a g a c t c t t c t a c t t g g g c t t t g g g a a g g g t c a g t t t a a t t t t c a a g t t g c c c c c 30183
human_rDNA t a g g g a a c c t c c g c c c t g g c g g g g g c c c t a t t g t t c t t t g a t c g g c g c t t t a c t t t t c t t t g t g t t t t g g c g c c t a g a c t c t t c t a c t t g g g c t t t g g g a a g g g t c a g t t t a a t t t t c a a g t t g c c c c c 29151

30,300 30,320 30,340 30,360 30,380 30,400 30,420

KY962518_1 g g c t c c c c c c a c t a c c c a c g t c c c t t c a c c t t a a t t t a g t g a g t c g g t t a g g t g g g t t t c c c c c a a a c c g c c c c c c c c c c c c c c g c c t c c c a a c a c c c t g c t t g g a a a c c t t c c a g a g c c a c c c c g g t g t 30313
human_rDNA g g c t c c c c c c a c t a c c c a c g t c c c t t c a c c t t a a t t t a g t g a g t c g g t t a g g t g g g t t t c c c c c a a a c c g - c c c c c c c c c c c c c g c c t c c c a a c a c c c t g c t t g g a a a c c t t c c a g a g c c a c c c c g g t g t 29280

30,440 30,460 30,480 30,500 30,520 30,540

KY962518_1 g c c t c c g t c t t c t c t c c c c t t c c c c c a c c c c t t g c c g g c g a t c t c a t t c t t g c c a g g c t g a c a t t t g c a t c g g t g g g c g t c a g g c c t c a c t c g g g g g c c a c c g t t t t t g a a g a t g g g g g c g g c a c g g t c c 30443
human_rDNA g c c t c c g t c t t c t c t c c c c t t c c c c c a c c c c t t g c c g g c g a t c t c a t t c t t g c c a g g c t g a c a t t t g c a t c g g t g g g c g t c a g g c c t c a c t c g g g g g c c a c c g t t t t t g a a g a t g g g g g c g g c a c g g t c c 29410

30,560 30,580 30,600 30,620 30,640 30,660 30,680

KY962518_1 c a c t t c c c c g g a g g c a g c t t g g g c c g a t g g c a t a g c c c c t t g a c c c g c g t g g g c a a g c g g g c g g g t c t g c a g t t g t g a g g c t t t t c c c c c c g c t g c t t c c c g c c t c a g g c c t c c c t c c c t a g g a a a g c t t 30573
human_rDNA c a c t t c c c c g g a g g c a g c t t g g g c c g a t g g c a t a g c c c c t t g a c c c g c g t g g g c a a g c g g g c g g g t c t g c a g t t g t g a g g c t t t t c c c c c c g c t g c t t c c c g c c t c a g g c c t c c c t c c c t a g g a a a g c t t 29540

30,700 30,720 30,740 30,760 30,780 30,800

KY962518_1 c a c c c t g g c t g g g t c t c g g t c a c c t t t t a t c a c g a t g t t t t a g t t t c t c c g c c c t c c g g c c a g c a g a g t t t c a c a a t g c g a a g g g c g c c a c g g c t c t a g t c t g g g c c t t c t c a g t a c t t g c c c a a a a t a g 30703
human_rDNA c a c c c t g g c t g g g t c t c g g t c a c c t t t t a t c a c g a t g t t t t a g t t t c t c c g c c c t c c g g c c a g c a g a g t t t c a c a a t g c g a a g g g c g c c a c g g c t c t a g t c t g g g c c t t c t c a g t a c t t g c c c a a a a t a g 29670

30,820 30,840 30,860 30,880 30,900 30,920 30,940

KY962518_1 a a a c g c t t t c t g a a a a c t a a t a a c t t t g c t c a c t t a a g a t t t c c a g g g a c g g c g c c t t g g c c c g t g t t t g t t g g c t t g t t t t g t t t c g t t c t g t t t t g t t t t g t t c g t g t t t t t c c t t t c t c g t a t g t c t 30833
human_rDNA a a a c g c t t t c t g a a a a c t a a t a a c t t t g c t c a c t t a a g a t t t c c a g g g a c g g c g c c t t g g c c c g t g t t t g t t g g c t t g t t t t g t t t c g t t c t g t t t t g t t t t g t t c g t g t t t t t c c t t t c t c g t a t g t c t 29800

30,960 30,980 31,000 31,020 31,040 31,060

KY962518_1 t t c t t t t c a g g t g a a g t a g a a a t c c c c a g t t t t c a g g a a g a c g t c t a t t t t c c c c a a g a c a c g t t a g c t g c c g t t t t t t c c t g t t g t g a a c t a g c g c t t t t g t g a c t c t c t c a a c g t g c a g t g a g a g c c g 30963
human_rDNA t t c t t t t c a g g t g a a g t a g a a a t c c c c a g t t t t c a g g a a g a c g t c t a t t t t c c c c a a g a c a c g t t a g c t g c c g t t t t t t c c t g t t g t g a a c t a g c g c t t t t g t g a c t c t c t c a a c g t g c a g t g a g a g c c g 29930

31,080 31,100 31,120 31,140 31,160 31,180 31,200

KY962518_1 g t t g a t g t t t a c t a t c c t t c a t c a t g a c a t c t t a t t t t c t a g a a a t c c g t a g g c g a a t g c t g c t g c t g c t c t t g t t g c t g t t g t t g t t g t t g t t g t t g t c g t c g t t g c t g t t g t c g t t g t c g t t g t t g t t 31093
human_rDNA g t t g a t g t t t a c t a t c c t t c a t c a t g a c a t c t t a t t t t c t a g a a a t c c g t a g g c g a a t g c t g c t g c t g c t c t t g t t g c t g t t g t t g t t g t t g t t g t t g t c g t c g t t g c t g t t g t c g t t g t c g t t g t t g t t 30060

```
            31,220  31,240  31,260  31,280  31,300  31,320
KY962518_1  gtcgttgtcgttgttttcaaagtataccccggccaccgtttatgggatcaaaagcattataaaatatgtgtgattatttcttgagcacgcccttcctccccctctctctgtctctctgtctgtctctgtc 31223
human_rDNA  gtcgttgtcgttgttttcaaagtataccccggccaccgtttatgggatcaaaagcattataaaatatgtgtgattatttcttgagcacgcccttcctccccctctctctgtctctctgtctgtctctgtc 30190

            31,340  31,360  31,380  31,400  31,420  31,440  31,460
KY962518_1  tctctctttctctgtctgtcttctctctctctctctctgtgtctctctctctctgcctgtctgtttctctctctctctgc--ctctctctctctctctctctctgcctgtctctctcactgtgtctgtct 31351
human_rDNA  tctctctttctctgtctgtcttctctctctctctctctgtgtctctctctctctgcctgtctgtttctctctctctctgcctctctctctctctctctctctctgcctgtctctctcactgtgtctgtct 30320

            31,480  31,500  31,520  31,540  31,560  31,580
KY962518_1  tctgtcttactccctttctctgtctgtctgtcggtctctctctctctctctccctgtctgtatgtttctctctgtctctgtctctctctctctttctgtttctctctctccgtctctgtctttctctgac 31481
human_rDNA  tctgtcttactccctttctctgtctgtctgtcggtctctctctctctctctccctgtctgtatgtttctctctgtctctgtctctctctctctttctgtttctctctctccgtctctgtctttctctgac 30450

            31,600  31,620  31,640  31,660  31,680  31,700  31,720
KY962518_1  tgtctctctctttccttctctctgtctctctctgcctgtctctctcactctgtcttctgtcttactctctctctctgcctgcctgtctctctcactctctctctgtgtgtctctctctctctctttctgt 31611
human_rDNA  tgtctctctctttccttctctctgtctctctctgcctgtctctctcactctgtcttctgtcttactctctctctctgcctgcctgtctctctcactctctctctgtgtgtctctctctctctctttctgt 30580

            31,740  31,760  31,780  31,800  31,820  31,840
KY962518_1  ttctctctgtctctctgtccgtctctgtctttctctgtctgtctctttgtctgtctgtctttgtctttccttctctctgtctctgtctctctcactgtgtctgtcttctgtcttagtctctctctctctc 31741
human_rDNA  ttctctctgtctctctgtccgtctctgtctttctctgtctgtctctttgtctgtctgtctttgtctttccttctctctgtctctgtctctctcactgtgtctgtcttctgtcttagtctctctctctctc 30710

            31,860  31,880  31,900  31,920  31,940  31,960  31,980
KY962518_1  tctccctgtctgtctgtctctctctctctccccctgtctgtt------------tctctctctctctctctctctgtctttgtctttctttctgtctctgtctctctctctctctctgtgtgtctgtc 31859
human_rDNA  tctccctgtctgtctgtctctctctctctccccctgtctgtttctctctctctctctctctctctctctctctctctgtctttgtctttctttctgtctctgtctctctctctctctctgtgtgtctgtc 30840

            32,000  32,020  32,040  32,060  32,080  32,100
KY962518_1  ttctgtcttactgtctttctctgcctgtctgtctgtctgtctctctctgtctgtctctctctctctctcccccctgtcggctgtttctctgtctctgtctgtgtctctctttctgtctgtttctctctgt 31989
human_rDNA  ttctgtcttactgtctttctctgcctgtctgtctgtctgtctctctctgtctgtctctctctctctctcccccc-gtcggctgtttctctgtctctgtctgtgtctctctttctgtctgtttctctctgt 30969

            32,120  32,140  32,160  32,180  32,200  32,220  32,240
KY962518_1  ctgtctttctctctctgtctctttctctctgtctctctgtctgtctctgtctctctctctgtctctctctctctgtgggggtgtgtgtgtgtgtgtg------tatgtgtgtgtgtgtgtgtgtgtgt 32113
human_rDNA  ctgtctttctctctctgtctctttctctctgtctctctgtctgtctctgtctctctctctgtctctctctctctgtgggggtgtgtgtgtgtgtgtgtgtgtatgtgtgtgtgtgtgtgtgtgtgtgt 31099

            32,260  32,280  32,300  32,320  32,340  32,360
KY962518_1  ctgccttctgtcttactctctttctctgcctgtctgtctgcctgtctgtttgtctctctctctctctgcctgtctctcccttcctgtctgtttctctctctttctgtttctctctgtctctgtccatctc 32243
human_rDNA  ctgccttctgtcttactctctttctctgcctgtctgtctgcctgtctgtttgtctctctctctctctgcctgtctctcccttcctgtctgtttctctctctttctgtttctctctgtctctgtccatctc 31229

            32,380  32,400  32,420  32,440  32,460  32,480  32,500
KY962518_1  tgtctttctccgtctgtctctttatctgtctctccgtctgtctctttatctgtctctctctctctctctttctgtctttctctctctgtgtatcgttgtctctctctgtctgtctctgtctctgtctctctg 32373
human_rDNA  tgtctttctccgtctgtctctttatctgtctctccgtctgtctctttatctgtctctctctctctctctttctgtctttctctctctgtgtatcgttgtctctctctgtctgtctctgtctctgtctctctg 31359

            32,520  32,540  32,560  32,580  32,600  32,620
KY962518_1  --tctctctctctctctctctctctgtctgtctgtccgtctgtctgtctcggtctctggctctcgctatcccccgccctctctttttttgcaaaagaagctcaagtacatctaatctaatcccttacc 32501
human_rDNA  tctctctctctctctctctctctctgtctgtctgtccgtctgtctgtctcggtctctggctctcgctatctcccgccctctctttttttgcaaaagaagctcaagtacatctaatctaatcccttacc 31489

            32,640  32,660  32,680  32,700  32,720  32,740  32,760
KY962518_1  aaggcctgaattcttcacttctgacatcccagatttgatctccctacagaatgctgtacagaactggcgagttgatttctggacttggatacctcatagaaactacatatgaataaagatccaatcctaa 32631
human_rDNA  aaggcctgaattcttcacttctgacatcccagatttgatctccctacagaatgctgtacagaactggcgagttgatttctggacttggatacctcatagaaactacatatgaataaagatccaatcctaa 31619

            32,780  32,800  32,820  32,840  32,860  32,880
KY962518_1  aatctggggtggcttctccctcgactgtctcgaaaaatcgtacctctgttcccctaggatgccggaagagttttctcaatgtgcatctgcccgtgtcctaagtgatctgtgaccgagccctgtccgtcct 32761
human_rDNA  aatctggggtggcttctccctcgactgtctcgaaaaatcgtacctctgttcccctaggatgccggaagagttttctcaatgtgcatctgcccgtgtcctaagtgatctgtgaccgagccctgtccgtcct 31749

            32,900  32,920  32,940  32,960  32,980  33,000  33,020
KY962518_1  gtctcaaatatgtacgtgcaaacacttctctccatttccacaactacccacggccccttgtggaaccactggctctttgaaaaaaatcccagaagtggttttggctttttggctaggaggcctaagcctg 32891
human_rDNA  gtctcaaatatgtacgtgcaaacacttctctccatttccacaactacccacggccccttgtggaaccactggctctttgaaaaaaatcccagaagtggttttggctttttggctaggaggcctaagcctg 31879

            33,040  33,060  33,080  33,100  33,120  33,140
KY962518_1  ctgagaactttcctgcccaggatcctgtgtgaccaaaagtgcctctgctgggagctgggatcctcgggaccatgcttgctagcgctggatgagtctctggaaggacgcacgggactccgcaaagctgacc 33021
human_rDNA  ctgagaactttcctgcccaggatcctgtgtgaccaaaagtgcctctgctgggagctgggatcctcgggaccatgcttgctagcgctggatgagtctctggaaggacgcacgggactccgcaaagctgacc 32009

            33,160  33,180  33,200  33,220  33,240  33,260  33,280
KY962518_1  tgtcccaccgaggtcaaatggatacctctgcattggcccgaggcctccgaagtacatcaccgtcaccaaccgtcaccgtcagcatccttgtgagcctgcccaaggccccgcctccggggagactcttggg 33151
human_rDNA  tgtcccaccgaggtcaaatggatacctctgcattggcccgaggcctccgaagtacatcaccgtcaccaaccgtcaccgtcagcatccttgtgagcctgcccaaggccccgcctccggggagactcttggg 32139
```

```
                33,300              33,320              33,340              33,360              33,380              33,400
KY962518_1  agcccggccttcgtcggctaaagtccaaagggatggtgacttccacccacaaggtccccactgaacggcgaagatgtggagcgtaggtcagagaggggaccaggaggggagacgtcccgacaggcgacga 33281
human_rDNA  agcccggccttcgtcggctaaagtccaaagggatggtgacttccacccacaaggtccccactgaacggcgaagatgtggagcgtaggtcagagaggggaccaggaggggagacgtcccgacaggcgacga 32269
      33,420              33,440              33,460              33,480              33,500              33,520              33,540
KY962518_1  gttcccaaggctctggccacccccacccacgccccacgccccacgtcccgggcacccgcgggacaccgccgctttatcccctcctctgtccacagccggcccccacccccaccacgcaacccacgcacacacg 33411
human_rDNA  gttcccaaggctctggccacccccacccacgccccacgccccacgtcccgggcacccgcgggacaccgccgctttatcccctcctctgtccacagccggcccccacccccaccacgcaacccacgcacacacg 32399
                33,560              33,580              33,600              33,620              33,640              33,660
KY962518_1  ctggaggttccaaaaccacacggtgtgactagagcctgacggagcgagagcccatttcacgaggtgggagggggtgggggtggggtgggttgggggttgtggggtctgtggcgagcccgattctccctctt 33541
human_rDNA  ctggaggttccaaaaccacacggtgtgactagagcctgacggagcgagagcccatttcacgaggtgggagggggtgggggtggggtgggttgggggttgtggggtctgtggcgagcccgattctccctctt 32529
      33,680              33,700              33,720              33,740              33,760              33,780              33,800
KY962518_1  gggtggctacaggctagaaatgaatatcgcttcttgggcggagggggcttccttaggccatcaccgcttgcgggactacctctcaaaccctcccttgaggccacaaaatagattccaccccacccatcgac 33671
human_rDNA  gggtggctacaggctagaaatgaatatcgcttcttgggcggagggggcttccttaggccatcaccgcttgcgggactacctctcaaaccctcccttgaggccacaaaatagattccaccccacccatcgac 32659
                33,820              33,840              33,860              33,880              33,900              33,920
KY962518_1  gtttccccgggtgctggatgtatcctgtcaagagacctgagcctgacaccgtcgaattaaacaccttgactggctttgtgtgtttgtttgtttctgagatggagtcttgctctgtcccccaggctggag 33801
human_rDNA  gtttccccgggtgctggatgtatcctgtcaagagacctgagcctgacaccgtcgaattaaacaccttgactggctttgtgtgtttgtttgtttctgagatggagtcttgctctgtcccccaggctggag 32789
      33,940              33,960              33,980              34,000              34,020              34,040              34,060
KY962518_1  tgcagtggcgtgatctcagctcactggaacctctgcctcctgggttcaagtgattctcctgtctcagcgccaccatggccggctcatttttttttttttttttttttttttttttttttttttttttttt 33931
human_rDNA  tgcagtggcgtgatctcagctcactggaacctctgcctcctgggttcaagtgattctcctgtctcagcgccaccatggccggctca--------------------------tttttttttttttttttt 32893
                34,080              34,100              34,120              34,140              34,160              34,180
KY962518_1  ttggtagacacggggtttcaccctctttcattggttttcactggagattctagattcgagccacacctcattccgtgccacagagagacttc-ttttttttttttttttttaagcgcaacgcaacatgtc 34060
human_rDNA  ttggtagacacggggtttcaccctctttcattggttttcactggagattctagattcgagccacacctcattccgtgccacagagagacttctttttttttttttttttttaagcgcaacgcaacatgtc 33023
      34,200              34,220              34,240              34,260              34,280              34,300              34,320
KY962518_1  tgccttatttgagtggcttcctatatcattataattgtgttatagatgaagaaacggtattaaacactgtgctaatgatagtgaaagtgaagacaaaagaaaggctatctatttttgtggttagaataaag 34190
human_rDNA  tgccttatttgagtggcttcctatatcattataattgtgttatagatgaagaaacggtattaaacactgtgctaatgatagtgaaagtgaagacaaaagaaaggctatctatttttgtggttagaataaag 33153
                34,340              34,360              34,380              34,400              34,420              34,440
KY962518_1  ttgctcagtatttagagctacctaaatacgtcagcatttacactcttcctagtaaaagctggccaatctgaataatcctcctttaaacaaacacaatttttgataggttaagatttttttaagaatgcg 34320
human_rDNA  ttgctcagtatttagagctacctaaatacgtcagcatttacactcttcctagtaaaagctggccgatctgaataatcctcctttaaacaaacacaatttttgataggttaagatttttttaagaatgcg 33283
      34,460              34,480              34,500              34,520              34,540              34,560              34,580
KY962518_1  actcctgcaaaatagctgaacagacgatacacatttaaaaaaataacaacacaaggatcaaccagacttgggaaaaaatcgaaaaccacacaagtcttatgaagaactgagttcttaaaataggacggag 34450
human_rDNA  actcctgcaaaatagctgaacagacgatacacatttaaaaaaataacaacacaaggatcaaccagacttgggaaaaaatcgaaaaccacacaagtcttatgaagaactgagttcttaaaataggacggag 33413
                34,600              34,620              34,640              34,660              34,680              34,700
KY962518_1  aacgtagctatcggaagagaaggcagtattggcaagttgattgttacgttggtcagcagtagctggcactatctttttggccatctttcgggcaatgtaactactacagcaaaatgagatatgatccatt 34580
human_rDNA  aacgtagctatcggaagagaaggcagtattggcaagttgattgttacgttggtcagcagtagctggcactatctttttggccatctttcgggcaatgtaactactacagcaaaatgagatatgatccatt 33543
      34,720              34,740              34,760              34,780              34,800              34,820              34,840
KY962518_1  aaacaacatattcgcaaatcaaaaagtgtttcagtaatataatgcttcagatttagaagcaaatcaaatgatagaactccactgctgtaataagtcaccccaaagatcaccgtatctgacaaaataacta 34710
human_rDNA  aaacaacatattcgcaaatcaaaaagtgtttcagtaatataatgcttcagatttagaagcaaatcaaatgatagaactccactgctgtaataagtcaccccaaagatcaccgtatctgacaaaataacta 33673
                34,860              34,880              34,900              34,920              34,940              34,960
KY962518_1  ccacagggttatgacttcagaatcatactttctcttgatatttacttatgtatgtatttatttttttttaatttatttctcttgagacggcgtctcgctctgtcgcccaggctggagtgcgatggtgtgat 34840
human_rDNA  ccacagggttatgacttcagaatcatactttctcttgatatttacttatgtatgtatttatttttttttaatttatttctcttgagacggcgtctcgctctgtcgcccaggctggagtgcgatggtgtgat 33803
      34,980              35,000              35,020              35,040              35,060              35,080              35,100
KY962518_1  ctcggctcactgcaaccgccacctccctgggttcaagcgattctcctgcctcagcctcccgagtagctgggactacaggtgcccgccaccacgcccagctaatctttatacttttaatagagacggggtt 34970
human_rDNA  ctcggctcactgcaaccgccacctccctgggttcaagcgattctcctgcctcagcctcccgagtagctgggactacaggtgcccgccaccacgcccagctaatctttatacttttaatagagacggggtt 33933
                35,120              35,140              35,160              35,180              35,200              35,220
KY962518_1  tcaccgtgtcggcccggatggtctcgatctcttgacctcgtgacccgcccgcctcggcctcccaaagtgctgggatgacaggcgtgagccactgagcccggccttctcttgacgtttaaactatgaagtc 35100
human_rDNA  tcaccgtgtcggcccggatggtctcgatctcttgacctcgtgacccgcccgcctcggcctcccaaagtgctgggatgacaggcgtgagccactgagcccggccttctcttgacgtttaaactatgaagtc 34063
      35,240              35,260              35,280              35,300              35,320              35,340              35,360
KY962518_1  agtccagagaaacgcaataaatgtcaacggtgaggatggtgttgaggcagaagtaggaccacacttttttcctatcttattcagttgataacaatatgacctaggtagtaatttcctatgtgcctacttat 35230
human_rDNA  agtccagagaaacgcaataaatgtcaacggtgaggatggtgttgaggcagaagtaggaccacacttttttcctatcttattcagttgataacaatatgacctaggtagtaatttcctatgtgcctacttat 34193
```

```
                35,380  35,400  35,420  35,440  35,460  35,480
KY962518_1  acacgagtacaaaagagtaaaacagagagactgctaaattaaagggtacgtgaagttcttcatagtaactccgtaaactggaacactgtcaaaaagcagcagctagtgaattgtttccatgtatttttct 35360
human_rDNA  acacgagtacaaaagagtaaaacagagagactgctaaattaaagggtacgtgaagttcttcatagtaactccgtaaactggaacactgtcaaaaagcagcagctagtgaattgtttccatgtatttttct 34323
                35,500  35,520  35,540  35,560  35,580  35,600  35,620
KY962518_1  attatccaataagtgaactatgctattcctttccagtctcccaagcacttcttgtccccatcaccacttcggtgctcgaagaaaaagtaacaaatcaaggaacacaactaaagaaacacacacacaaacc 35490
human_rDNA  attatccaataagtgaactatgctattcctttccagtctcccaagcacttcttgtccccatcaccacttcggtgctcgaagaaaaagtaacaaatcaaggaacacaactaaagaaacacacacacaaacc 34453
                35,640  35,660  35,680  35,700  35,720  35,740
KY962518_1  aaagacaactacagcgtctgcaaaagtttgctagaagactgaaactgttgagtataaggatctggtattctacgatcatgagttcacttcagagtttgttcaagacatacgtttcgtaaggaaacatctt 35620
human_rDNA  aaagacaactacagcgtctgcaaaagtttgctagaagactgaaactgttgagtataaggatctggtattctacgatcatgagttcacttcagagtttgttcaagacatacgtttcgtaaggaaacatctt 34583
                35,760  35,780  35,800  35,820  35,840  35,860  35,880
KY962518_1  agttagaagttattcagcagtaggtaccatccctaagtatttttcaccaaattcgtgacaataaagagctatctaaccagaaaaattagcgagtaccggcaccatccatagggctttgtctttacgcttc 35750
human_rDNA  agttagaagttattcagcagtaggtaccatccctaagtatttttcaccaaattcgtgacaataaagagctatctaaccagaaaaattagcgagtaccggcaccatccatagggctttgtctttacgcttc 34713
                35,900  35,920  35,940  35,960  35,980  36,000
KY962518_1  attagcacttaccatgccttacaatgtctaggattgaccctgatagcatttcgaaaacaagctaatgctttgtccagttcttcagtgaagacaagctcacgccctaatgcgctataggcataagcatcat 35880
human_rDNA  attagcacttaccatgcctcacaatgtctaggattgaccctgatagcatttcgaaaacaagctaatgctttgtccagttcttcagtgaagacaagctcacgccctaatgcgctataggcataagcatcat 34843
                36,020  36,040  36,060  36,080  36,100  36,120  36,140
KY962518_1  ttggatccacttcgagagttctctggaagaattgaatcgcaatatcgtgttcccgttgcagaccgaaacagttccctgcagcacaccaggcctctggctggcgaatttttatccatgtctgtgaagtctt 36010
human_rDNA  ttggatccacttcgagagttctctggaagaattgaatcgcaatatcgtgttcccgttgcagaccgaaacagttccctgcagcacaccaggcctctggctggcgaatttttatccatgtctgtgaagtctt 34973
                36,160  36,180  36,200  36,220  36,240  36,260
KY962518_1  tggacagaactgaaagagcaacctctttcggaggatgccaaagtgttgtagagtagatctccatgccttcgactctgtaattctcaatcctcctaacctctgagaattgtctttcagcttgcgtggactc 36140
human_rDNA  tggacagaactgaaagagcaacctctttcggaggatgccaaagtgttgtagagtagatctccatgccttcgactctgtaattctcaatcctcctaacctctgagaattgtctttcagcttgcgtggactc 35103
                36,280  36,300  36,320  36,340  36,360  36,380  36,400
KY962518_1  tgaaagtttacaataggcccttccgatttggcacagtacccaaccggtattgcagtggtgagaagctagatggctcaagatgctgatagcttctttgccgtggtaagaacacaaagctaaataacctttc 36270
human_rDNA  tgaaagtttacaataggcccttccgatttggcacagtacccaaccggtattgcagtggtgagaagctagatggctcaagatgctgatagcttctttgccgtggtaagaacacaaagctaaataacctttc 35233
                36,420  36,440  36,460  36,480  36,500  36,520
KY962518_1  cccctttcacgaagaaggctcatcaagccttccgctgctgctttttgtagattaaaagcctgaatctgaggcgcgattgtggctattttcccttctgaaatgacggaagagtccaattttgtcacttcca 36400
human_rDNA  cccctttcacgaagaaggctcatcaagccttccgctgctgctttttgtagattaaaagcctgaatctgaggcgcgattgcggctatttccccttctgaaatgacggaagagtccaattttgtcacttcca 35363
                36,540  36,560  36,580  36,600  36,620  36,640  36,660
KY962518_1  ggctatcacttatgttcggtggagttattgctcctttattagttttactttggttcttctgtttgggatttaggtggaaacttcattttaatttctcctattctcctcggttgtggagctgtcact 36530
human_rDNA  ggctatcacttatgttcggtggagttattgctcctttattagttttactttggttcttctgtttgggatttaggtggaaacttcattttaatttctcctattctcctcggttgtggagctgtcact 35493
                36,680  36,700  36,720  36,740  36,760  36,780
KY962518_1  agtcaagagtcgtgaatttcttcgaggcggtgcatttggggagatgccatagtggggctcaatacctgaggtgttgcccttgtcggcggaccagaactttgtgtttttgcaaggactggagttaccttt 36660
human_rDNA  agtcaagagtcgtgaatttcttcgaggcggtgcatttggggagatgccatagtggggctcaatacctgaggtgttgcccttgtcggcggaccagaactttgtgtttttgcaaggactggagttaccttt 35623
                36,800  36,820  36,840  36,860  36,880  36,900  36,920
KY962518_1  cggctctttcccctctgcgagaagacagacggtgttccggtttggccgattctggcaacaggctttttttgaaggggctccggtggatggcacgtcaatgacagacggtgtctcataccagtgcagttttg 36790
human_rDNA  cggctctttcccctctgcgagaagacagacggtgttccggtttggccgattctggcaacaggctttttttgaaggggctccggtggatggcacgtcaatgacagacggtgtctcataccagtgcagttttg 35753
                36,940  36,960  36,980  37,000  37,020  37,040
KY962518_1  tcaatagggtccgtctccgggacttggggtttctaatggcaaaatgccaacacttggggttaatggactaacagctgctggtcctcctaataaacttcgaccagtttttggtttatgttgaacctgttta 36920
human_rDNA  tcaatagggtccgtctccgggacttggggtttctaatggcaaaatgccaacgcttggggttaatggactaacagctgctggtcctcctaataaacttcgaccagtttttggtttatgttgaacctgttta 35883
                37,060  37,080  37,100  37,120  37,140  37,160  37,180
KY962518_1  gatcatatggaagttcctgttcccagtgggacagtatcaggtgaaaggacagctgaatcgatagaagacactggggagtctgtattcaaggagtactttgaattggaagattctaaattccatccgtttc 37050
human_rDNA  gatcatatggaagttcctgttcccagtgggacagtatcaggtgaaaggacagctgaatcgatagaagacactggggagtctgtattcaaggagtactttgaattggaagattctaaattccatccgtttc 36013
                37,200  37,220  37,240  37,260  37,280  37,300
KY962518_1  attcgacggtgtcctggggtgtttccgtaagaacggtctcgggctgtctgtgacataaactaggacgaggtccaagtgttgtggcgcaacacttggacaggcagttgctaaagctctctagagaggtgaa 37180
human_rDNA  attcgacggtgtcctggggtgtttccgtaagaacggtctcgggctgtctgtgacataaactaggacgaggtccaagtgttgtggcgcaacacttggacaggcagttgctaaagctctctagagaggtgaa 36143
                37,320  37,340  37,360  37,380  37,400  37,420  37,440
KY962518_1  tcaaaatgtttggtcaggatctggcttttccccccctatttcacatcatgattcaaagggacaccagaggaaaggatttcaacgaaggctcttttggtcacattctgatcctttggtaagccgatctgtct 37310
human_rDNA  tcaaaatgtttggtcaggatctggcttttccccccctatttcacatcatgattcaaagggacaccagaggaaaggatttcaacgaaggctcttttggtcacattctgatcctttggtaagccgatctgtct 36273
```

37,460 37,480 37,500 37,520 37,540 37,560
KY962518_1 tgcaatatacatgtcccgacgatggaaggggaaagcgagctgaatcaccaaactcaggaacgataatatcatcgtggcttttctgcttatgaaacactccacccgataagatttgatccccttctgcaag 37440
human_rDNA tgcaatatacatgtcccgacgatggaaggggaaagcgagctgaatcaccaaactcaggaacgataatatcatcgtggcttttctgcttatgaaacactccacccgataagatttgatccccttctgcaag 36403

37,580 37,600 37,620 37,640 37,660 37,680 37,700
KY962518_1 cttgctgagatcaacacaacatttcgcaagcaggcatttgcattgcggggtagtacaactgtgtcctttcaagagtctatatgttttataggcctttcctgagcggtaagaacaggtcgccagtaagaac 37570
human_rDNA cttgctgagatcaacacaacatttcgcaagcaggcatttgcattgcggggtagtacaactgtgtcctttcaagagtctatatgttttataggcctttcctgagcggtaagaacaggtcgccagtaagaac 36533

37,720 37,740 37,760 37,780 37,800 37,820
KY962518_1 aaggcttcttctgagtgtacttctgcataaaggcgttctgcgggggaaaccgcatctcggtaggcatagtggtttagtgcttgccatatagcagcctggacgggtccctgcagcaccgccatcctcgagg 37700
human_rDNA aaggcttcttctgagtgtacttctgcataaaggcgttctgcgggggaaaccgcatctcggtaggcatagtggtttagtgcttgccatatagcagcctggacgggtccctgcagcaccgccatcctcgagg 36663

37,840 37,860 37,880 37,900 37,920 37,940 37,960
KY962518_1 ctcaggcccactttctgcagtgccacaggcacccccccccccccccatagcggctccggcccggccagcctcggctcatttaaaggcaccagccgccgttaccgggggatgggggagtccgagacagaatg 37830
human_rDNA ctcaggcccactttctgcagtgccacaggca-cccccccccccccatagcggctccggcccggccagccccggctcatttaaaggcaccagccgccgttaccgggggatgggggagtccgagacagaatg 36792

37,980 38,000 38,020 38,040 38,060 38,080
KY962518_1 acttctttatcctgctgactctggaaagcccggcgccttgtgatccattgcaaaccgagagtcacctcgtgtttagaacacggatccactcccaagttcagtgggggggatgtgagggggtgtggcaggtag 37960
human_rDNA acttctttatcctgctgactctggaaagcccggcgccttgtgatccattgcaaacggagagtcacctcgtgtttagaacacggatccactcccaagttcagtgggggggatgtgagggggtgtggcaggtag 36922

38,100 38,120 38,140 38,160 38,180 38,200 38,220
KY962518_1 gacgaaggactctcttccttctgattcggtctgcacagtggggcctagggctggagctctctccgtgcggaccgctgactccctctaccttgggttccctcggccccaccctggaacgccgggccttggc 38090
human_rDNA gacgaaggactctcttccttctgattcggtctgcacagtggggcctagggctggagctctctccgtgcggaccgctgactccctctaccttgggttccctcggccccaccctggaacgccgggccttggc 37052

38,240 38,260 38,280 38,300 38,320 38,340
KY962518_1 agattctggcccttcctggcccttcagtcgctgtcagaaaccccatctcatgctcggatgccccgagtgactgtggctcgcacctctccggaaacattggaaatctctcctctacgcgcggccacctgaa 38220
human_rDNA agattctggcccttcctggcccttcagtcgctgtcagaaaccccatctcatgctcggatgccccgagtgactgtggctcgcacctctccggaaacattggaaatctctcctctacgcgcggccacctgaa 37182

38,360 38,380 38,400 38,420 38,440 38,460 38,480
KY962518_1 accacaggagctcgggacacacgtgctttcgggagagaatgctgagagtctcttgccgactctctcttgacttgagttcttcgtgggtgcgtggttaagacgtagtgagaccagatgtattaactcaggc 38350
human_rDNA accacaggagctcgggacacacgtgctttcgggagagaatgctgagagtctcttgccgactctctcttgacttgagttcttcgtgggtgcgtggttaagacgtagtgagaccagatgtattaactcaggc 37312

38,500 38,520 38,540 38,560 38,580 38,600
KY962518_1 cgggtgctggtggctcacgcctgtaaccccaacactttgggaggccgaggccgtaggatccctcgaggaatcgcctaaccctggggaggttgaggttgcagtgagtgagccatagttgtgtcactgtgct 38480
human_rDNA cgggtgctggtggctcacgcctgtaaccccaacactttgggaggccgaggccgtaggatccctcgaggaatcgcctaaccctggggaggttgaggttgcagtgagtgagccatagttgtgtcactgtgct 37442

38,620 38,640 38,660 38,680 38,700 38,720 38,740
KY962518_1 ccagtctgggcgaaagacagaatgaggccctgcca----caggcaggcaggcaggcaggcaggcagaaagacaacagctgtattatgttcttctcagggtaggaagcaaaaataacagaatacagcactt 38606
human_rDNA ccagtctgggcgaaagacagaatgaggccctgccacaggcaggcaggcaggcaggcaggcaggcagaaagacaacagctgtattatgttcttctcagggtaggaagcaaaaataacagaatacagcactt 37572

38,760 38,780 38,800 38,820 38,840 38,860
KY962518_1 aattaattttttttttttcccttcggacggagtttcactcttggtgcccacgctggagtgcagtggcaccatctcggctcaccgcaacctccacctcccgcgttcaagcgattctcctgcctcagcctcc 38736
human_rDNA aattaa-tttttttttttcccttcggacggagtttcactcttggtgcccacgctggagtgcagtggcaccatctcggctcaccgcaacctccacctcccgcgttcaagcgattctcctgcctcagcctcc 37701

38,880 38,900 38,920 38,940 38,960 38,980 39,000
KY962518_1 tgagtagctgggattacagggaggagccaccacacccagctgatttgtattgttagtagagacggcatttctccatgtgggtcaggctggtctcgaactggcgaccccagtggatctgcccgccccggc 38866
human_rDNA tgagtagctgggattacagggaggagccaccacacccggctgatttgtattgttagtagagacggcatttctccatgtgggtcaggctggtctcgaactggcgaccccagtggatctgcccgccccggc 37831

39,020 39,040 39,060 39,080 39,100 39,120
KY962518_1 ctcccaaagtgctggggtgacaggcgtgagccatcgtgactggccggctacgtttatttatttatttttttaattattttacttttttttagttttccattttaatctatttatttatttacatttattt 38996
human_rDNA ctcccaaagtgctggggtgacaggcgtgagccatcgtgactggccggctacgtttatttatttatttttttaattattttacttttttttagttttccattttaatctatttatttatttacatttattt 37961

39,140 39,160 39,180 39,200 39,220 39,240 39,260
KY962518_1 atttatttatttatttacttatttatttattttcgagacagactctcgctctgctgcccaggctggagtgcagcggcgtgatctcggctcactgcaagctccgcctcccgggttcacgccattctcctgc 39126
human_rDNA atttatttatttatttacttatttatttattttcgagacagactctcgctctgctgcccaggctggagtgcagcggcgtgatctcggctcactgcaagctccgcctcccgggttcacgccattctcctgc 38091

39,280 39,300 39,320 39,340 39,360 39,380
KY962518_1 ctcagcctcccaagtagctgggactacaggcgcccgccaccgtgcccggctaactttttgtattttgagtagagatggggtttcactgtggtagccaggatggtctcgatctcctgaccccgtgatccgt 39256
human_rDNA ctcagcctcccaagtagctgggactacaggcgcccgccaccgtgcccggctaactttttgtattttgagtagagatggggtttcactgtggtagccaggatggtctcgatctcctgaccccgtgatccgt 38221

39,400 39,420 39,440 39,460 39,480 39,500 39,520
KY962518_1 ccacctcggcctcccaaagtgctgggatgacaggcgtgagccaccgcccccggcctatttatctatttattaactttgagtccaggttatgaaaccagttagttttgtaattttttttttttttttttt 39386
human_rDNA ccacctcggcctcccaaagtgctgggatgacaggcgtgagccaccgcccccggcctatttatctatttattaactttgagtccaggttatgaaaccagttagttttgtaa-tttttttttttttttttt 38350

```
                              39,540              39,560              39,580              39,600              39,620              39,640
KY962518_1  ttttttgagacgaggtttcaccgtgttgccaaggcttggaccgagggatccaccggccctcggcctcccaaaagtgcggggatgacaggcgcgagcctaccgcgcccggaccccccctttccccttcccc 39516
human_rDNA  ttttttgagacgaggtttcaccgtgttgccaaggcttggaccgagggatccaccggccctcggcctcccaaaagtgcggggatgacaggcgcgagcctaccgcgcccggaccccccctttccccttcccc 38480
                  39,660              39,680              39,700              39,720              39,740              39,760              39,780
KY962518_1  cgcttgtcttcccgacagacagtttcacggcagagcgtttggctggcgtgcttaaactcattctaaatagaaatttgggacgtcagcttctggcctcacggactctgagccgaggagtcccctggtctgt 39646
human_rDNA  cgcttgtcttcccgacagacagtttcacggcagagcgtttggctggcgtgcttaaactcattctaaatagaaatttgggacgtcagcttctggcctcacggactctgagccgaggagtcccctggtctgt 38610
                              39,800              39,820              39,840              39,860              39,880              39,900
KY962518_1  ctatcacaggaccgtacacgtaaggaggagaaaaatcgtaacgttcaaagtcagtcattttgtgatacagaaatacacggattcacccaaaacacagaaagcagtcttttagaaatggccttagccctgg 39776
human_rDNA  ctatcacaggaccgtacacgtaaggaggagaaaaatcgtaacgttcaaagtcagtcattttgtgatacagaaatacacggattcacccaaaacacagaaagcagtcttttagaaatggccttagccctgg 38740
                  39,920              39,940              39,960              39,980              40,000              40,020              40,040
KY962518_1  tgtccgtgccagtgattcttttcggtttggaccttgactgagaggattcccagtcggtctctcgtctctggacggaagttccagatgatccgatgggtgggggacttaggctgcgtccccccaggagccc 39906
human_rDNA  tgtccgtgccagtgattcttttcggtttggaccttgactgagaggattcccagtcggtctctcgtctctggacggaagttccagatgatccgatgggtgggggacttaggctgcgtccccccaggagccc 38870
                              40,060              40,080              40,100              40,120              40,140              40,160
KY962518_1  tggtcgattagttgtggggatcgccttggagggcgcggtgacccactgtgctgtgggagcctccatccttccccccaccccctccccaggggatcccaattcattccgggctgacacgctcactggcagg 40036
human_rDNA  tggtcgattagttgtggggatcgccttggagggcgcggtgacccactgtgctgtgggagcctccatccttccccccaccccctccccaggggatcccaattcattccgggctgacacgctcactggcagg 39000
                  40,180              40,200              40,220              40,240              40,260              40,280              40,300
KY962518_1  cgtcgggcatcacctagcggtcactgttactctgaaaacggaggcctcacagaggaagggagcaccaggccgcctgcgcacagcctggggcaactgtgtcttctccaccgcccccgcccccacctccaag 40166
human_rDNA  cgtcgggcatcacctagcggtcactgttactctgaaaacggaggcctcacagaggaagggagcaccaggccgcctgcgcacagcctggggcaactgtgtcttctccaccgcccccgcccccacctccaag 39130
                              40,320              40,340              40,360              40,380              40,400              40,420
KY962518_1  ttcctccctcccttgttgcctaggaaatcgccactttgacgaccgggtctgattgacctttgatcaggcaaaaacgaacaaacagataaataaataaaataacacaaaagtaactaactaaataaaataa 40296
human_rDNA  ttcctccctcccttgttgcctaggaaatcgccactttgacgactgggtctgattgacctttgatcaggcaaaaacgaacaaacagataaatcaataaaataacacaaaagtaactaactaaataaaataa 39260
                  40,440              40,460              40,480              40,500              40,520              40,540              40,560
KY962518_1  gtcaatacaacccattacaatacaataagatacgatacgataggatgcgataggatacgataggatacaatacaatacgatacg----------------------------------------atacaa 40386
human_rDNA  gtcaatacaacccattacaatacaataagatacgatacgataggatgcgataggatacgataggatacaatacaatacgatacgatacaatacaatacaatacaatacaatacaatacaatacaatacaa 39390
                              40,580              40,600              40,620              40,640              40,660              40,680
KY962518_1  tacaatacaatacaatacaatacaatacaatacaatacaatacaatacgccgggcgcggtggctcatgcctgtcatcccgtcactttgggatgccgaggtggacgcatcacctgaagtcgggagttggag 40516
human_rDNA  tacaatacaatacaatacaatacaatacaatacaatacaatacaatacgccgggcgcggtggctcatgcctgtcatcccgtcactttgggatgccgaggtggacgcatcacctgaagtcgggagttggag 39520
                  40,700              40,720              40,740              40,760              40,780              40,800              40,820
KY962518_1  acaagcccgaccaacatggagaaatcccgtctcaattgaaaatacaaaactagccgggcgcggtggcacatgcctataatcccagctgctaggaaggctgaggcaggagaatcgcttgaacctgggaagc 40646
human_rDNA  acaagcccgaccaacatggagaaatcccgtctcaattgaaaatacaaaactagccgggcgcggtggcacatgcctataatcccagctgctaggaaggctgaggcaggagaatcgcttgaacctgggaagc 39650
                              40,840              40,860              40,880              40,900              40,920              40,940
KY962518_1  ggaggttgcagtgagccgagattgcgccatcgcactccagtctgagcaacaagagcgaaactccgtctcaaaaataaatacataaataaatacatacatacatacatacatacatacatacataaattaa 40776
human_rDNA  ggaggttgcagtgagccgagattgcgccatcgcactccagtctgagcaacaagagcgaaactccgtctcaaaaataaatacataaataa----atacatacatacatacatacatacatacataaattaa 39776
                  40,960              40,980              41,000              41,020              41,040              41,060              41,080
KY962518_1  aataaataaataaaataaaataaataaatgggccctgcgcggtggctcaagcctgtcatcccctcactttgggaggccaaggccggtggatcaagaggcggtcagaccaacagggccagtatggtgaaac 40906
human_rDNA  aataaataaataaaataaaataaataaatgggccctgcgcggtggctcaagcctgtcatcccctcactttgggaggccaaggccggtggatcaagaggcggtcagaccaacagggccagtatggtgaaac 39906
                              41,100              41,120              41,140              41,160              41,180              41,200
KY962518_1  cccgtctctactcacaatacacaacattagccgggcgctgtgctgtgctgtactgtctgtaatcccagctactcgggaggccgagctgaggcaggagaatcgcttgaacctgggaggcggaggttgcagt 41036
human_rDNA  cccgtctctactcacaatacacaacattagccgggcgctgtgctgtgctgtactgtctgtaatcccagctactcgggaggccgagctgaggcaggagaatcgcttgaacctgggaggcggaggttgcagt 40036
                  41,220              41,240              41,260              41,280              41,300              41,320              41,340
KY962518_1  gagccgagatcgcgccactgcaacccagcctgggcgacagagcgagactccgtctccaaaaaatgaaaatgaaaatgaaacgcaacaaaataattaaaaagtgagtttctggggaaaaagaagaaaagaa 41166
human_rDNA  gagccgagatcgcgccactgcaacccagcctgggcgacagagcgagactccgtctccaaaaaatgaaaatgaaaatgaaacgcaacaaaataattaaaaagtgagtttctggggaaaaagaagaaaagaa 40166
                              41,360              41,380              41,400              41,420              41,440              41,460
KY962518_1  aaaagaaaaaaacaacaaaacagaacaaccccaccgtgacatacacgtacgcctctcgcctttcgaggcctcaaacacgttaggaattatgcgtgatttctttttttaacttcattttatgttattatca 41296
human_rDNA  aaaagaaaaaaacaacaaaacagaacaaccccaccgtgacatacacgtacgcctctcgcctttcgaggcctcaaacacgttaggaattatgcgtgatttctttttttaacttcattttatgttattatcg 40296
                  41,480              41,500              41,520              41,540              41,560              41,580              41,600
KY962518_1  tgattgatgtttcgagacggagtctcggaggcccgccctccctggttgcccagacaaccccgggagacagaccctggctgggcccgattgttcttctccttggtcaggggtttccttgtctttcttcgtg 41426
human_rDNA  tgattgatgtttcgagacggagtctcggaggcccgccctccctggttgcccagacaaccccgggagacagaccctggctgggcccgattgttcttctccttggtcaggggtttccttgtctttcttcgtg 40426
```

```
             41,620 41,640 41,660 41,680 41,700 41,720
KY962518_1   tctttaacccgcgtggactcttccgctcgggtttgacagatggcagctccactttaggccttgttgttgttggggactttcctgattctccccagatgtagtgaaagcaggtagatttgccttgcctgga 41556
human_rDNA   tctttaacccgcgtggactcttccgctcgggtttgacagatggcagctccactttaggccttgttgttgttggggactttcctgattctccccagatgtagtgaaagcaggtagatttgccttgcctgga 40556
             41,740 41,760 41,780 41,800 41,820 41,840 41,860
KY962518_1   cttgcctggccttgcctttttctttctttctttctttattactttctctttttcttcttcttcttcttcttcttcttcttcttcttcttcttcttcttct-----------------tttttttttttg 41669
human_rDNA   cttgcctggccttgcctttttctttctttctttctttattactttctctttttcttcttcttcttcttcttcttcttcttcttcttcttcttcttcttcttcttcttcttcttcttctttttttttg 40686
             41,880 41,900 41,920 41,940 41,960 41,980
KY962518_1   agacagagtttcactcttgttgcccaggctagagggcaatggtgcgatctcggctcaccgcaccctccgcctcccaggttcaagcgattctcctgcctcagcctcctgattagctgggattacaggcatg 41799
human_rDNA   agacagagtttcactcttgttgcccaggctagagggcaatggtgcgatctcggctcaccgcaccctccgcctcccaggttcaagcgattctcctgcctcagcctcctgattagctgggattacaggcatg 40816
             42,000 42,020 42,040 42,060 42,080 42,100 42,120
KY962518_1   ggccaccgtgcctggctgatgtttgtactttagtagagacggtgtttttccatgttggtcaggctggtctcccactcccaacctcaggtggtccgcctgccttagcctcccaaagtgctgggatgacag 41929
human_rDNA   ggccaccgtgcctggctgatgtttgtactttagtagagacggtgtttttccatgttggtcaggctggtctcccactcccaacctcaggtggtccgcctgccttagcctcccaaagtgctgggatgacag 40946
             42,140 42,160 42,180 42,200 42,220 42,240
KY962518_1   gcgtgagccaccgcgcccagcctctctctctctctctctctctctctctctctctctctctctcgctcgcttgcttgcttgctttcgtgctttcttgctttcccgttttcttgctttctttctttctttc 42059
human_rDNA   gcgtgagccaccgcgcccagcctctctctctctctctctctctctctctctctctctctctctcgctc----gcttgcttgctttcgtgctttcttgctttcccgttttcttgctttctttctttctttc 41072
             42,260 42,280 42,300 42,320 42,340 42,360 42,380
KY962518_1   gtttctttcatgcttgctttcttgcttgcttgcttgctttcgtgctttcttgctttcctgt----------tttctttctttctttctttcttttg----------------------------------- 42145
human_rDNA   gtttctttcatgcttgctttcttgcttgcttgcttgctttcgtgctttcttgctttcctgttttctttttctttctttctttctttcttttgtttctttcttgcttgctttcttgcttgcttgctt 41202
             42,400 42,420 42,440 42,460 42,480 42,500
KY962518_1   --------------------------------------tttctttcttgcttgctttcttgcttgcttgctttcgtgctttcttgttttctcgatttctttctttctttgtttctttcctgcttgcttt 42237
human_rDNA   gctttcgtgctttcttgctttcctgtttctttctttctttctttcttgcttgctttcttgcttgcttgctttcgtgctttcttgttttctcgctttctttctttctttgtttctttcctgcttgcttt 41332
             42,520 42,540 42,560 42,580 42,600 42,620 42,640
KY962518_1   cttgcttgattgctttcgtgctttcttgctttcttgtttctttctttctttgtttctttctttcttgcttcctgttttcttgctttcttgcttgcttgctttcgtgctttcttgttttcttgctttc 42367
human_rDNA   cttgcttgattgctttcgtgctttcttgctttcttgtttctttctttctttgtttctttctttcttgcttcctgttttcttgctttcttgcttgcttgctttcgtgctttcttgttttcttgctttc 41462
             42,660 42,680 42,700 42,720 42,740 42,760
KY962518_1   tttctttgtttctttcttgcttgctttcttgcttccttgttttcttgctttcttgcttgcttgctttcgtgctttctttcttgctttcttttctttctttctttcttttctttctttcttgctttct 42497
human_rDNA   tttctttgtttctttcttgcttgctttcttgcttccttgttttcttgctttcttgcttgcttgctttcgtgctttctttcttgctttcttttctttctttctttcttttctttctttcttgctttct 41592
             42,780 42,800 42,820 42,840 42,860 42,880 42,900
KY962518_1   tttctttcattcattcattctttctttctttccttctttctttctttctttctttcta--------------------tctttctttctttctttctttctgtttcgtcctttgagacagagtttcactctt 42607
human_rDNA   tttctttcattcattcattctttctttctttccttctttctttctttctttctttctatctttctttctttctttctttctttctttctttctttctttctgtttcgtcctttgagacagagtttcactctt 41722
             42,920 42,940 42,960 42,980 43,000 43,020
KY962518_1   gtttccacggctagagtgcaatggcgcgatcttggctcaccgcaccttccgcctcccgggttcgagcgcttctcctgcctcagcctcccgattagcggggattacagggaggcacccccacgcctggctt 42737
human_rDNA   gtttccacggctagagtgcaatggcgcgatcttggctcaccgcaccttccgcctcccgggttcgagcgcttctcctgcctcagcctcccgattagcggggattacagggaggcacccccacgcctggctt 41852
             43,040 43,060 43,080 43,100 43,120 43,140 43,160
KY962518_1   ggctgatgtttgtgtttttagtaggcacgccgtgtctctccatgttgctcaggctggtctccaactcccgacctcctgtgatgcgcccacctcggcctctcgaagtgctgggatgacggcgtgagccac 42867
human_rDNA   ggctgatgtttgtgtttttagtaggcacgccgtgtctctccatgttgctcaggctggtctccaactcccgacctcctgtgatgcgcccacctcggcctctcgaagtgctgggatgacggcgtgagccac 41982
             43,180 43,200 43,220 43,240 43,260 43,280
KY962518_1   cgtgcccggcctgttgactcatttcgctttttttatttctttcgtttccacgcgtttacttatatgtattaatgtaaacgtttctgtacgcttatatgcaaacaacgacaacgtgtatctctgcattgaat 42997
human_rDNA   cgtgcccggcctgttgactcatttcgctttttttatttctttcgtttccacgcgtttacttatatgtattaatgtaaacgtttctgtacgcttatatgcaaacaacgacaacgtgtatctctgcattgaat 42112
             43,300 43,320 43,340 43,360 43,380 43,400 43,420
KY962518_1   actcttgcgtatggtaaatacgtatcggttgtatggaaatagacttctgtatgatagatgtaggtgtctgtgttatacaaataaatacacatcgctctataaagaagggatcgtcgataaagacgtttat 43127
human_rDNA   actcttgcgtatggtaaatacgtatcggttgtatggaaatagacttctgtatgatagatgtaggtgtttgtgttatacaaataaatacacatcgctctataaagaagggatcgtcgataaagacgtttat 42242
             43,440 43,460 43,480 43,500 43,520 43,540
KY962518_1   tttacgtatgaaaagcgtcgtatttatgtgtgtaaatgaacgagcgtacgtagttatctctgtttctttcttcctctccttcgtgtttttcttccttcctttcttcctttctctccttctttaggtttt 43257
human_rDNA   tttacgtatgaaaagcgtcgtatttatgtgtgtaaatgaacgagcgtacgtagttatctctgtttctttcttcctctccttcgtgtttttcttccttcctttcttcctttctctccttctttaggtttt 42372
             43,560 43,580 43,600 43,620 43,640 43,660 43,680
KY962518_1   tcttcctctcttcctttccttctttctctctttctgtccttttttccttcgtgctttatttctctttcgttccctgtgtttccttctttttttctttcctctctgtttcttttcccttctttccttcgtt 43387
human_rDNA   tcttcctctcttcctttccttctttctctctttctgtccttttttccttcgtgctttatttctctttcgttccctgtgtttccttctttttttctttcctctctgtttcttttcccttctttccttcgtt 42502
```

```
                43,700              43,720              43,740              43,760              43,780              43,800
KY962518_1  tctttcctcattctttctctcttttcgtgtttctttccttcccgtctgtcttttaaaaaatggagtgtttcagaagtttactttgtgtatctacgttttctaaattgtctctcttttctccattgtctt 43517
human_rDNA  tctttcctcattctttctctcttttcgtgtttctttccttcccgtctgtcttttaaaaaatggagtgtttcagaagtttactttgtgtatctacgttttctaaattgtctctcttttctccattttctt 42632
          43,820              43,840              43,860              43,880              43,900              43,920              43,940
KY962518_1  cctccctccctccctccctccctccctgctcccttccctccctccttccctttcgccatctgtctcttttccccactcccctcccccgtctgtctctgcgtggattccggaagagcctacgcattctgc 43647
human_rDNA  cctccctccctccctccctccctccctgctcccttccctccctccttccctttcgccatctgtctcttttccccactcccctcccccgtctgtctctgcgtggattccggaagagcctacgcattctgc 42762
                43,960              43,980              44,000              44,020              44,040              44,060
KY962518_1  ctctccgtgtgtctgcagcgacccgcgaccgagtccttgtgtgttctttctccctccctccctccctccctccctccctccctgcttccgagaggcatctccaaacacccacgcgccgtgggttgtcttc 43777
human_rDNA  ctctccgtgtgtctgcagcgacccgcgaccgagtccttgtgtgttctttctccctccctcactccctccctccctccctccctgcttccgagaggcatctccaaacacccacgcgccgtgggttgtcttc 42892
          44,080              44,100              44,120              44,140              44,160              44,180              44,200
KY962518_1  tgactctgtcgcggtcgaggcagagacgcgttttgggcaccgtttgtgtggggttggggcagagggctgcgttttcggcctcgggaagagcttctcgactcacggtttcgctttcgcggtccacggcc 43907
human_rDNA  tgactctgtcgcggtcgaggcagagacgcgttttgggcaccgtttgtgtggggttggggcagagggctgcgttttcggcctcgggaagagcttctcgactcacggtttcgctttcgcggtccacggcc 43022
                44,220              44,240              44,260              44,280              44,300              44,320
KY962518_1  gccctgccagccggatctgtctcgctgacgtccgcggcggttgtcgggctccatctggcggccgctttgagatcgtgctctcggcttccggagctgcggtggcagctgccgagggaggggaccgtccccg 44037
human_rDNA  gccctgccagccggatctgtctcgctgacgtccgcggcggttgtcgggctccatctggcggccgctttgagatcgtgctctcggcttccggagctgcggtggcagctgccgagggaggggaccgtccccg 43152
          44,340              44,360              44,380              44,400              44,420              44,440              44,460
KY962518_1  ctgtgagctaggcagagctccggaaagcccgcggtcgtcagcccggctggcccggtggcgccagagctgtggcgcgtcgcttgtgagtcacagctctggcgtgcaggtttatgtgggggagaggctgtcg 44167
human_rDNA  ctgtgagctaggcagagctccggaaagcccgcggtcgtcagcccggctggcccggtggcgccagagctgtggcgcgtcgcttgtgagtcacagctctggcgtgcaggtttatgtgggggagaggctgtcg 43282
                44,480              44,500              44,520              44,540              44,560              44,580
KY962518_1  ctgcgcttctgggcccgcggcgggcgtggggctgcccgggccggtcgaccagcgcgccgtagctcccgaggcccgagccgcgacccgcggggacccgccgcgcgtggcgcgggaggctggggacgccctt 44297
human_rDNA  ctgcgcttctgggcccgcggcgggcgtggggctgcccgggccggtcgaccagcgcgccgtagctcccgaggcccgagccgcgacccgcggggacccgccgcgcgtggcgcgggaggctggggacgccctt 43412
          44,600              44,620              44,640              44,660              44,680              44,700              44,720
KY962518_1  cccggcccggtcgcgggtccgcgctcatcctggccgtctgaggcggcggccgaattcgtttccgagtccccgtggggagccggggaccgtcccgcccccgtcccccgggtgccgggga------------ 44415
human_rDNA  cccggcccggtcgcgggtccgcgctcatcctggccgtctgaggcggcggccgaattcgtttccgagtccccgtggggagccggggaccgtcccgcccccgtcccccgggtgccggggagcggtccccggg 43542
                44,740              44,760              44,780              44,800              44,820              44,840
KY962518_1  -------gcggtccctctgccgcgatcctttctggcgagtccccgtgcggagtcggagagcgctccctgagcgcgcgtgcggcccgagaggtcgcgcctggccggccttcggtccctcgtgtgtcccggt 44538
human_rDNA  ccgggccgcggtccctctgccgcgatcctttctggcgagtccccgtgcggagtcggagagcgctccctgagcgcgcgtgcggcccgagaggtcgcgcctggccggccttcggtccctcgtgtgtcccggt 43672
          44,860              44,880              44,900              44,920              44,940              44,960              44,980
KY962518_1  cgtaggaggggccggccgaaaatgcttccggctcccgctctggagacacgggccggcccccctgcgtgtggcacgggcggccgggagggcgtccccggcccggcgctgctcccgcgtgtgtcctggggttg 44668
human_rDNA  cgtaggaggggccggccgaaaatgcttccggctcccgctctggagacacgggccggcccccctgcgtgtggcacgggcggccgggagggcgtccccggcccggcgctgctcccgcgtgtgtcctggggttg 43802
                45,000              45,020              45,040              45,060              45,080              45,100
KY962518_1  accagagggccccgggcgctccgtgtgtggctgcgatggtggcgttttggggacaggtgtccgtgtcgcgcgtcgcctgggccggcggcgtggtcggtgacgcgacctcccggccccgggggaggtata 44798
human_rDNA  accagagggccccgggcgctccgtgtgtggctgcgatggtggcgttttggggacaggtgtccgtgtcgcgcgtcgcctgggccggcggcgtggtcggtgacgcgacctcccggccccgggggaggtata 43932
          45,120              45,140
KY962518_1  tctttcgctccgagtcggcatttgggccgccgggttatt 44838
human_rDNA  tctttcgctccgagtcggcatttgggccgccgggttatt 43972
```
