# Supplementary material for: The conservation landscape of the human ribosomal RNA gene repeats
Source: PLoS One. 2018 Dec 5;13(12):e0207531. doi: 10.1371/journal.pone.0207531 (PMC6281188; doi:10.1371/journal.pone.0207531)
Supplement: S1 File — (DOCX) [file pone.0207531.s007.docx]

**The conservation landscape of the human ribosomal RNA gene repeats**

Saumya Agrawal^1^ and Austen R.D. Ganley^1,2,3^

^1^*Institute of Natural and Mathematical Sciences, Massey University, Private Bag 102-904, Auckland 0632, New Zealand*

^2^ *School of Biological Sciences, University of Auckland, Private Bag 92019, Auckland 1142, New Zealand (current address)*

^3^Corresponding author: Email *a.ganley@auckland.ac.nz*

## Supporting Methods

## Primate rDNA sequence construction

The following steps were repeated for each of the primate species to obtain the rDNA repeat unit sequence:

**Step-1:**Potential rDNA containing contigs were identified by screening the obtained WGA with human rDNA unit using BLAST.

**Step-2:** The rDNA aligned contigs with an average read coverage <10 were removed. The read coverage for rDNA containing contigs was obtained using CLC genomic workbench (CLC bio, Inc.). To remove the segmentally duplicated rDNA regions in other parts of the genome, contigs smaller than 1kb were also discarded. The low coverage ends (reads coverage <5) of the remaining contigs were trimmed before proceeding.

**Step-3:** Depending on the number of rDNA units present in the contigs obtained from Step-2, two different strategies were employed to construct the primate rDNA repeat unit sequence. The strategies are described as below:

The first strategy was employed if Step-2 yielded a contig longer than the human rDNA unit sequence (assuming that all the primate rDNA units are ~40 kb) and thus has one or more complete rDNA units. This contig was selected for the construction of the rDNA sequence. The partial unit in the contig was removed to obtain the complete rDNA repeat unit sequence. The presence of more than one rDNA unit in the contig was established by self-comparison using BLAST.

The second strategy was employed if the contigs obtained from Step-2 were smaller than the human rDNA sequence and more than one overlapping contig was required to completely cover the human rDNA sequence. The overlapping contigs were merged using Consed (ver. 19) (1). Consed first determines the overlapping regions between the contigs using cross_match (<http://www.phrap.org/phredphrap/general.html>). Next it merges the reads in the overlapping regions and creates a new contig by generating a consensus sequence using the merged reads. The files containing contig information in .ACE format that were generated by Arachne were used as input for Consed.

**d) Step-4:** The contig obtained from Step-3 was rearranged such that the base 1 of the sequenced is the start of the 45S rRNA coding region. The 45S rRNA coding region in the primate rDNA sequence was demarcated by comparing it with the human 45S rRNA coding sequence using BLAST.

## Parameters for ChIP-seq analysis

The following steps were performed to analyze all the histone modification and TF datasets for the seven cell lines:

**Step-1:** Low quality ends of reads were trimmed using DynamicTrim with quality score cutoff of 13, and short reads were removed with a length cutoff of 25 bp using LengthSort. Both programs are part of the SolexaQA package (2).

**Step-2:** Processed reads were mapped to the modified human genome assembly using bowtie (ver. 0.12.8) (3). The following parameters were used to map the reads: uniquely mapped reads to the genome (-m 1), maximum three mismatches in the alignment (-v 3), and seed length (-l 30). The alignment was obtained in SAM file format.

**command:** bowtie -l 30 -v 3 -p 7 --chunkmbs 1024 -a --best --strata -m 1 <modified_human_genome_index><ChIP_seq_trimmed.fastq> -S <ChIP_seq_mapped.sam>

**c) Step-3:** Mapped reads were sorted according to the position mapped to the reference sequence using the command SortSam.jar. To remove the coverage bias and provide equal representation of all the regions, multiple reads mapped to same location were removed using the command MarkDuplicates.jar. All the replicates for each sample were merged using the command MergeSamFiles.jar. All three commands are part of Picard tools (ver. 1.6.1).

**Command:** java –jar SortSam.jar I=<mapped_ChIPseq_chr21.sam> O=<mapped_ChIPseq _chr21_sorted.sam> SO=coordinate MAX_RECORDS_IN_RAM=5000000

**Command:** java -jar MarkDuplicates.jar INPUT=<mapped_ChIPseq_chr21_sorted.sam> O= <mapped_ChIPseq _chr21_dr.sam> M=<stat_dr.txt> REMOVE_DUPLICATES=true

**Command:** java -jar MergeSamFiles.jar I=<mapped_ChIPseq _chr21_dr1.sam> I=<mapped_ChIPseq_chr21_dr2.sam> O=<mapped_ChIPseq_chr21_dr_merge.sam> SORT_ORDER=coordinate

**d) Step-4:** The fragment size was calculated using the merged SAM file withrun_spp.R ver. 1.11 (http://code.google.com/p/phantompeakqualtools) (4).

**Command:** Rscriptrun_spp.R -c=<ChIP_seq_mapped_dr_merge.bam> -savp -out=<ChIP_seq_mapped_dr_merge.txt>

**e) Step-5:** The merged and sorted SAM files were used to call peaks using the callpeak function of MACS2 (ver. 2.0.10.20120913; <https://github.com/taoliu/MACS/>) (5,6). Noise was removed from the signal by subtracting the corresponding Input signal using thebdgcmp function. Both callpeak and bdgcmp are functions of MACS2.

**Command:** macs2 callpeak -c ChIP_seq_control_mapped_dr_merge.sam -t ChIP_seq_mapped_dr_merge.sam -g 'hs' --keep-dup all -n ChIP_seq --trackline -B -m 5 50 --nomodel --shiftsize<fragment_length/2>

**Command:** macs2 bdgcmp -t ChIP_seq_treat_pileup.bdg -c ChIP_seq_control_lambda.bdg -o ChIP_seq_treat_minus_linear.bdg -m subtract

**f) Step-6:** Peaks corresponding to the rDNA sequence were extracted for further analysis.

**g) Step-7:** The peaks were visualized using Integrative Genomics Viewer (IGV) ver. 2.3 (7,8).

## Parameters for transcriptome profiling

The following steps were performed for all the selected long poly(A)+ and long poly(A)- RNA-seq datasets:

**Step-1:** The RNA-seq data were first mapped to the rRNA coding region using bowtie (ver 0.12.8) to filter out the rRNA reads.

The cleaned data were mapped to the modified human genome assembly using STAR aligner (ver. 2.2.0) (9). The following parameters were used to map the reads: the first 13 bp at the 5’ end were trimmed before mapping (--clip5pNbases 1), and mapped 1 times on the genome (--outFilterMultimapNmax 1)**.** Maximum 10 mismatches for cell line and maximum 5 mismatches for tissue data (--outFilterMismatchNmax 10) were allowed in the called hit. The variation in mismatches was based on the read length. The alignments were obtained in SAM file format.

**Command:** STAR --genomeDirgenome_STAR_index --readFilesIn rnaseq_1.fastq rnaseq_2.fastq --outSAMstrandFieldintronMotif --clip5pNbases 13 --runThreadN 10 --outFilterMultimapScoreRange 1 --outFilterMultimapNmax 1 --outFilterMismatchNmax 10 --outFilterIntronMotifsRemoveNoncanonical

**d) Step-3:** All the data were sorted according to the reference sequence coordinates using the command SortSamFiles.jar from Picard tools (ver. 1.6.1).

**Command:** java –jar SortSam.jar I=mapped_rnaseq.sam O=mapped_rnaseq_sorted.sam SO=coordinate MAX_RECORDS_IN_RAM=5000000

**e) Step-5:** The sorted SAM files were used as input for Cufflinks (ver 2.2.1) (10) to assemble the RNA-seq data. Default parameters were used for the assembly except the minimum isoform abundance required to be assembled was changed from 1% to 0.5% (-F 0.05) and the reads from the rDNA coding region were masked (–M rdna_coding_region.gff).

**Command:** cufflinks –M rdna_coding_region.gff -N --total-hits-norm -p 8 --no-update-check -F 0.05 –library-type fr-sfirststrand <mapped_rnaseq_sorted.sam>

## Parameters for CAGE analysis

CAGE data for the seven cell lines were obtained from FANTOM 5 (11). CAGE tags were sequenced on Heliscope platform and therefore the reads do not have any quality value. Hence before mapping the reads, the fastq files were converted into fasta files to remove the pseudo-quality information. A repeat masked custom human genome assembly (hg19) was created for the CAGE analysis by inserting masked rDNA sequence into chr21. The following steps were repeated for each sample to obtain the rDNA CAGE peaks:

**Step-1:** The extracted fasta files were mapped to the custom masked human genome assembly using bowtie (ver. 0.12.8). The parameters used for the mapping were: uniquely mapped reads to the genome (-m 1), maximum three mismatches in the alignment (-v 3), and seed length (-l 20). The alignment was obtained in SAM file format. The obtained alignments were sorted using SortSam and were converted to bam files for downstream analysis.

**command:** bowtie -l 20 -v 3 -p 7 --chunkmbs 1024 -a --best --strata -m 1 -f hg19_masked_index sample_CAGE.fasta -S sample.sam

**command:**java -Xmx32g -jar /home/sagrawal/project/nzgl00690/scratch/bin/picard-tools-1.113/SortSam.jar I=sample.sam O=sample_sort.sam SO=coordinate MAX_RECORDS_IN_RAM=10000000

**command:** samtools view –bS sample_sort.sam > sample_sort.bam

**Step-2:** The read coverage of each base for both strands was obtained using the bedcov function of bedtools. The obtained coverage files were combined into one file as per the input file requirement of paraclu (12) to call the clusters.

**Command:**

bedtoolsgenomecov -strand "+" -5 -dz -ibamsample.bam > sample_plus.txt

bedtoolsgenomecov -strand "-" -5 -dz -ibamsample.bam > sample_minus.txt

sed "s/chr21/chr21\t-/g" sample_minus.txt >> sample_combine.txt

sed "s/chr21/chr21\t+/g" sample_plus.txt >> sample_combine.txt

**Step-3:** The regions enriched with CAGE tags were identified using paraclu (ver. 3). Initially, the clusters were identified as the region with minimum 10 tags. The identified clusters were then further processed using the paraclu-cut.sh script.

**command:** paraclu 10 sample_combine.txt > sample_paraclu.txt

**command**: paraclu-cut.sh sample_paraclu.txt > sample_paraclu_clean.gff

**References**

1. Gordon, D., Abajian, C. and Green, P. (1998) Consed: a graphical tool for sequence finishing. *Genome Res*, **8**, 195-202.

2. Cox, M.P., Peterson, D.A. and Biggs, P.J. (2010) SolexaQA: At-a-glance quality assessment of Illumina second-generation sequencing data. *BMC bioinformatics*, **11**, 485.

3. Langmead, B. (2010) Aligning short sequencing reads with Bowtie. *Curr Protoc Bioinformatics*, **Chapter 11**, Unit 11 17.

4. Kharchenko, P.V., Tolstorukov, M.Y. and Park, P.J. (2008) Design and analysis of ChIP-seq experiments for DNA-binding proteins. *Nature biotechnology*, **26**, 1351-1359.

5. Zhang, Y., Liu, T., Meyer, C.A., Eeckhoute, J., Johnson, D.S., Bernstein, B.E., Nusbaum, C., Myers, R.M., Brown, M., Li, W. *et al.* (2008) Model-based analysis of ChIP-Seq (MACS). *Genome biology*, **9**, R137.

6. Feng, J., Liu, T., Qin, B., Zhang, Y. and Liu, X.S. (2012) Identifying ChIP-seq enrichment using MACS. *Nature protocols*, **7**, 1728-1740.

7. Thorvaldsdóttir, H., Robinson, J.T. and Mesirov, J.P. (2013) Integrative Genomics Viewer (IGV): high-performance genomics data visualization and exploration. *Briefings in bioinformatics*, **14**, 178-192.

8. Robinson, J.T., Thorvaldsdóttir, H., Winckler, W., Guttman, M., Lander, E.S., Getz, G. and Mesirov, J.P. (2011) Integrative genomics viewer. *Nature biotechnology*, **29**, 24-26.

9. Dobin, A., Davis, C.A., Schlesinger, F., Drenkow, J., Zaleski, C., Jha, S., Batut, P., Chaisson, M. and Gingeras, T.R. (2013) STAR: ultrafast universal RNA-seq aligner. *Bioinformatics*, **29**, 15-21.

10. Trapnell, C., Roberts, A., Goff, L., Pertea, G., Kim, D., Kelley, D.R., Pimentel, H., Salzberg, S.L., Rinn, J.L. and Pachter, L. (2012) Differential gene and transcript expression analysis of RNA-seq experiments with TopHat and Cufflinks. *Nature protocols*, **7**, 562-578.

11. Forrest, A.R., Kawaji, H., Rehli, M., Baillie, J.K., de Hoon, M.J., Haberle, V., Lassmann, T., Kulakovskiy, I.V., Lizio, M., Itoh, M. *et al.* (2014) A promoter-level mammalian expression atlas. *Nature*, **507**, 462-470.

12. Frith, M.C., Valen, E., Krogh, A., Hayashizaki, Y., Carninci, P. and Sandelin, A. (2008) A code for transcription initiation in mammalian genomes. *Genome Res*, **18**, 1-12.
