# Supplementary material for: The conservation landscape of the human ribosomal RNA gene repeats
Source: PLoS One. 2018 Dec 5;13(12):e0207531. doi: 10.1371/journal.pone.0207531 (PMC6281188; doi:10.1371/journal.pone.0207531)
Supplement: S1 Fig — The rDNA BAC clones for A) Gorilla B) Orangutan C) Gibbon D) Rhesus macaque and E) Common marmoset were used to determine the rDNA unit lengths for these species. For each primate, undigested (U) and I-PpoI digested (D) BACs were run on a FIGE gel (left panels) to determine rDNA unit size. The gels were probed with an 18S rDNA fragment (Southern blots; right panels) to verify the bands contain rDNA. Arrows indicate the rDNA bands. A) Gorilla rDNA BAC bands are ~42 kb. In the digested CH276-103L10 lane, the band above the rDNA band is undigested DNA, as it is the same size as the band in the undigested lane (U). In the digested CH276-120P14 lane, the band above the rDNA band is likely to be E. coli genomic DNA as it is the same size as the band in the undigested lane (D) and has no corresponding signal in the Southern blot. B) Orangutan rDNA BAC bands are ~42 kb. In the digested CH276-103L10 lane, the band above the rDNA band is undigested DNA, as it is the same size as the band in the undigested lane (U). In the digested CH276-120P14 lane, the band above the rDNA band in the gel is E. coli genomic DNA as it is the same size as the band in the undigested lane and has no corresponding signal in the Southern blot (D). C) Gibbon rDNA BAC bands are ~44 kb. In the digested lanes, the band above the rDNA band is undigested DNA as it is the same size as the band in the undigested lane (U). The bands below the rDNA band in CH271-470I24 are probably the BAC backbone. D) Rhesus macaque rDNA BAC bands are ~42.5 kb. In the digested CH250-26D15 lane, the two bands above the rDNA band are a complete rDNA unit with a partial unit (lower band) and E. coli genomic DNA (upper band; same size as the band in the undigested lane and no corresponding signal in the Southern blot). In the digested CH250-46L14 lane, the band above the rDNA band is a complete rDNA unit with a partial unit. In the digested CH259-119I6 lane, the band above the rDNA band is undigested DNA, as it is the same size [file pone.0207531.s008.pdf]

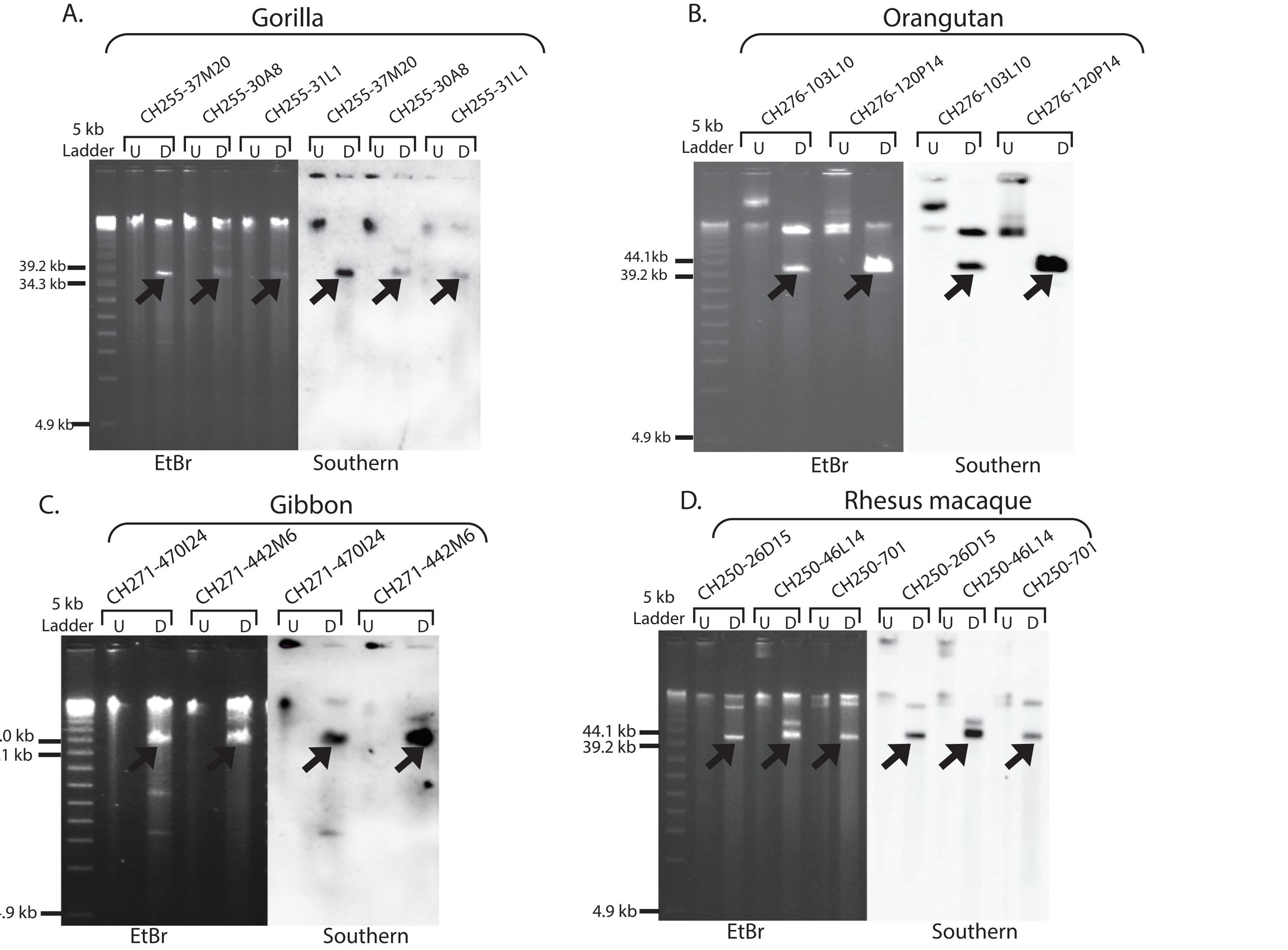

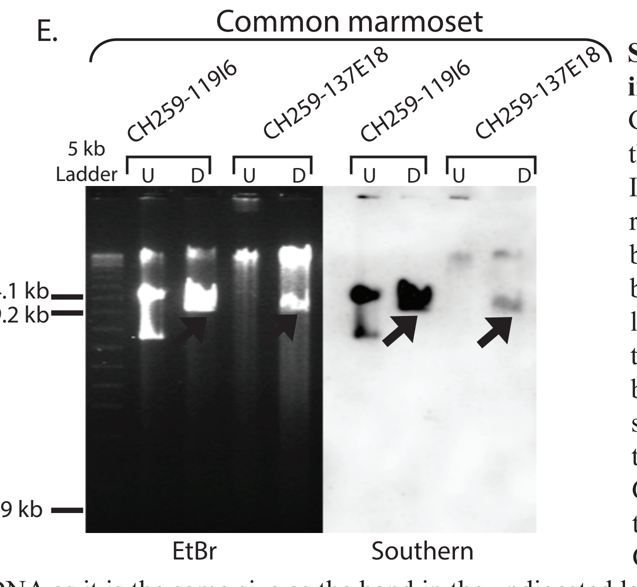

S1 Figure: Estimating the lengths of rDNA units in primate BAC clones. The rDNA BAC clones for **A)** Gorilla **B)** Orangutan **C)** Gibbon **D)** Rhesus macaque and **E)** Common marmoset were used to determine the rDNA unit lengths for these species. For each primate, undigested (U) and I-PpoI digested (D) BACs were run on a FIGE gel (left panels) to determine rDNA unit size. The gels were probed with an 18S rDNA fragment (Southern blots; right panels) to verify the bands contain rDNA. Arrows indicate the rDNA bands. **A)** Gorilla rDNA BAC bands are ~42 kb. In the digested CH276-103L10 lane, the band above the rDNA band is undigested DNA, as it is the same size as the band in the undigested lane (U). In the digested CH276-120P14 lane, the band above the rDNA band is likely to be E. coli genomic DNA as it is the same size as the band in the undigested lane (D) and has no corresponding signal in the Southern blot. **B)** Orangutan rDNA BAC bands are ~42 kb. In the digested CH276-103L10 lane, the band above the rDNA band is undigested DNA, as it is the same size as the band in the undigested lane (U). In the digested CH276-120P14 lane, the band above the rDNA band in the gel is E. coli genomic DNA as it is the same size as the band in the undigested lane and has no corresponding signal in the Southern blot (D). **C)** Gibbon rDNA BAC bands are ~44 kb. In the digested lanes, the band above the rDNA band is undigested DNA as it is the same size as the band in the undigested lane (U). The bands below the rDNA band in CH271-470I24 are probably the BAC backbone. **D)** Rhesus macaque rDNA BAC bands are ~42.5 kb. In the digested CH250-26D15 lane, the two bands above the rDNA band are a complete rDNA unit with a partial unit (lower band) and E.coli genomic DNA (upper band; same size as the band in the undigested lane and no corresponding signal in the Southern blot). In the digested CH250-46L14 lane, the band above the rDNA band is a complete rDNA unit with a partial unit. In the digested CH259-119I6 lane, the band above the rDNA band is undigested DNA, as it is the same size as the band in the undigested lane (U). In the digested CH250-701 lane, the two bands above the rDNA band are a complete rDNA unit with a partial unit (lower band) and E. coli genomic DNA (upper band; same size as the band in the undigested lane and no corresponding signal in the Southern blot). **E)** Common marmoset rDNA BAC bands are ~40 kb. In the digested CH259-137E18 lane, the band above the rDNA band is E. coli genomic DNA as it is the same size as the band in the undigested lane and has no corresponding signal in the Southern blot (D). In the digested CH259-119I6 lane, the band above the rDNA band is undigested DNA as it is the same size as the band in the undigested lane (U). Numbers on the left are the 5 kb ladder sizes used to estimate rDNA unit size.

Agrawal & Ganley,
S1 Figure
