# Supplementary material for: The conservation landscape of the human ribosomal RNA gene repeats
Source: PLoS One. 2018 Dec 5;13(12):e0207531. doi: 10.1371/journal.pone.0207531 (PMC6281188; doi:10.1371/journal.pone.0207531)
Supplement: S2 Fig — The IGS is shown as a grey line. Repeat elements are indicated above and below the IGS as follows: Alu elements (green boxes), LTRs (blue boxes), LINEs (brown boxes), and satellites (orange boxes), with the names alongside. The cdc27 pseudogene is shown as a pink box. Elements above the rDNA are on the forward strand; elements below are on the reverse strand. The start and end coordinates of the IGS are indicated. (PDF) [file pone.0207531.s009.pdf]

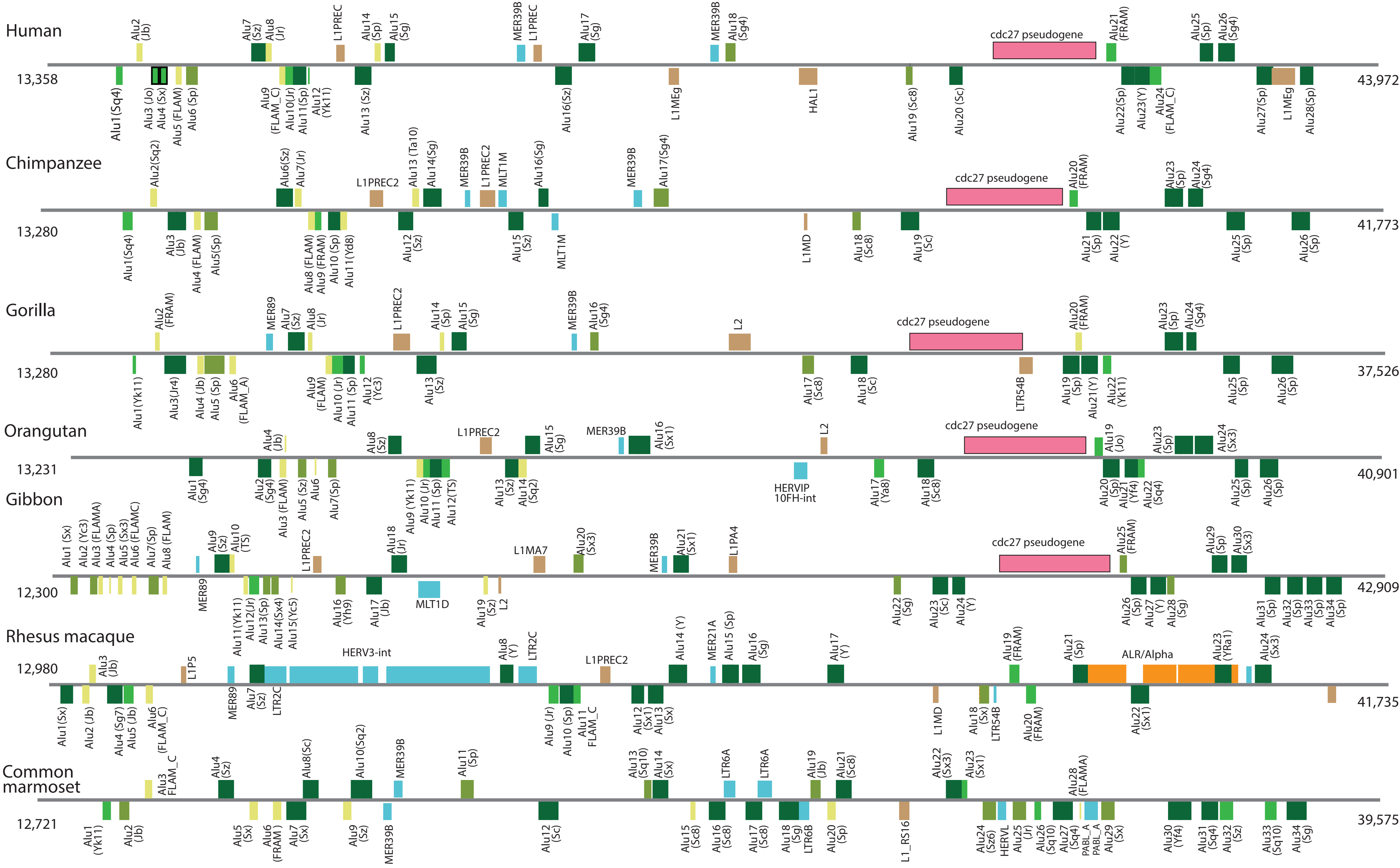

S2 Figure: Repeat elements in the IGS of different primates species. The IGS is shown as a grey line. Repeat elements are indicated above and below the IGS as follows: Alu elements (green boxes), LTRs (blue boxes), LINEs (brown boxes), and satellites (orange boxes), with the names alongside. The cdc27 pseudogene is shown as a pink box. Elements above the rDNA are on the forward strand; elements below are on the reverse strand. The start and end coordinates of the IGS are indicated.

Agrawal & Ganley, S2 Figure
