# Supplementary material for: The conservation landscape of the human ribosomal RNA gene repeats
Source: PLoS One. 2018 Dec 5;13(12):e0207531. doi: 10.1371/journal.pone.0207531 (PMC6281188; doi:10.1371/journal.pone.0207531)
Supplement: S3 Fig — A) Alignment of the human rRNA promoter region, which encompasses the upstream control element (UCE; brown box) and core control elements (CCE; green box) as indicated by the numbering below relative to the transcription start site. Bases that match human are in black, mismatches are in grey. B) Alignments of potential rRNA terminators (Sal boxes) in the human IGS. The name of the terminator is indicated on the top of each alignment, and the coordinates relative to the human rDNA sequence are indicated. The nucleotides that match the 11 bp human rRNA terminator consensus sequence (GGGTCGACCAG) (Haltiner et al. 1986) are in black, mismatches are in grey. Absence of a terminator is indicated by hyphens. Alignments corresponding to conserved regions identified in this study are shown in pink boxes, with the name of the corresponding conserved region indicated below. Numbering above the alignments refers to the positions in the human rDNA sequence. (PDF) [file pone.0207531.s010.pdf]

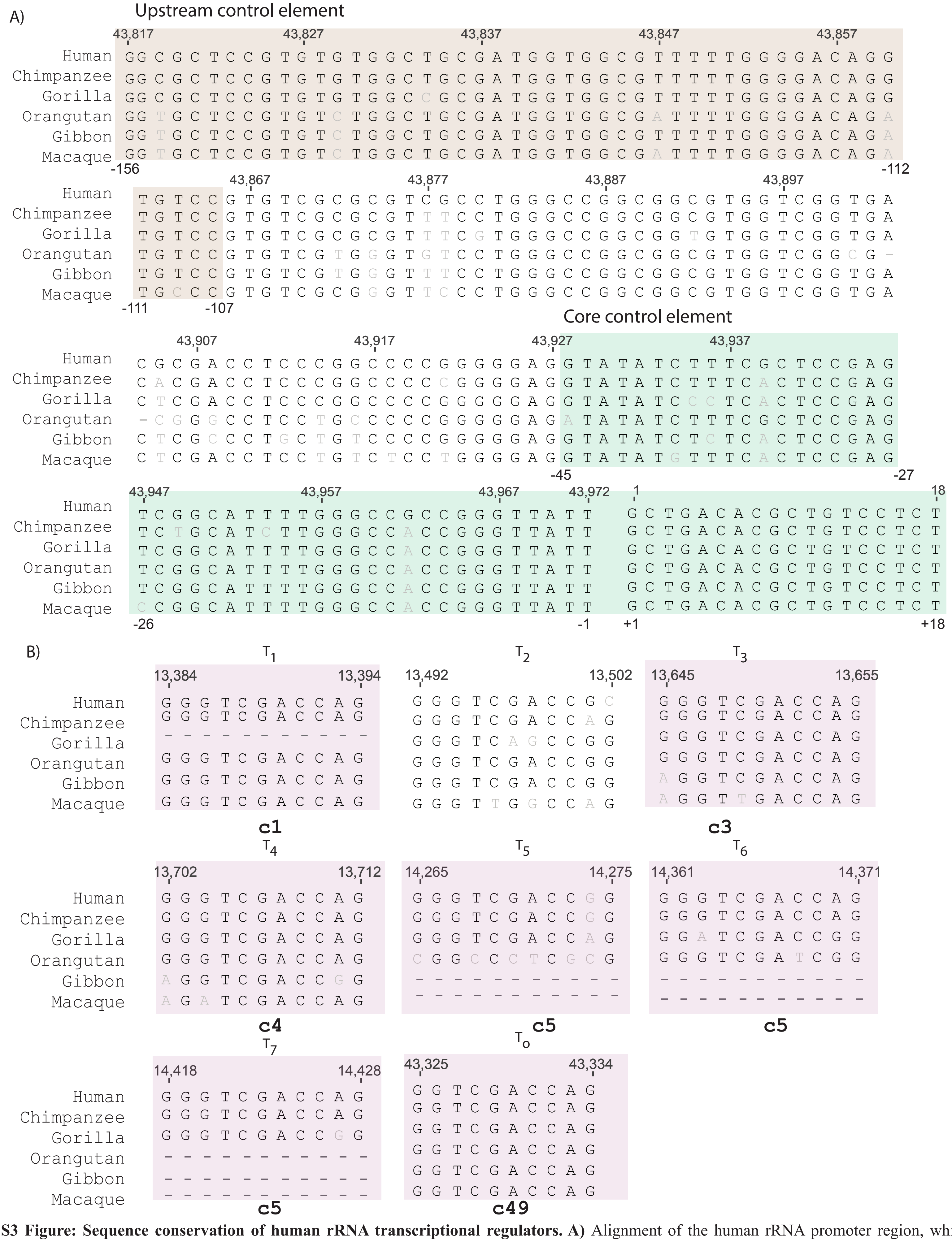

S3 Figure: Sequence conservation of human rRNA transcriptional regulators. A) Alignment of the human rRNA promoter region, which encompasses the upstream control element (UCE; brown box) and core control elements (CCE; green box) as indicated by the numbering below relative to the transcription start site. Bases that match human are in black, mismatches are in grey. **B)** Alignments of potential rRNA terminators (Sal boxes) in the human IGS. The name of the terminator is indicated on the top of each alignment, and the coordinates relative to the human rDNA sequence are indicated. The nucleotides that match the 11 bp human rRNA terminator consensus sequence (GGGTCGACCAG) (Haltiner et al. 1986) are in black, mismatches are in grey. Absence of a terminator is indicated by hyphens. Alignments corresponding to conserved regions identified in this study are shown in pink boxes, with the name of the corresponding conserved region indicated below. Numbering above the alignments refers to the positions in the human rDNA sequence.

Agrawal & Ganley, S3 Figure
