# Supplementary material for: The conservation landscape of the human ribosomal RNA gene repeats
Source: PLoS One. 2018 Dec 5;13(12):e0207531. doi: 10.1371/journal.pone.0207531 (PMC6281188; doi:10.1371/journal.pone.0207531)
Supplement: S4 Fig — Alignments of all 29 potential c-Myc binding sites in the human IGS are shown. Alignments corresponding to conserved regions identified in this study are shown in pink boxes, with the name of the corresponding conserved region indicated below. The coordinates of each c-Myc binding site relative to the human rDNA sequence are indicated on the top of each alignment. The nucleotides that are conserved with human are shown in black and that mismatches are in grey. Absence of an orthologous c-Myc binding site is indicated by hyphens. (PDF) [file pone.0207531.s011.pdf]

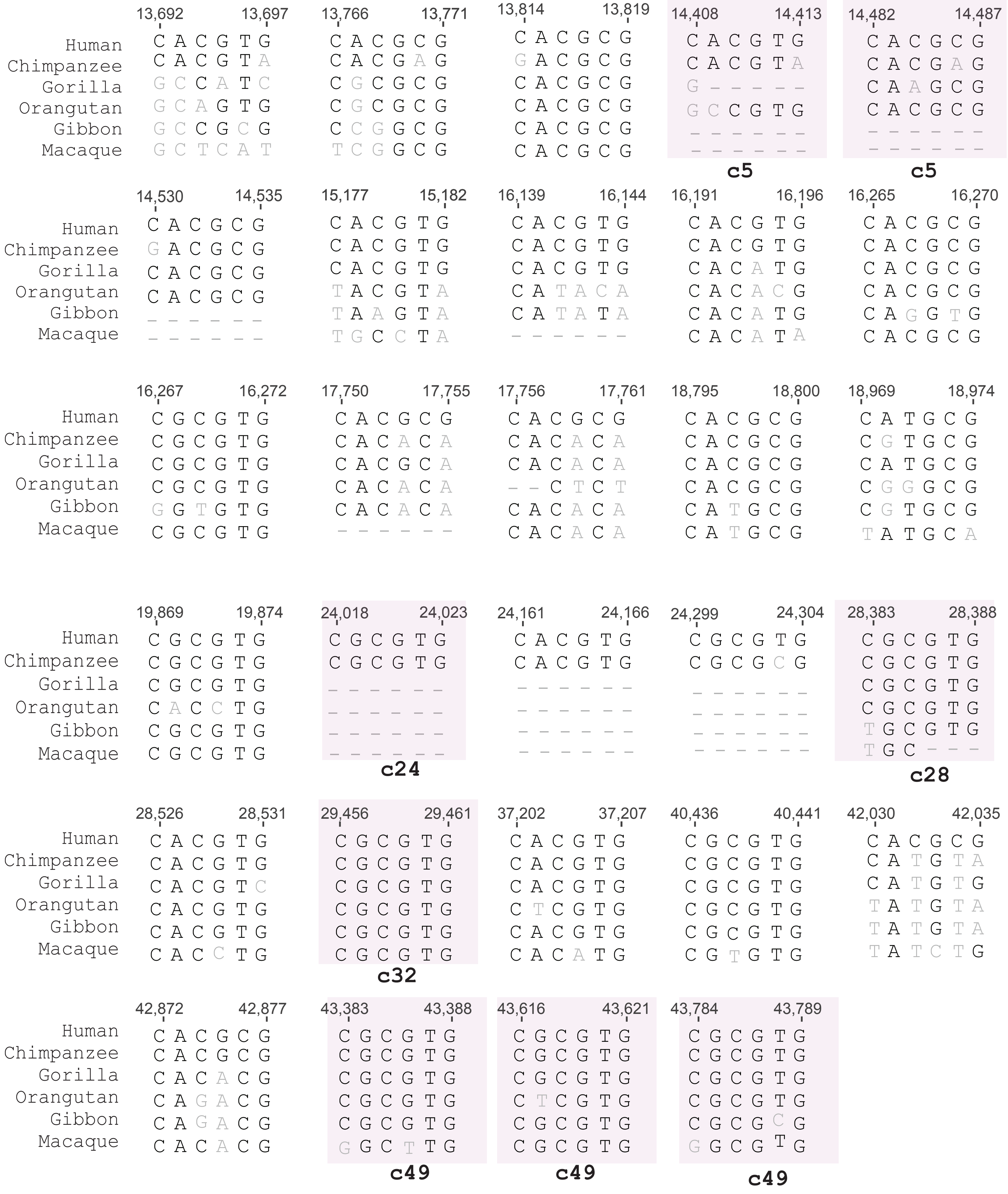

S4 Figure: Sequence conservation of potential c-Myc binding sites in the human IGS. Alignments of all 29 potential c-Myc binding sites in the human IGS are shown. Alignments corresponding to conserved regions identified in this study are shown in pink boxes, with the name of the corresponding conserved region indicated below. The coordinates of each c-Myc binding site relative to the human rDNA sequence are indicated on the top of each alignment. The nucleotides that are conserved with human are shown in black and that mismatches are in grey. Absence of an orthologous c-Myc binding site is indicated by hyphens.

Agrawal & Ganley,
S4 Figure
