# Supplementary material for: The conservation landscape of the human ribosomal RNA gene repeats
Source: PLoS One. 2018 Dec 5;13(12):e0207531. doi: 10.1371/journal.pone.0207531 (PMC6281188; doi:10.1371/journal.pone.0207531)
Supplement: S5 Fig — The human IGS with conserved regions (purple boxes) and Alu elements (green boxes) is shown at the top. The diagonal shaded region shows the position of the cdc27 pseudogene. Each cell line is separated by thick black lines. The black (plus strand) and red (minus strand) boxes represent CAGE tag signals. Long polyA(+) (green boxes) and polyA(-) (blue boxes) transcripts with FPKM value > 0.5 are shown. The arrowheads show the direction of transcription. Grey boxes represent polyA(+)and polyA(-) transcripts (depending on the lane the box is present in) with FPKM value < 1. The small RNA (< 200 bp) signals are shown as pink peaks with the scale (pink bracket on the left) representing the number of reads (negative values represent the reverse strand). The cell line is indicated to the left, although the CAGE stem cell data come from H9-hESC, not H1-hESC. (PDF) [file pone.0207531.s012.pdf]

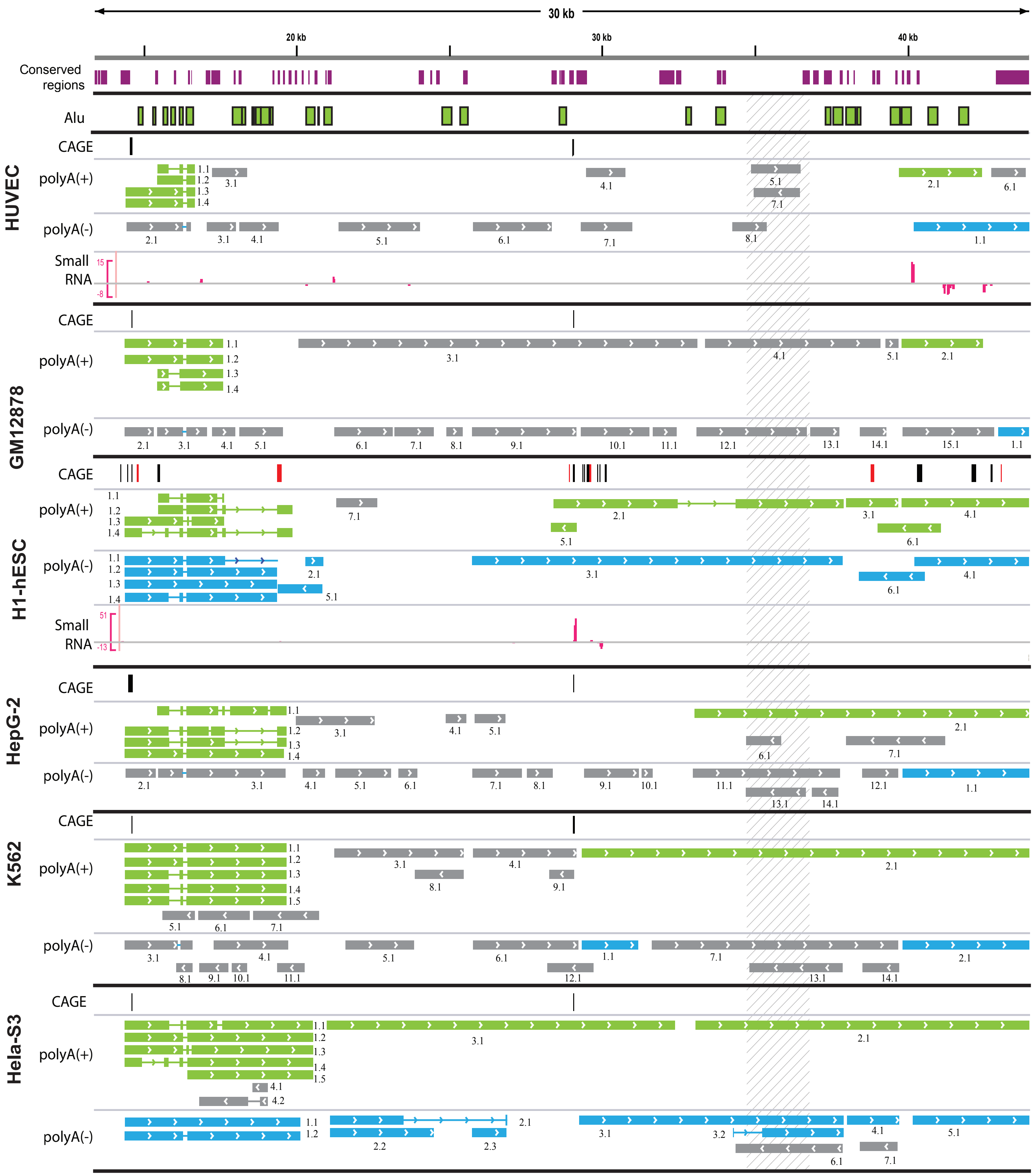

S5 Figure: The transcriptomic landscape of the human IGS in different cell lines. The human IGS with conserved regions (purple boxes) and Alu elements (green boxes) is shown at the top. The diagonal shaded region shows the position of the cdc27 pseudogene. Each cell line is separated by thick black lines. The black (plus strand) and red (minus strand) boxes represent CAGE tag signals. Long polyA(+) (green boxes) and polyA(-) (blue boxes) transcripts with FPKM value > 0.5 are shown. The arrowheads show the direction of transcription. Grey boxes represent polyA(+)and polyA(-) transcripts (depending on the lane the box is present in) with FPKM value < 1. The small RNA (< 200 bp) signals are shown as pink peaks with the scale (pink bracket on the left) representing the number of reads (negative values represent the reverse strand). The cell line is indicated to the left, although the CAGE stem cell data come from H9-hESC, not H1-hESC

Agrawal & Ganley,
S5 Figure
