# Supplementary material for: The conservation landscape of the human ribosomal RNA gene repeats
Source: PLoS One. 2018 Dec 5;13(12):e0207531. doi: 10.1371/journal.pone.0207531 (PMC6281188; doi:10.1371/journal.pone.0207531)
Supplement: S6 Fig — The scale at the top shows the position in the rDNA unit, and the start of the IGS is indicated by the pink vertical line. Purple boxes with purple shaded regions below represent the conserved regions. The position of cdc27 pseudogene is shown as a diagonally shaded region. Each row represents phastcon signal (pink boxes), the enrichment for active histone modifications (green signals), repressive histone modifications (red signals), CTCF (orange signals), RNA polymerase II (Pol-II; blue signals), CAGE peaks (black boxes), long polyA(+) transcripts (green boxes), and long polyA(-) transcripts (blue boxes). The scales on the left represent the levels of enrichment. Grey rows represent the absence of the data or no signal in the human IGS for the ENCODE dataset. (PDF) [file pone.0207531.s013.pdf]

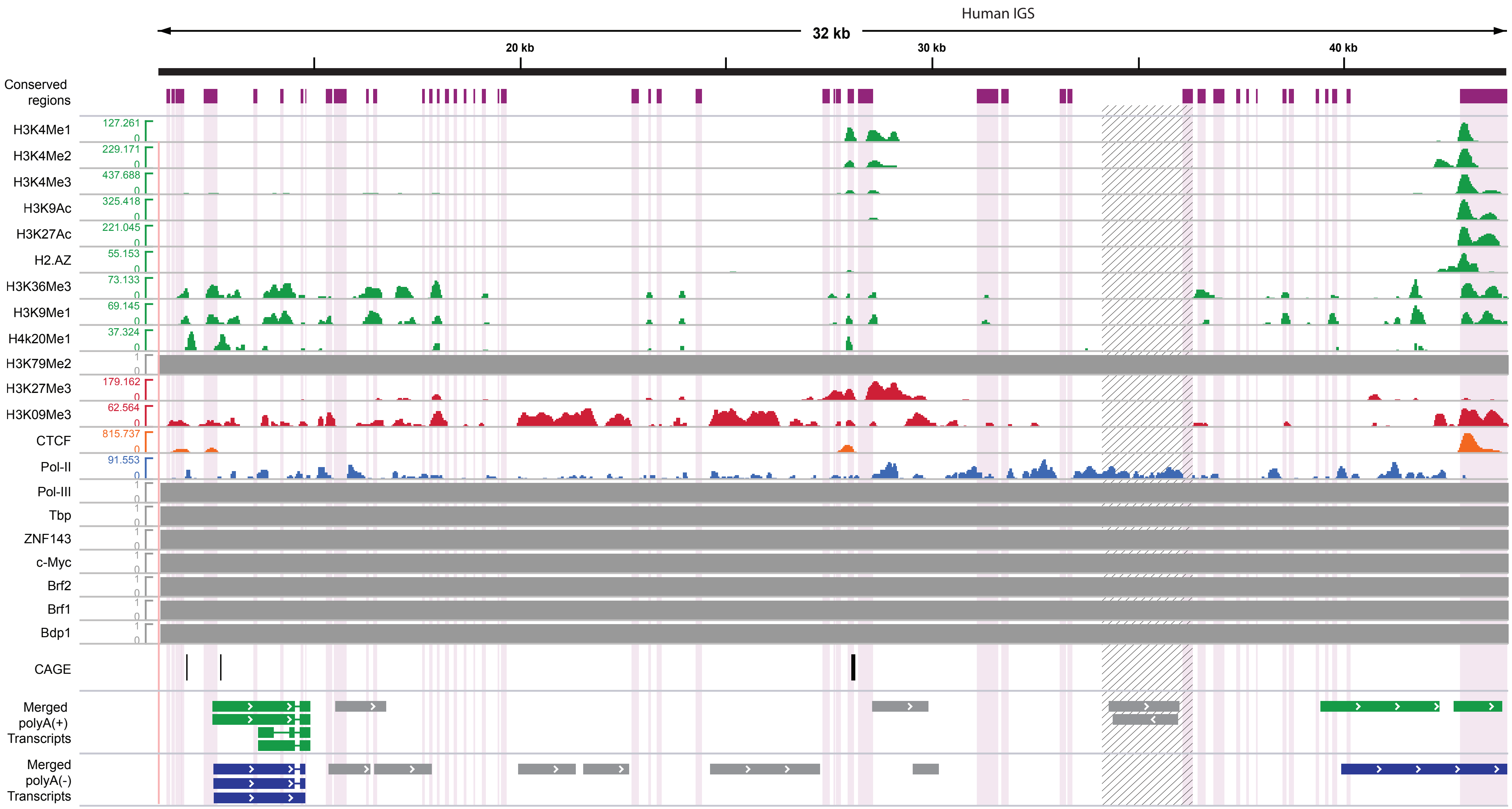

S6 Figure: Chromatin, transcription factor and transcript landscape of the IGS in the umbilical vein endothelial cell line, HUVEC. The scale at the top shows the position in the rDNA unit, and the start of the IGS is indicated by the pink vertical line. Purple boxes with purple shaded regions below represent the conserved regions. The position of cdc27 pseudogene is shown as a diagonally shaded region. Each row represents phastcon signal (pink boxes), the enrichment for active histone modifications (green signals), repressive histone modifications (red signals), CTCF (orange signals), RNA polymerase II (Pol-II; blue signals), CAGE peaks (black boxes), long polyA(+) transcripts (green boxes), and long polyA(-) transcripts (blue boxes). The scales on the left represent the levels of enrichment. Grey rows represent the absence of the data or no signal in the human IGS for the ENCODE dataset.

Agrawal & Ganley, S6 Figure
