# Supplementary material for: The conservation landscape of the human ribosomal RNA gene repeats
Source: PLoS One. 2018 Dec 5;13(12):e0207531. doi: 10.1371/journal.pone.0207531 (PMC6281188; doi:10.1371/journal.pone.0207531)
Supplement: S7 Fig — Figure as for S6 Fig, except that the blue signals represent transcription factors and RNA polymerases, and pink signals indicated small RNA transcripts. (PDF) [file pone.0207531.s014.pdf]

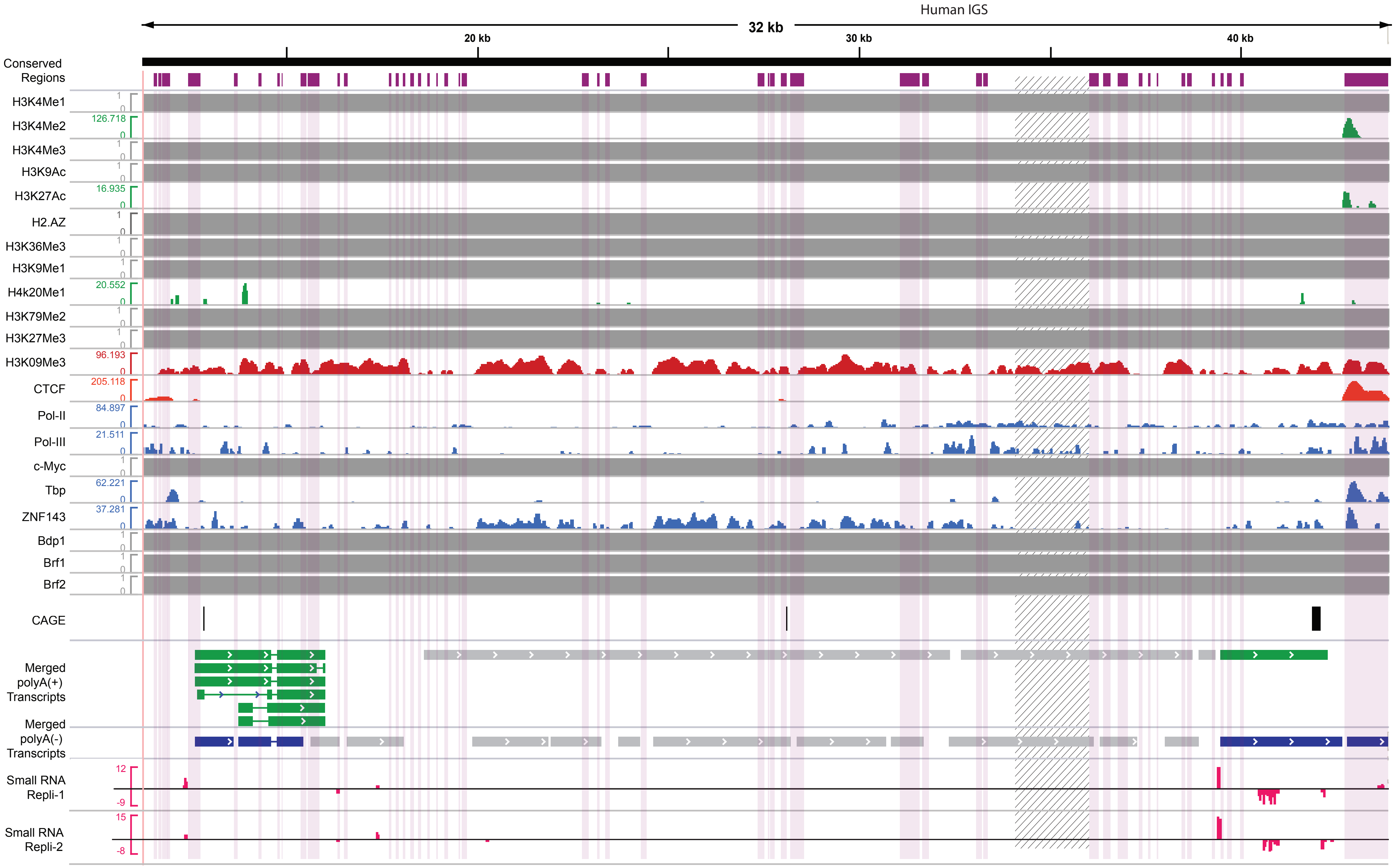

S7 Figure: Chromatin, transcription factor and transcript landscape of the IGS in the lymphoblastoid cell line, GM12878. Figure as for **S6 Figure**, except that the blue signals represent transcription factors and RNA polymerases, and pink signals indicated small RNA transcripts.

Agrawal & Ganley, S7 Figure
