# Supplementary material for: The conservation landscape of the human ribosomal RNA gene repeats
Source: PLoS One. 2018 Dec 5;13(12):e0207531. doi: 10.1371/journal.pone.0207531 (PMC6281188; doi:10.1371/journal.pone.0207531)
Supplement: S8 Fig — Figure as for S7 Fig. The CAGE data come from H9-hESC, not H1-hESC. (PDF) [file pone.0207531.s015.pdf]

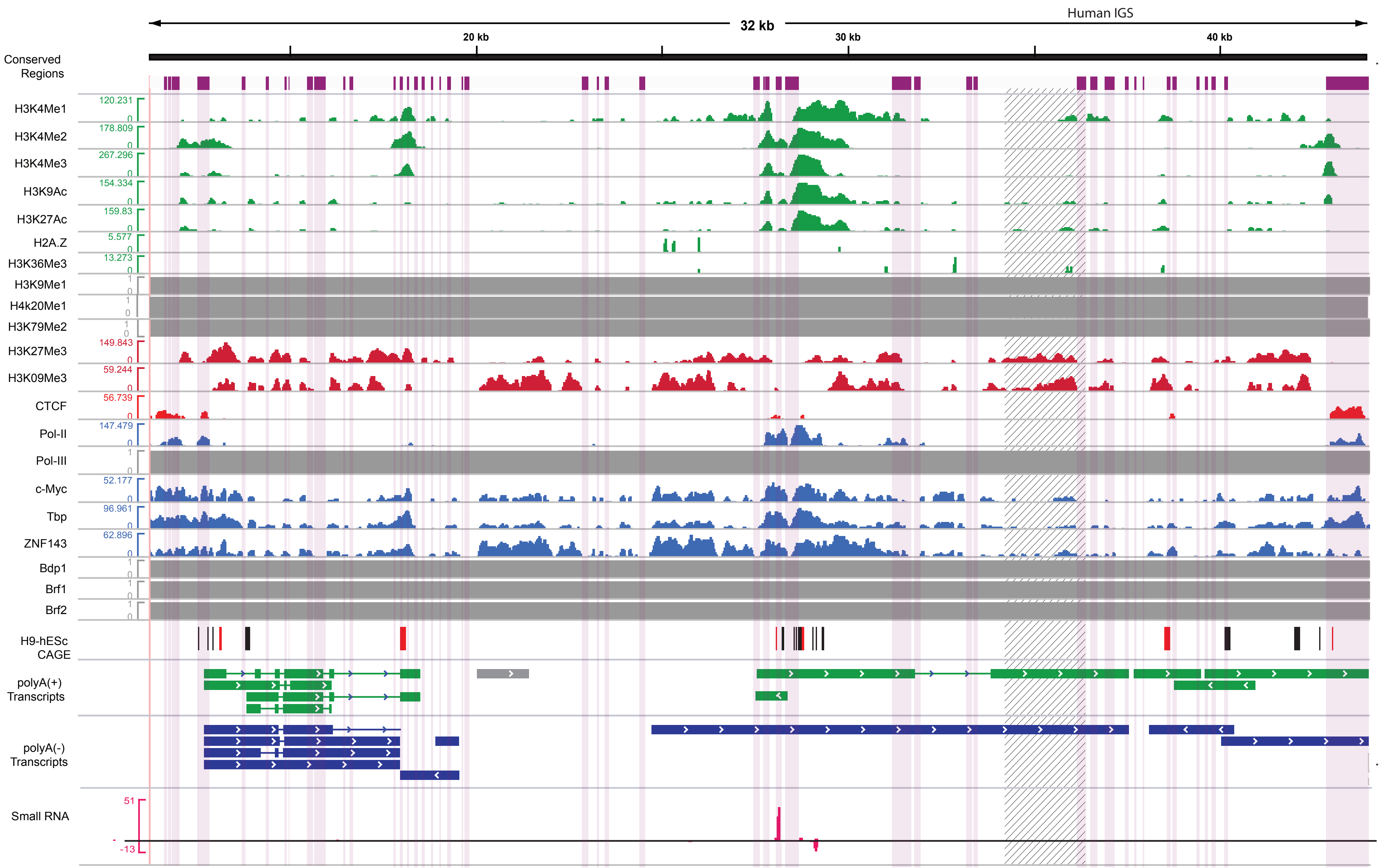

S8 Figure: Chromatin, transcription factor and transcript landscape of the IGS in the embryonic stem cell line, H1-hESC. Figure as for **Figure S7.** The CAGE data come from H9-hESC, not H1-hESC.

Agrawal & Ganley, S8 Figure
