# Supplementary material for: The conservation landscape of the human ribosomal RNA gene repeats
Source: PLoS One. 2018 Dec 5;13(12):e0207531. doi: 10.1371/journal.pone.0207531 (PMC6281188; doi:10.1371/journal.pone.0207531)
Supplement: S12 Fig — Figure as for S7 Fig. (PDF) [file pone.0207531.s019.pdf]

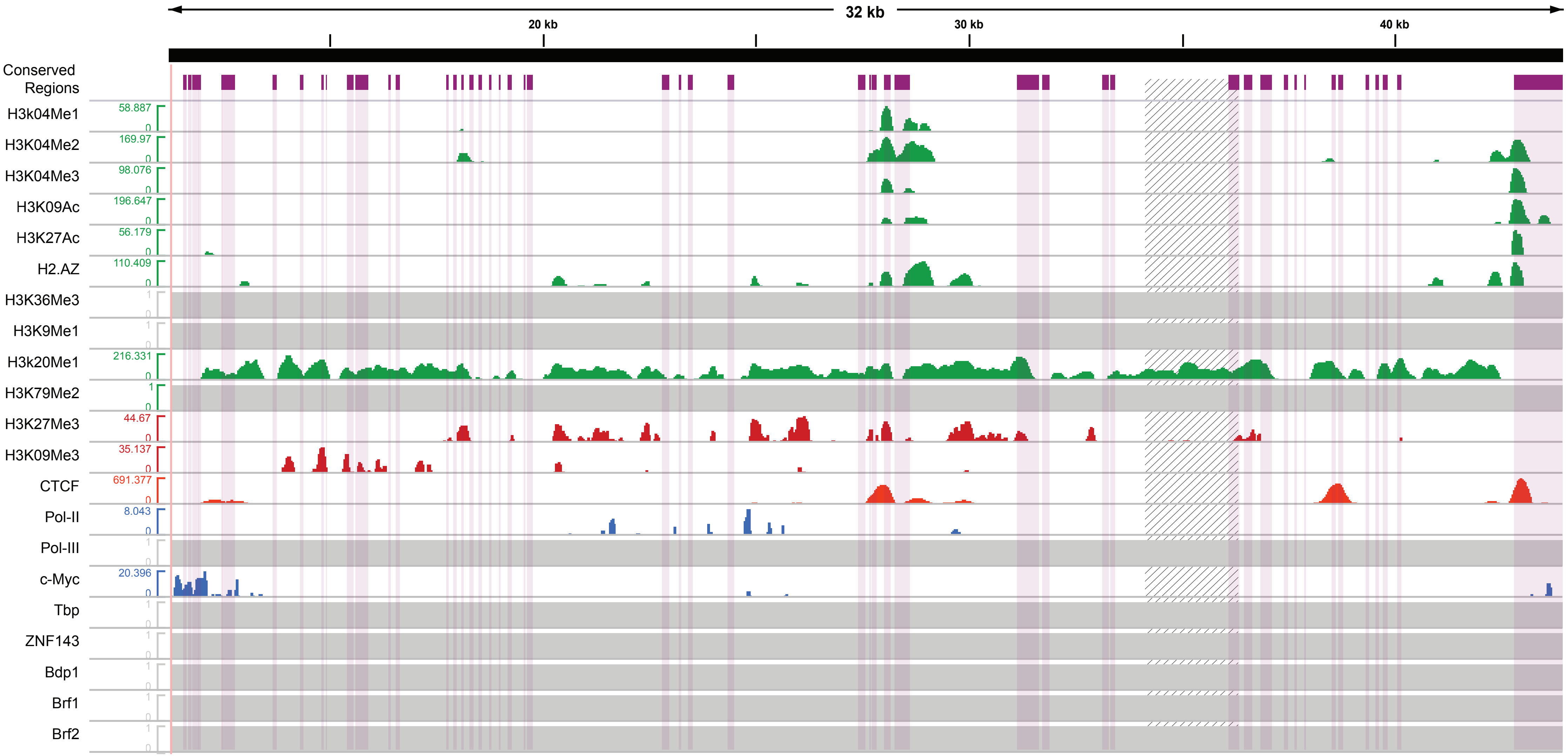

S12 Figure: Chromatin, transcription factor and transcript landscape of the IGS in the adenocarcinomic cell line, A549. Figure as for **Figure S7.**

Agrawal & Ganley,
S12 Figure
