# Supplementary material for: The conservation landscape of the human ribosomal RNA gene repeats
Source: PLoS One. 2018 Dec 5;13(12):e0207531. doi: 10.1371/journal.pone.0207531 (PMC6281188; doi:10.1371/journal.pone.0207531)
Supplement: S13 Fig — The segmentation states were obtained by merging histone modification, Pol II and CTCF peaks using Segway. The conserved regions (purple boxes) in the human IGS, also shown as grey shadows through the cell lines, are indicated at the top along with Alu elements (green boxes) and microsatellites (grey boxes). The diagonally shaded region represents the cdc27 pseudogene. Segmentation states for each cell line are boxed below, with the name of the cell line indicated to the left. The predicted states shown are: transcription start sites (TSS; green boxes), promoters (pink boxes), and enhancers (orange boxes). CAGE peaks are shown as black boxes (positive strand) and red boxes (reverse strand). The CAGE stem cell data come from H9-hESC, not H1-hESC. Long poly(A+) and poly(A-) transcripts with FPKM values > 1 are shown as green and blue arrows, respectively. Gray arrows show transcripts with FPKM < 1. The arrows indicate the direction of transcription. Small RNA peaks are shown in pink. Not all features have data available for all cell lines. (PDF) [file pone.0207531.s020.pdf]

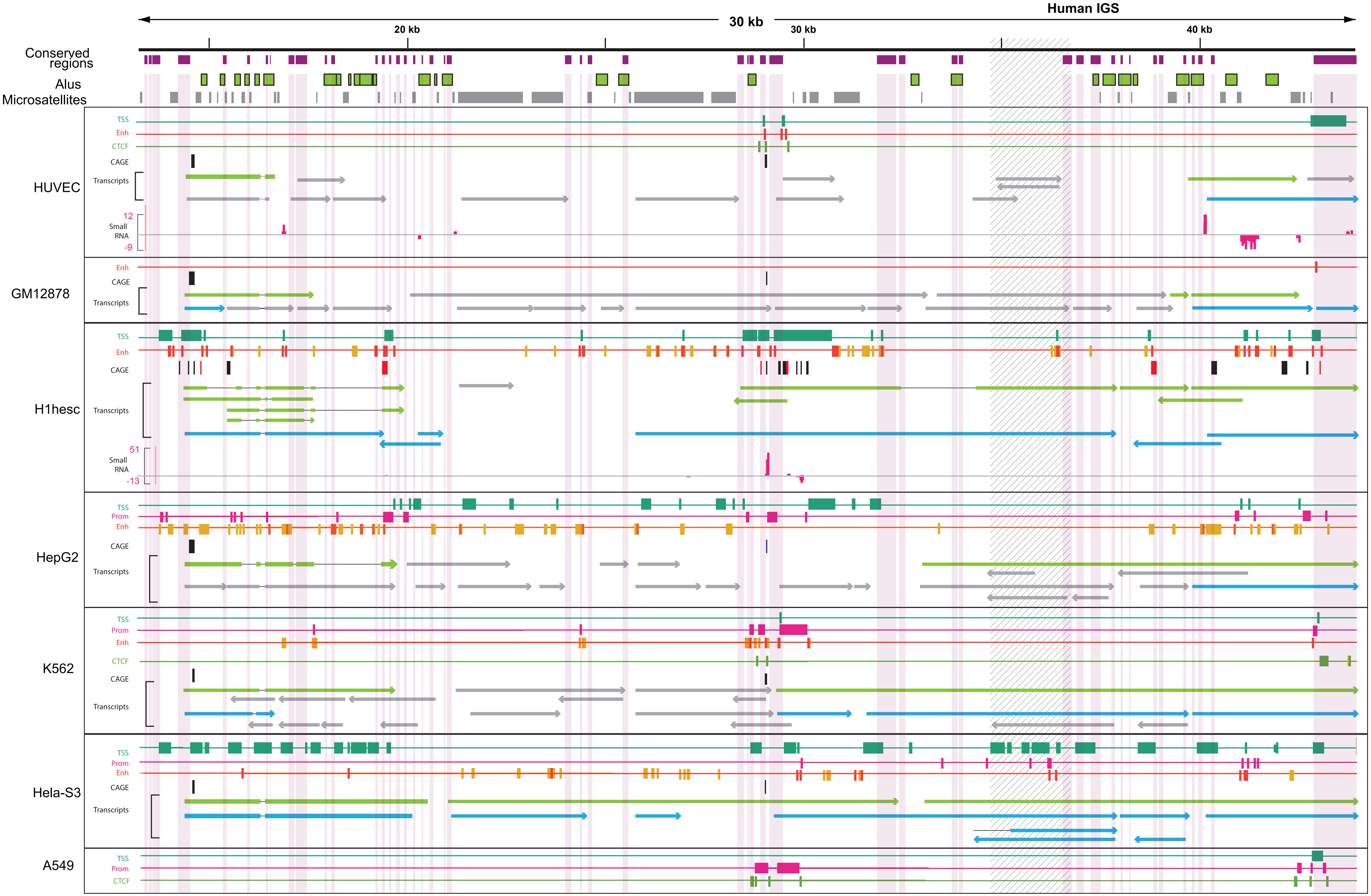

S13 Figure: Genomic segmentation showing functional annotation states in the human IGS. The segmentation states were obtained by merging histone modification, Pol II and CTCF peaks using Segway. The conserved regions (purple boxes) in the human IGS, also shown as grey shadows through the cell lines, are indicated at the top along with Alu elements (green boxes) and microsatellites (grey boxes). The diagonally shaded region represents the cdc27 pseudogene. Segmentation states for each cell line are boxed below, with the name of the cell line indicated to the left. The predicted states shown are: transcription start sites (TSS; green boxes), promoters (pink boxes), and enhancers (orange boxes). CAGE peaks are shown as black boxes (positive strand) and red boxes (reverse strand) . The CAGE stem cell data come from H9-hESC, not H1-hESC. Long poly(A+) and poly(A-) transcripts with FPKM values > 1 are shown as green and blue arrows, respectively. Gray arrows show transcripts with FPKM < 1.The arrows indicate the direction of transcription. Small RNA peaks are shown in pink. Not all features have data available for all cell lines.

Agrawal & Ganley, S13 Figure
