# Supplementary material for: The conservation landscape of the human ribosomal RNA gene repeats
Source: PLoS One. 2018 Dec 5;13(12):e0207531. doi: 10.1371/journal.pone.0207531 (PMC6281188; doi:10.1371/journal.pone.0207531)
Supplement: S14 Fig — Transcriptome assemblies were performed using stranded total (top half; orange colors) and unstranded polyA(+) (bottom half; green colors) RNA-seq data from different tissues of chimpanzee. The first (dark orange boxes) and seventh (dark green boxes) rows represent the consensus transcripts obtained by merging together the individual IGS transcripts (light orange/green boxes) from different tissues that are shown in the rows below the respective consensus rows. The names of the consensus transcripts are indicated underneath them. Tissue source and replicate number are indicated to the left. The direction of transcription is indicated by the arrowheads. The scale above shows the position in the chimpanzee rDNA IGS. (PDF) [file pone.0207531.s021.pdf]

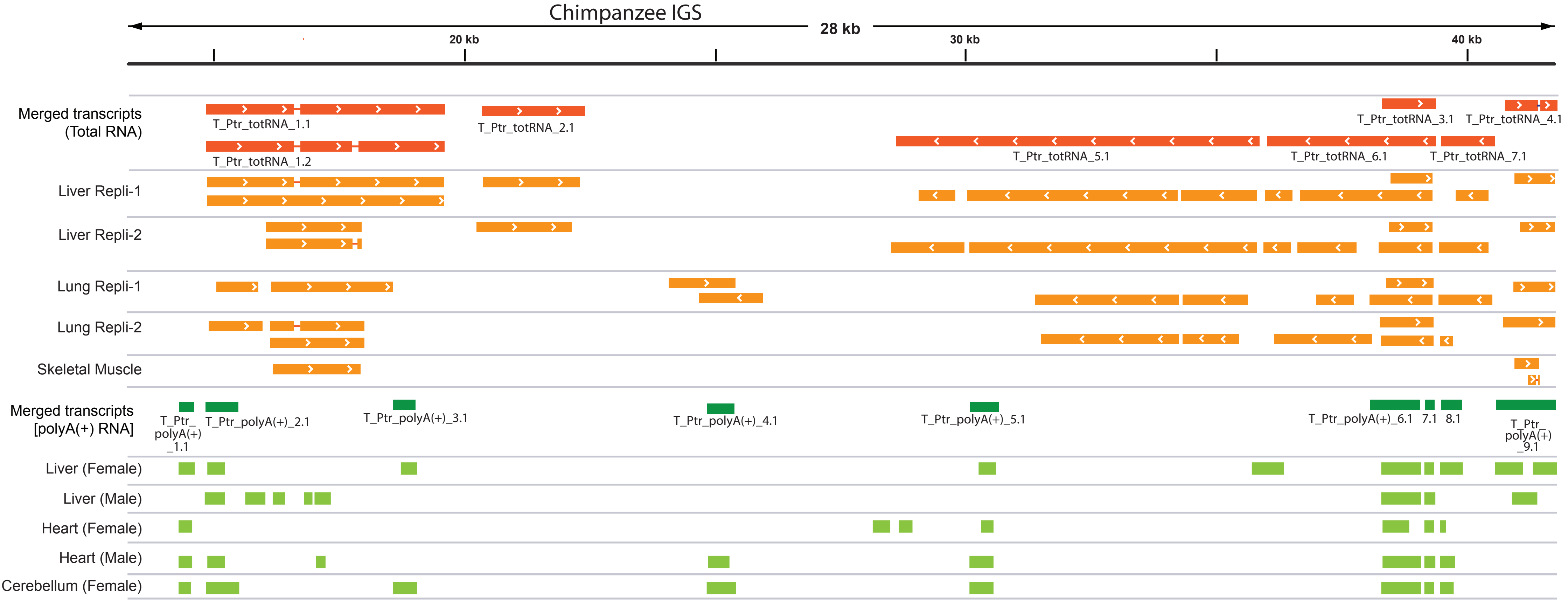

S14 Figure: Transcripts in the Chimpanzee IGS. Transcriptome assemblies were performed using stranded total (top half; orange colors) and unstranded polyA(+) (bottom half; green colors) RNA-seq data from different tissues of chimpanzee. The first (dark orange boxes) and seventh (dark green boxes) rows represent the consensus transcripts obtained by merging together the individual IGS transcripts (light orange/green boxes) from different tissues that are shown in the rows below the respective consensus rows. The names of the consensus transcripts are indicated underneath them. Tissue source and replicate number are indicated to the left. The direction of transcription is indicated by the arrowheads. The scale above shows the position in the chimpanzee rDNA IGS.

Agrawal & Ganley,
S14 Figure
