# Supplementary material for: The conservation landscape of the human ribosomal RNA gene repeats
Source: PLoS One. 2018 Dec 5;13(12):e0207531. doi: 10.1371/journal.pone.0207531 (PMC6281188; doi:10.1371/journal.pone.0207531)
Supplement: S16 Fig — Transcriptome assemblies were performed using unstranded polyA(+) RNA-seq data from the orangutan tissues indicated to the left. The first row represents consensus transcripts (dark green boxes) obtained by merging the individual IGS transcripts (light green boxes) from the different tissues (rows beneath). The names of the consensus transcripts are indicated next to them. The scale above shows the position in the orangutan rDNA IGS. (PDF) [file pone.0207531.s023.pdf]

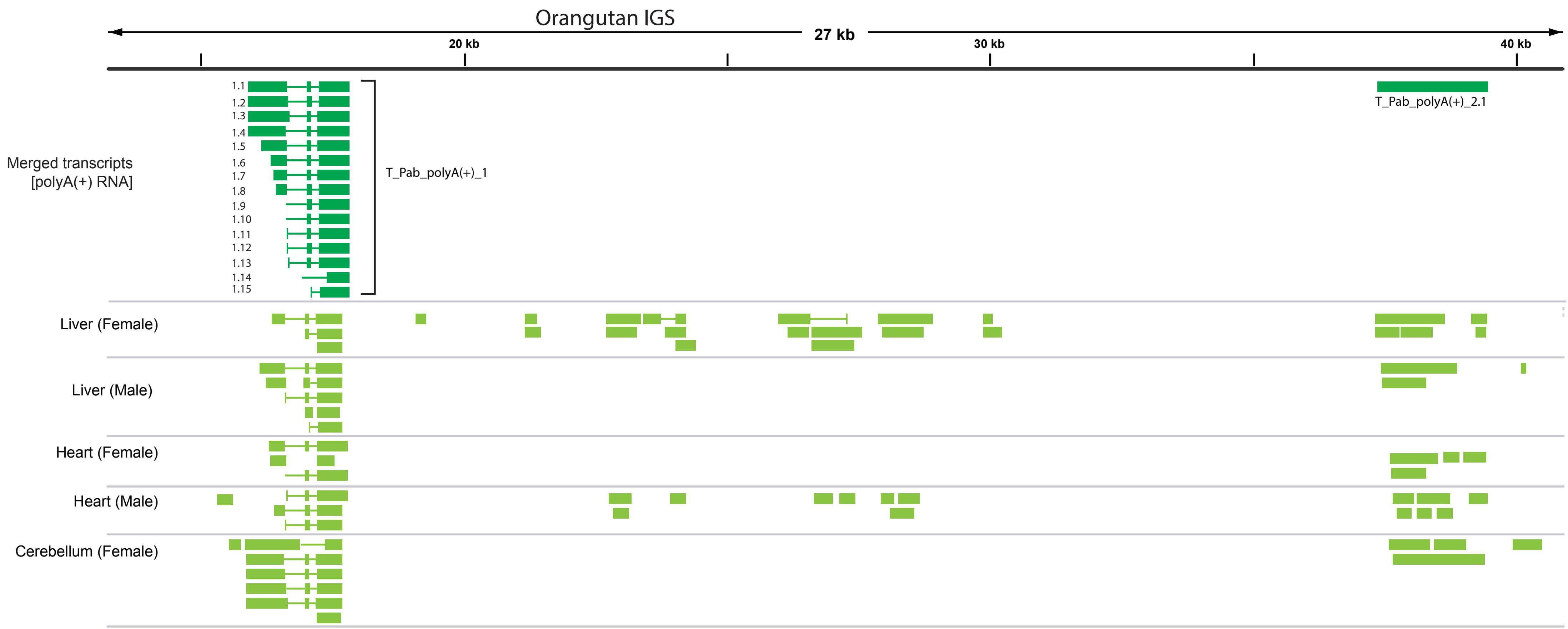


**S16 Figure: Transcripts in the orangutan IGS.** Transcriptome assemblies were performed using unstranded polyA(+) RNA-seq data from the orangutan tissues indicated to the left. The first row represents consensus transcripts (dark green boxes) obtained by merging the individual IGS transcripts (light green boxes) from the different tissues (rows beneath). The names of the consensus transcripts are indicated next to them. The scale above shows the position in the orangutan rDNA IGS.

**Agrawal & Ganley,**
**S16 Figure**
