# Supplementary material for: The conservation landscape of the human ribosomal RNA gene repeats
Source: PLoS One. 2018 Dec 5;13(12):e0207531. doi: 10.1371/journal.pone.0207531 (PMC6281188; doi:10.1371/journal.pone.0207531)
Supplement: S17 Fig — A) Abundance of the poly(A+) transcript splice variant from Zone-1 (represented by HUVEC poly(A+) transcript 1 in S5 Fig) that is shared between all cell lines. B) Abundance of the poly(A-) transcript from the promoter region (represented by HUVEC poly(A-) transcript 1 in S5 Fig) that is present in all cell lines. Abundances were calculated from RNA-seq data as FPKM (Fragments Per Kilobase of transcript per Million mapped reads; vertical axis) for the six cell lines (horizontal axis). The abundances represent the total expression from all rDNA units in the genome of the region of each transcript that is shared between all six cell lines. The error bars represent 95% confidence intervals for transcript abundance. (PDF) [file pone.0207531.s024.pdf]

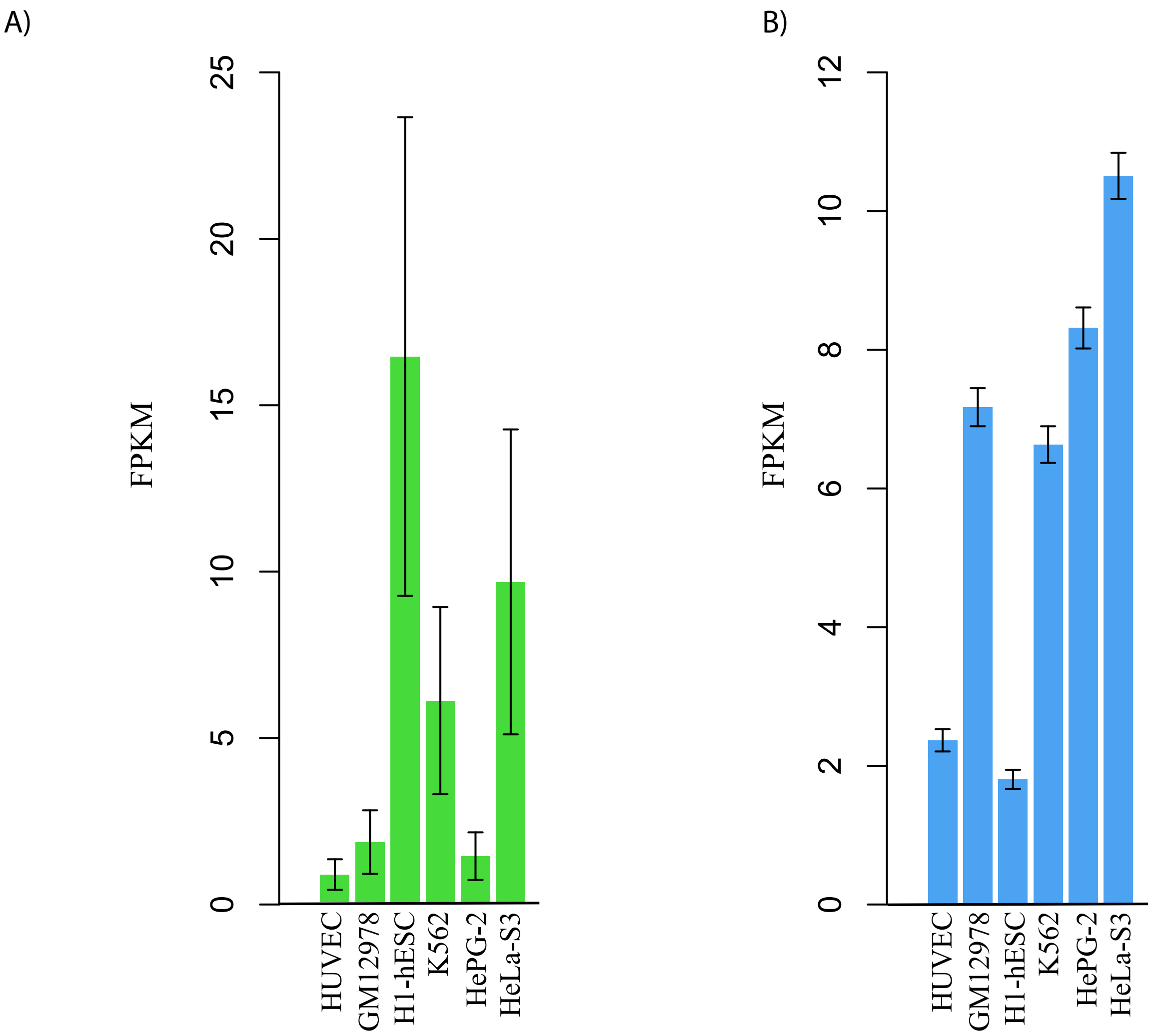


**S17 Figure: Quantification of the expression level of IGS transcripts. A)** Abundance of the poly(A+) transcript splice variant from Zone-1 (represented by HUVEC poly(A+) transcript 1 in **Figure S5**) that is shared between all cell lines. **B)** Abundance of the poly(A-) transcript from the promoter region (represented by HUVEC poly(A-) transcript 1 in **Figure S5**) that is present in all cell lines. Abundances were calculated from RNA-seq data as FPKM (**F**ragments **P**er **K**ilobase of transcript per **M**illion mapped reads; vertical axis) for the six cell lines (horizontal axis). The abundances represent the total expression from all rDNA units in the genome of the region of each transcript that is shared between all six cell lines. The error bars represent 95% confidence intervals for transcript abundance.

**Agrawal & Ganley,**
**S17 Figure**
